# Clinical exome sequencing for inherited retinal degenerations at a tertiary care center

**DOI:** 10.1038/s41598-022-13026-2

**Published:** 2022-06-07

**Authors:** Mythily Ganapathi, Amanda Thomas-Wilson, Christie Buchovecky, Avinash Dharmadhikari, Subit Barua, Winston Lee, Merry Z. C. Ruan, Megan Soucy, Sara Ragi, Joy Tanaka, Lorraine N. Clark, Ali B. Naini, Jun Liao, Mahesh Mansukhani, Stephen Tsang, Vaidehi Jobanputra

**Affiliations:** 1 Laboratory of Personalized Genomic Medicine, Department of Pathology & Cell Biology, Columbia University Medical Center, New York, NY USA; 2 Department of Ophthalmology, Columbia University, Edward S. Harkness Eye Institute, New York-Presbyterian Hospital, New York, NY USA; 3 College of Physicians and Surgeons, Columbia University Irving Medical Center, New York, NY USA; 4 Jonas Children’s Vision Care, Bernard & Shirlee Brown Glaucoma Laboratory, Columbia Stem Cell Initiative-Departments of Ophthalmology, Biomedical Engineering, Pathology & Cell Biology, Institute of Human Nutrition, Vagelos College of Physicians and Surgeons, Columbia University, New York, NY USA; 5 Precision Genomics Laboratory, Columbia University Irving Medical Center, 701 West 168th St., HHSC 1412, New York, NY 10032 USA

**Keywords:** Genetics, Genomics, Medical genomics

## Abstract

Inherited retinal degenerations are clinically and genetically heterogeneous diseases characterized by progressive deterioration of vision. This study aimed at assessing the diagnostic yield of exome sequencing (ES) for an unselected cohort of individuals with hereditary retinal disorders. It is a retrospective study of 357 unrelated affected individuals, diagnosed with retinal disorders who underwent clinical ES. Variants from ES were filtered, prioritized, and classified using the ACMG recommendations. Clinical diagnosis of the individuals included rod-cone dystrophy (60%), macular dystrophy (20%), cone-rod dystrophy (9%), cone dystrophy (4%) and other phenotypes (7%). Majority of the cases (74%) were singletons and 6% were trios. A confirmed molecular diagnosis was obtained in 24% of cases. In 6% of cases, two pathogenic variants were identified with phase unknown, bringing the potential molecular diagnostic rate to ~ 30%. Including the variants of uncertain significance (VUS), potentially significant findings were reported in 57% of cases. Among cases with a confirmed molecular diagnosis, variants in *EYS, ABCA4, USH2A, KIZ, CERKL, DHDDS, PROM1, NR2E3, CNGB1, ABCC6, PRPH2, RHO, PRPF31, PRPF8, SNRNP200, RP1, CHM, RPGR* were identified in more than one affected individual. Our results support the utility of clinical ES in the diagnosis of genetically heterogeneous retinal disorders.

## Introduction

Retinal diseases are a class of clinically and genetically heterogeneous disorders. To date, more than 300 genes have been described to cause non-syndromic or syndromic retinal degeneration, and the number is increasing (RetNet: https://sph.uth.edu/retnet/; Accessed 04/30/2022). A confirmed molecular diagnosis is important for clinical management, prognostic assessment and more importantly, treatment prospects such as gene therapy approaches are becoming increasingly available for Inherited Retinal Degenerations (IRDs) including the recently FDA-approved voretigene neparvovec-rzyl (Luxturna) for *RPE65* gene-specific Leber Congenital Amaurosis (LCA)^1^, and clinical trials are ongoing for gene therapies of several other IRD genes. It is likely that within the next few years we will see a rapid expansion of gene specific therapies and treatments for retinal disease^2^. Given this, the urgency to identify a molecular diagnosis in patients with retinal dystrophy has become critical for personalized medicine, patient care, and the prevention of vision loss in the affected individuals.

Many of the earlier methodologies used for detection of disease causing genetic variation in IRDs such as Sanger sequencing, polymerase chain reaction (PCR) or array-based methods, relied on targeted sequencing of a few genes (gene panels) or variants which were known to be causative for disease. These methodologies were targeted for specific gene/variation with low throughput and yields for molecular diagnosis. With the advent of Next-Generation Sequencing (NGS) based methodologies, analysis of millions of variants in the entire human genome became possible in a high-throughput way. This revolutionized the detection of disease associated variation and has led to the rapid expansion of our understanding of the molecular causes of human disease including IRDs, as well as our capability to identify the specific molecular cause of disease.

Given the incredibly heterogeneous nature of retinal disease, NGS based methodologies such as targeted panels or exome sequencing (ES) are ideal for identification of underlying molecular variants associated with this disease phenotype^3–11^. Targeted capture or virtual panels from ES are able to identify rare molecular variants within a specific subset of genes known to be associated with retinal disease.

Currently for IRD, both targeted panels and ES tests are routinely utilized by laboratories worldwide, and each has benefits and limitations. Targeted panels often have overall better coverage but lack the ability to detect variation in genes not included on the specific panel. The benefit of ES is that almost all the coding regions of the genome can be simultaneously analyzed. However, ES is limited to the coding exons and few bases of the splice junction of a wider variety of genes, but is not able to detect deep intronic variation which can often be included on specific targeted panels. Further, the capture might not be optimal for several regions, including genes of interest. Despite their benefits and limitations, the overall outcomes seem to be similar for both targeted panel and ES, with potentially significant molecular findings typically identified in > 50% of cases in large cohort studies^3–11^ (Supplemental Table 1). In these studies, targeted panel based testing was utilized for a significant subset or the entirety of the cohort, leading to a slight bias for that methodology over ES. Additionally, in many of the published studies, the description of the criteria used to determine a positive molecular diagnosis were lacking, with only few studies using a standardized classification metric for variants or specific evidence to classify these variants as pathogenic or likely pathogenic for disease, or confirm that the identified variant is just a rare benign variant identified in their test.

Here, we add to this body of knowledge with the results of ES performed in a clinical laboratory for 357 consecutive cases with retinal disorders. These individuals were ascertained at the ophthalmology clinic at Columbia University Irving Medical Center (CUIMC) and clinical ES test was performed in the Laboratory of Personalized Genomic Medicine at CUIMC. Variants identified in these cases were curated and interpreted based on the Variant Interpretation Guidelines described by the American College of Medical Genetics (ACMG)^12^.

## Results

### Demographics

**Figure 1:**
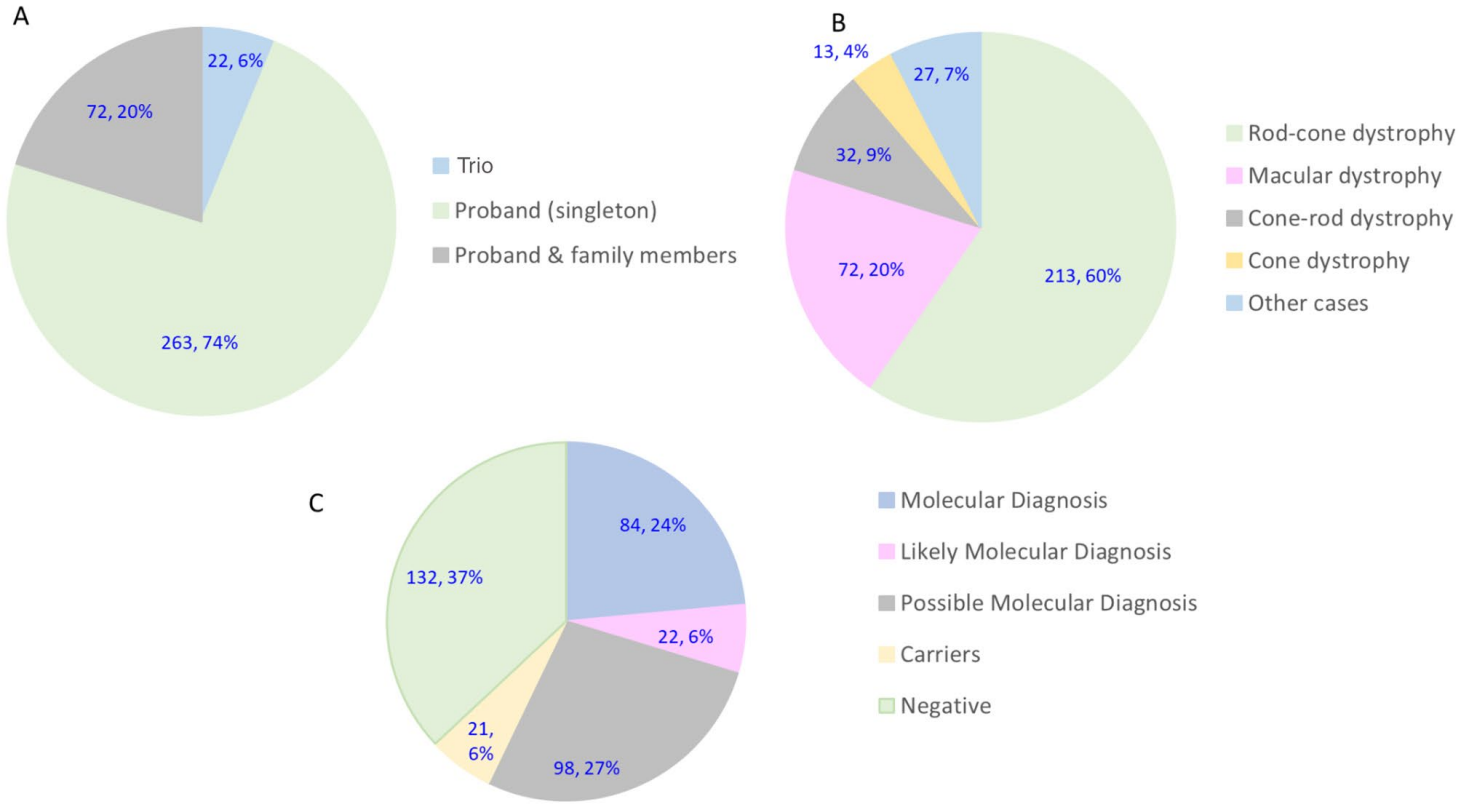
(**A**) Samples sent for clinical ES testing, (**B**) phenotypes seen in the cohort, (**C**) categorization of ES results.

In this study, 357 consecutive unrelated probands with retinal degeneration tested in our laboratory from 2012 to 2018, were included. The majority of ES samples were submitted as singletons (73.6%, 263/357; Supplemental Table 2). Trio cases with both parents accounted for 6.2% (22/357) of cases, and 10.6% (38/357) of cases were submitted as duos with one parent. For a subset of the duo cases, additional affected or unaffected non-parental first or second-degree family members were subsequently submitted to determine inheritance or clarify the reported results. The remaining cases (34/357; 9.5%) were proband samples that were submitted with non-parental first-degree affected or unaffected family members, which largely included siblings or children of the proband (Fig. 1A).

Of the 357 probands, 182 (50.9%) were male and 175 (49.1%) were female. The ages of the affected individuals at the time of testing ranged from 3 months to 92 years, with the median age of 52.5 years. Based on the phenotype categories described in “Methods”, the majority of the cases were referred with a clinical diagnosis classifiable under rod-cone dystrophy (213/357; 59.6%) followed by macular dystrophy (72/357; 20.2%), cone-rod dystrophy (32/357; 9.0%), “other” (27/357; 7.5%), and cone dystrophy (13/357; 3.6%) (Fig. 1B; Supplemental Table 2).

### Cohort selection

Referrals with stereotypic gene-specific phenotypes had undergone candidate gene testing. For instance, candidate gene sequencing was offered to referrals for purpose of “ruling-out” characteristic disorders such as X-linked retinoschisis, X-linked ocular albinism, X-linked retinitis pigmentosa, X-linked choroideremia, congenital stationary night blindness, enhanced S-cone syndrome, rod monochromacy, *RPE65*- or *LRAT*- or *CRB1*- related early onset retinal dystrophy, Bietti crystalline dystrophy, cone dystrophy with supernormal rods, gyrate atrophy, *MAK1*-related RP, late-onset retinal degeneration (L-ORD) *BEST1*, Doyne honeycomb retinal dystrophy, Malattia Leventinese or familial dominant drusen, maternally inherited diabetes mellitus and deafness (*MIDD),* choroiditis areata*,* and Stargardt group 1 cases. Other cases with phenotypes which can be caused by alterations in any of the multiple IRD genes were sent for ES.

### Exome sequencing test results

The reported ES testing results were classified in this study into four categories as described in the methods section. A molecular diagnosis was found in 23.5% of cases (84/357) (Fig. 1C; Table 1). A likely molecular diagnosis was made in 6.2% of cases (22/357), and a possible molecular diagnosis was made in an additional 27.4% (98/357) of cases. Negative results with no variants that potentially explained the clinical findings were reported for 132/357 (37%) of cases. In the remaining 21 cases (5.9%), a single Pathogenic or Likely Pathogenic variant was identified in a gene associated with autosomal recessive disease or a single X-linked variant was found in females. In these cases, we cannot exclude the presence of a second variant not detectable by ES such as a deep intronic variant or a CNV. In summation, we identified a clinically significant or potentially clinically significant molecular finding in a total of 204 (57.1%) of cases and classified 132 (37%) cases as Negative, with an additional 21 (5.9%) cases with a single clinically significant variant in autosomal recessive disease (carriers).

**Table 1:** Molecular diagnosis and likely molecular diagnosis in our cohort.

Phenotype	Molecular diagnoses (%)	Likely molecular diagnoses (%)	Potentially clinically significant findings (%)
Rod-cone	53 (63.1%)	13 (59.1%)	55 (56.12%)
Macular dystrophy	16 (19.3%)	6 (27.3%)	19 (19.2%)
Cone-rod	6 (7.2%)	3 (13.6%)	12 (12.1%)
Cone dystrophy	3 (3.6%)	0 (0%)	4 (4.0%)
Other	6 (7.2%)	0 (0%)	8 (8.1%)
Total	84	22	98

In total, 362 variants were reported in the 357 cases submitted for ES. Of these 362 variants, 209 (57.7%) were classified as pathogenic or likely pathogenic and 153 (42.2%) were classified as VUS at the time of analysis based on ACMG/AMP variant interpretation guidelines^12^.

### Cases with molecular diagnosis

Out of the 84 cases with molecular diagnosis, the highest rate of positive cases were those clinically identified as having rod-cone dystrophy, followed by macular dystrophy, cone-rod dystrophy, and cone dystrophy (Table 1). Six cases were classified as “other” phenotypes and included one case each of ocular albinism, *CACNA1F* associated disorder, and two cases each of Choroideremia and Pseudoxanthoma Elasticum.

Proband only cases comprised the majority (67/84; 79.7%) of the cases with a molecular diagnosis. Of the remaining cases, 8 were submitted as trio cases and 9 were submitted as duos. Among the 84 resolved cases, 40 (47.6%) were found to have homozygous or compound heterozygous variants in autosomal recessive disease, 33 (39.28%) had a heterozygous variant in autosomal dominant disease, and 11 (13.0%) individuals were hemizygous for a X-linked variant.

Several genes were identified in multiple independent cases with confirmed molecular diagnoses, with variants found most frequently in *RPGR*, *PRPH2* and *RHO* (see Table 2). The nine *RPGR* variants identified were present in the GA rich ORF15 (exon 15) which has poor sequence coverage in our ES platform. For all positive cases with *RPGR* variant in ORF 15, except one, we have confirmed the ORF15 variants by using amplicon based NGS methodology.

**Table 2:** Most frequently identified genes in the dataset (by number of families).

Gene	No. of cases	Inheritance	Phenotype association
*RPGR*	9	X-linked	Cone-Rod Dystrophy, Macular Degeneration, Retinitis Pigmentosa
*PRPH2*	6	AD, AR	Leber Congenital Amaurosis, Macular Dystrophy, Retinitis Pigmentosa, Choroidal Dystrophy
*RHO*	5	AD, AR	Night Blindness, Retinitis Pigmetosa, Retinitis Punctata Albescens
*ABCA4*	4	AR	Cone-Rod Dystrophy, Fundus Flavimaculatus, Retinal Dystrophy, Retinitis Pigmentosa, Stargardt
*EYS*	4	AR	Retinitis Pigmentosa
*KIZ*	3	AR	Retinitis Pigmentosa
*PRPF8*	3	AD	Retinitis Pigmentosa
*USH2A*	3	AR	Retinitis Pigmentosa, Usher Syndrome

A likely molecular diagnosis was made in 22 cases, all of which had two or more pathogenic or likely pathogenic variants identified in a gene associated with autosomal recessive inheritance. This included multiple cases with two or more variants of unknown phase in *ABCA4* (5 cases), *USH2A* (6 cases), *EYS* (3 cases), single cases with variants in *CNGA3, CNGB1, COL18A1, COQ2, NPHP4, PCARE, PDE6B*, and *RPE65.* These were largely proband only cases (19/22; 86.4%), two duo cases in which one parent was unavailable for testing, and a single case with proband and one child with unknown clinical phenotype submitted for testing.

### Potentially clinically significant molecular findings

In addition to the 84 cases with confirmed molecular diagnoses and 22 with a likely molecular diagnosis, an additional 98 cases had potentially clinically significant molecular findings, which includes two heterozygous variants in a gene with autosomal recessive inheritance (two VUS or one pathogenic/likely pathogenic variant along with a VUS variant), a VUS in a gene with autosomal dominant inheritance pattern, and X-linked variants in hemizygous males classified as VUS.

The 98 cases were comprised of 56.6% cases with rod-cone dystrophy, followed by macular dystrophy, cone-rod dystrophy, cone dystrophy (Table 1). Eight cases with potentially clinically significant findings were classified based on phenotypes as “other” and included cases of glaucoma, cystoid macular edema with lamellar hole, and pre-symptomatic individuals with significant family history of retinal disease.

Among these 98 cases with potentially clinically significant molecular findings, only 5 were submitted as trios. Of these 5 trio cases, 4 had two VUS *in trans* and one had a homozygous VUS. Eleven were submitted as duo cases. In 5 of these cases, a VUS was identified in a gene with autosomal dominant inheritance pattern and 6 cases identified two heterozygous or one homozygous variant in a gene with autosomal recessive inheritance pattern. In 4 of the 5 cases with 2 variants in a gene with autosomal recessive inheritance patterns, at least one of the identified variants met pathogenic or likely pathogenic criteria, and in 2 of these 5 cases both variants were classified as pathogenic. The remaining 83 cases were submitted as singleton proband samples.

In total, we identified variants in 85 different genes. The most common variants identified in our cohort were variants in *ABCA4*, *EYS*, *PRPH2*, *RPGR*, and *USH2A*, which each identified as potentially clinically significant molecular findings in more than 10 independent cases (Table 3). Variants were identified in 65.6% of cone-rod dystrophy, 64.0% of rod-cone dystrophy, 59.7% of macular dystrophy, and 30.8% of cone dystrophy cases.

**Table 3:** Genes in which variants were identified by phenotype.

Phenotype	No. of cases	No. of cases with at least 1 P/LP	Genes identified with P/LP variants
Rod-cone	212	94	*ABCA4, AHI1, BEST1, BBS1, CEP290, CNGB1, CNGB3, COQ2, EYS, FAM161A, IFT140, IMPG2, KIZ, KLHL7, MYO7A, NR2E3, PCARE, PCDH15, PDE6B, PRPF8, PRPF31, RHO, RP1, RPGR, SNRNP200, TOPORS, USH2A*
Macular dystrophy	72	28	*ABCA4, CNGA3, CRX, EFEMP1, PDE6C, PROM1, PRPF8, PRPH2, RDH12, RP1L1, RPGR, RS1*
Cone-rod	32	12	*ABCA4, CDHR1, GUCY2D, NPHP4, PCARE, PROM1, PRPH2, RPGR, TULP1*
Cone dystrophy	13	4	*CACNA2D4, GUCA1A, MAK, OPN1MW*
Other	29	9	*ABCC6, CACNA1F, CHM, EYS, GPR143, LCA5, SLC38A8*

P/LP Pathogenic/Likely Pathogenic.

### Cases with “other” phenotypes in the cohort

Of the cases with clinical diagnoses that fell within our “Other” category, 65.4% had at least one variant identified with potential clinical significance, including 3 (11.5%) that had a molecular diagnosis. Molecular findings in this group included variants in genes such as *ABCC6*, *CHM*, *GPR143*, *SLC38A8*, *ASB10*, and *NR2E3*.

### Cases with multiple molecular findings

In 3 cases, molecular findings in multiple genes potentially related to the clinical phenotype were identified. In one case, we reported two VUSs in two different autosomal dominant disease associated genes (Case 124; Supplemental Table 2), one case was reported with 2 heterozygous variants of unknown phase in an autosomal recessive disease gene in addition to a single VUS in an autosomal dominant disease gene (Case 93) and one case in which two heterozygous variants in three different autosomal recessive genes were identified (Case 26). Each of these cases are considered potentially clinically significant findings with a possible molecular diagnosis.

The first case (Case 124) has a VUS in two autosomal dominant genes, *SNRNP200* and *PRPF8*. Both genes affect RNA splicing and are associated with autosomal dominant retinitis pigmentosa. Fundus examination showed numerous white dots in the inferior retina. Intraretinal pigment was noted more severely in the inferior retina than the superior retina (Supplemental Fig. 1A). ERG examination was abnormal and consistent with a rod-cone dystrophy. These findings together show a likely diagnosis of retinitis pigmentosa. However both genes cause a very similar phenotype.

The second case (Case 93) showed two pathogenic variants in the gene *ABCA4*, phase unknown and one heterozygous VUS in *CRX*. Both genes can cause cone-rod dystrophy. The patient had a central bull’s eye atrophy in both maculae. Drusenoid deposits were noted in both eyes (Supplemental Fig. 1B). ERG was normal which is consistent with Stargardt Group 1. The phenotype is more consistent with *ABCA4.*

The last case (Case 26) had two likely pathogenic heterozygous variants identified in *CNGB1*, phase unknown; two heterozygous VUS in *KCNV2*, phase unknown; and one heterozygous likely pathogenic and one heterozygous VUS in *GALC,* phase unknown. Fundus exam showed dense intraretinal pigment migration encroaching on the arcade (Supplemental Fig. 1C). ERG examination was not completed for this patient. Given the three candidates, *CNGB1* fits the phenotype the best as it would cause the peripheral intraretinal pigment migration and the classical retinitis pigmentosa symptoms the patient reported.

### Outcomes for ophthalmic disease

The ophthalmology records were reviewed to assess the outcomes of the ES testing on affected individuals and their families. The specific outcomes due to their sequencing results fell into 4 categories (1) approved for Luxturna (FDA approved gene-therapy), (2) enrolled/currently being enrolled in an interventional clinical trial, (3) correction of previously miscounseled inheritance pattern/reproductive risk, and (4) confirmed cascade testing of family members. Overall, 28 of the 357 cases (7.9%) were identified as having at least one of the four outcomes (Table 4). Of those with a molecular diagnosis 5 cases out of 84 (5.95%) were enrolled or in the process of being enrolled in an interventional clinical trial due to their sequencing results. Out of the 22 cases with a likely molecular diagnosis, 1 patient (4.5%), and her affected sibling, were able to get phase testing confirmed at a later date and have successfully completed Luxturna enrollment. ES results allowed for correction in counseling regarding inheritance and reproductive risk in 19 out of the 357 cases (5.3%). Cascade familial testing was done for 3 out 357 cases (0.8%).

**Table 4:** Outcomes for ophthalmic disease.

ES category	Cases approved for Luxturna	Cases enrolled/currently enrolling for intervention clinical trial	Cases provided with corrective counseling for inheritance	Cases with known family member cascade testing
Molecular Diagnosis	2	5	9	5
Likely Molecular Diagnosis	1	0	1	0
Possible Molecular Diagnosis	0	0	13	0

In some cases variants were identified in genes causing syndromic IRD including multiple cases with biallelic variants in ciliopathy genes, Pseudoxanthoma Elasticum, ocular albinism, and Coenzyme Q10 deficiency (Supplemental Table 3). Review of the charts provided additional clinical data confirming the molecular diagnosis for some of these cases including polycystic kidneys in a patient with biallelic variants in *CEP290* (Case 155), kidney failure, motor problems, and intellectual disability in a patient with a homozygous *AHI1* variant (Case 221), and chronic kidney disease in a patient with two heterozygous variants of unknown phase in the *NPHP4* gene (Case 16).

### Secondary findings (ACMG v1 & v2)

Secondary findings in the genes recommended by ACMG^13,14^ were reported in 4 out of 357 cases (1.12%) and included pathogenic or likely pathogenic variants in *BRCA1*, *APOB*, *TNNT2,* and *LDLR* genes.

## Discussion

The extensive clinical and genetic heterogeneity of disorders of hereditary retinal degeneration have led to large scale adoption of high throughput NGS based methodologies such as gene panels, ES, and more recently whole genome sequencing (GS) which can test for a large set of genes at once. Several large NGS based studies have been published with potentially significant variants identified in 41–76% of cases, with variable yields depending on the specific disease subtype^3–11^ (Supplemental Table 1). The majority of these studies (6/8; 75%) utilized NGS with either targeted capture or virtual gene panel, one (12.5%) utilized ES and a stand-alone test for RPGR (ORF 15), and one (12.5%) utilized ES in a subset of probands and a targeted capture panel in the remainder of the cohort. Of these studies, only one^6^ used ACMG guidelines to interpret identified variants, and accordingly, in others, aspects such as the rate of confirmed molecular diagnosis versus the likely molecular diagnosis which includes two pathogenic variants with unknown phase, and whether confirmed cases also included ones with VUS or two variants with unknown phase, are unclear. The classification of variants based on ACMG guidelines and categorization of case results using a clearly defined scoring system utilized in the current study provides a consistent way to compare the diagnostic yields across different cohort datasets.

By comparison, in this study we completed clinical ES in a series of 357 individuals with inherited retinal dystrophy including specific diagnoses of rod-cone dystrophy, macular dystrophy, cone-rod dystrophy or cone dystrophy. Using ACMG guidelines to interpret all variants identified in our laboratory, we confirmed a molecular diagnosis in 23.5% of cases, a likely molecular diagnosis in an additional 6.2% of cases, and a possible molecular diagnosis in 27.4% of cases, for a total of 57.1% of cases with either a confirmed molecular diagnosis or potentially clinically significant findings. This is comparable to the yield of clinical whole exome sequencing for other disease subtypes^15^, as well as the rate of molecular findings in other previously published cohorts with IRD (Supplemental Table 1).

Some differences between the previous studies and ours include the cohort demographics, specific clinical phenotypes, previous testing performed before inclusion in the current study, availability of familial samples for variant phasing, and NGS methodology used. The coverage and depth of reads for retinal genes may differ between targeted gene panels and ES. One of the limitations of our exome method is the inability to detect CNVs which are implicated in many retinal disorders and there are multiple studies which have underscored the importance of these variants in retinal disorders^16,17^. However, unlike pediatric patients with neurodevelopmental disorders, where chromosomal microarray testing is a standard of care, eye disorder patients do not undergo standard chromosomal microarray testing, even though the literature now supports prevalence of CNVs in this cohort. CNVs along with deep intronic variants may explain a part of the missing heritability in our patient population. In at least one study, for 17.3% cases (n = 45) only a single likely causative variant in autosomal recessive disease^5^ was identified. Additional testing in a subset of these cases identified a likely causative deep intronic variant in 6 cases, a likely causative small deletion or duplication in an additional 4 cases, and 1 case in which a canonical splice variant was detected by the second testing methodology that was missed by ES likely due to low coverage^5^. Similar to these findings, we identified a single variant in autosomal recessive disease in 21 (5.9%) cases. In 9 of these cases, single variants were identified in *ABCA4, EYS,* or *USH2A*, which all have previously reported and/or well known pathogenic intronic variants which would not have been detected by ES. While there are few recurrent small deletions or duplications associated with some of the genes identified in these 21 samples, small private CNVs or larger CNVs cannot be excluded. Follow-up testing including GS was recommended in each of these cases, although results of those tests if conducted were not available for further analysis.

The highest rate of potentially clinically significant findings as well as the highest rate of molecular diagnosis was found in individuals who were referred with clinical findings of rod-cone dystrophy, in which 64% of individuals had a potentially clinically significant finding, and 24.6% received a molecular diagnosis. Variants in disease-associated genes were reported in 59.7% of cases with clinical findings consistent with macular dystrophy, with 22.2% of individuals receiving a molecular diagnosis. Individuals with cone dystrophy were returned potentially significant variants in 53.8% of cases, and molecular diagnoses were reported in a total of 23.1%. Interestingly, individuals with clinical findings of cone-rod dystrophy were returned the highest percentage of potentially clinically significant findings (65.6%), but molecular diagnosis was only identified in 15.6% of cases. This may reflect the small number of individuals with cone-rod dystrophy (n = 32), although it may also reflect fewer variants with significant evidence for pathogenicity.

Across all disease subtypes in our cohort, 11% of the cases had two variants identified in a disease associated with autosomal recessive inheritance with unknown phase. While follow-up testing is not always a viable option, pursuing additional testing methodologies, targeted variant testing, or segregation studies in families may ultimately resolve some of these cases. Furthermore, samples submitted to our lab were not routinely reanalyzed, so reanalysis and/or re-classification of these variants with current evidence may result in different classifications.

Limitations of our observational retrospective study include that a majority of the cases submitted were singleton samples restricting the phase information when variants in AR genes were identified, incomplete targeted familial testing for the identified variants as most of the cases were of adults, limited review of the post-ES clinical testing results which were triggered by a ES finding, and lack of systematic review on clinical follow-ups and genotype–phenotype correlation for cases with molecular findings. Further, cases analyzed before the availability of large population databases such as prior to ExAC in 2015, were not re-reviewed using the updated allele frequency information. Additionally, our clinical WES analysis approach is primarily focused on finding variants in known IRD genes and might potentially miss novel candidate gene variants.

GS is often being used on a research or clinical basis. It has the benefit of simultaneously detecting exonic variation as well as intronic variation, CNVs, and in some cases repeat expansions and mitochondrial DNA variation. While GS is currently not often utilized as a first-tier test for diagnosis of genetic disease, it has the potential to change in the near future as technology becomes more widely available and our understanding of non-exonic genetic variation in disease improves.

## Methods

Retrospective review of clinical records and genetic testing results between 2012 and 2018 was performed. Patients were seen and evaluated at the Edward S. Harkness Eye Institute at Columbia University Irving Medical Center. Informed consent was waived due to the minimal risk conferred to the patients and the retrospective nature of the study design as per Columbia University Institutional Review Board-approved protocol AAAR8743. All procedures were reviewed and in accordance with the tenets of the Declaration of Helsinki. All patients underwent a full ophthalmic examination which includes assessment of best-corrected visual acuity (BCVA), acquisition of autofluorescence imaging, optical coherence tomography (OCT) scans and in most cases, full-field electroretinogram (ffERG) testing. Cases with gene-specific phenotypes were sent for candidate gene sequencing. For the rest, peripheral blood samples of the proband and the family members (if available) were sent for ES in a CLIA certified lab in the Laboratory of Personalized Genomic Medicine at the Columbia University Irving Medical Center (CUIMC). Written informed consent was obtained from all patients for the ES testing. Follow-up targeted variant testing in additional family members was completed, post-hoc, if the samples were available at a later time point. Prior to 2016, most referral cases were initially sent for a retinal gene panel testing and the ones that were negative for a causal variant that was consistent with the disease phenotype by panel sequencing were reflexed to clinical ES test. Since 2016, except for gene-specific phenotypes, the other cases were directly sent for ES testing.

### Categorization of cases based on the phenotype

Based on the clinical examination, cases were broadly categorized into five phenotypic classifications based on the shared degenerative features and disease trajectories. The rod-cone dystrophy classification encompasses disorders characterized by initial pan-retinal rod degeneration with subsequent cone involvement typified by conditions such as LCA, retinitis pigmentosa and related disorders. The cone-rod dystrophy classification includes severe, early-onset loss of cone function that is manifested on ffERG resulting in progressive and rapid loss of central vision. The cone dystrophy classification includes disorders involving selective degeneration of cones such as achromatopsia and macular dystrophies include degenerative disorders confined to the posterior pole such as Stargardt disease or pattern macular dystrophy. The “other” category included cases of myopic degeneration, choroideremia, ocular albinism, glaucoma, familial drusen, pseudoxanthoma elasticum, etc.

### Clinical exome sequencing

For clinical ES, libraries were prepared from genomic DNA from the proband and the parents using Agilent SureSelectXT (Human All Exon v.5 + UTRs) capture kit according to the manufacturers’ protocol. Paired-end sequencing was performed on the Illumina HiSeq 2500 platform (average coverage of 100x). The sequence data was aligned [human genome reference sequence (GRCh37/hg19)] and annotated using Nextgene (version 2.3; Softgenetics, LLC. PA) software. Variant filtering and annotation were performed using an in-house developed pipeline and reviewed as part of the clinical workflow for constitutional clinical ES. The WES variants were categorized into various bins: (1) All variants that had been reported as pathogenic, (2) All variants in genes with associated phenotypes, and (3) All variants in genes not associated with phenotypes. These three bins were then filtered at 1% population frequency using 1000 genomes, Exome Variant Server^18^ and were used to generate a total of six separate outputs. Each of these output had variants in different tabs: disruptive variants (frameshift, stopgain, canonical splice site), missense variants, two or more heterozygous variants per gene (potential compound heterozygous variants), De Novo variants (for trios), Unique variants (variants only present in the proband and not seen in our database) and homozogyous/hemizygous variants. Additional population databases such as ExAC and gnomAD were used for variant prioritization as they became available.

Identified variants were assessed for the phenotype match by means of a bi-weekly case discussion meeting with the clinical team. Variants were classified using the guidelines from the ACMG and the Association of Molecular Pathology (AMP)^12^. Variants classified as pathogenic, likely pathogenic and VUS were reported in the test results. Heterozygous pathogenic/likely pathogenic variants in autosomal recessive disease associated genes (carriers) were also reported with a recommendation for a reflex to Genome Sequencing (GS) or copy number variant (CNV) test at an outside provider since these tests are currently not performed at our laboratory. Routine re-analysis of ES data is currently not part of our constitutional case analysis workflow in the clinical laboratory, hence other than special instances most cases have not been re-analyzed.

### RPGR ORF15 variants: amplicon based sequencing

*RPGR* ORF15 testing was performed on selected samples, which were negative by clinical ES test and met clinical indication for *RPGR* ORF15 testing. *RPGR* ORF15 variant detection was performed using a long range PCR, amplicon based NGS and analysis method. For this testing the genomic region chrX:38144742-38146527 (hg19) corresponding to the *RPGR* ORF15 region was amplified by PCR using the following primers, ORF15F 59-GTATGATTTTAAATGTGATCGCTTGTCAGAG and ORF15R 59-AAGGCATTTAAATTGTCTGACTGGCCATAATC^19^. PCR was performed using standard methods using Platinum SuperFi PCR Master Mix (Invitrogen Cat.# 12358-010).

The 1.7 kb PCR product was fragmented into smaller sized DNA, typically 150–200 bp, purified and end-repaired following 3’adenylation. Double stranded sequencing adaptors were ligated to both ends of this DNA. The library was loaded onto the MiSeq instrument (Illumina) for cluster generation and sequencing. Analysis of the sequence reads was performed by NextGENE software (SoftGenetics, LLC. Pennsylvania). For data analysis, the “consolidation” tool in NextGENE software was used, which merges overlapping sequences to give a consensus sequence in place of all the original reads that are in the subregion. This method is recommended for datasets that have a high depth of coverage in the raw reads, when there is a need to correct sequence reads such as those in repeat regions and difficult to align repeat rich regions. The resulting variant calls from the original reads and the consolidation output were evaluated for validity, based on variant allele fractions, gender of the proband and if the variant was seen in ES reads from the ORF15 region.

### Categorization of cases based on the results of ES test

Based on the ES results, the laboratory classified the cases into four categories. (1) Cases in which a pathogenic or likely pathogenic variant in an autosomal dominant (AD) disease gene, an X-Linked (XL) pathogenic or likely pathogenic variant in hemizygous or homozygous state, or in cases in which two pathogenic or likely pathogenic variants were identified in autosomal recessive (AR) disease *in trans,* in a disease gene with clinical phenotype match were classified as a “Molecular Diagnosis”. (2) Cases in which two pathogenic or likely pathogenic variants explaining the phenotype were identified with unknown phase were categorized as “Likely Molecular Diagnosis”. (3) Cases with two heterozygous variants with unknown phase in an AR disease gene (two VUS or one pathogenic/likely pathogenic variant along with a VUS variant), or cases in which a VUS was identified in an AD or XL gene were categorized as “Possible Molecular Diagnosis” (4) Remaining cases in which no variants potentially explaining the clinical phenotype were identified were categorized as “Negative”.

## Supplementary Information

Supplementary Information 1.

Supplementary Information 2.

Supplementary Information 3.

Supplementary Information 4.

## Data Availability

All variants reported during this study are included in this published article including supplementary information files. The raw next-generation sequencing data used to identify the variants reported herein cannot be made publicly available for reasons of patient confidentiality and in compliance with HIPAA regulations. Qualified researchers or clinicians may apply for access to these data pending institutional review board approval.
