# Supplementary material for: Clinical exome sequencing for inherited retinal degenerations at a tertiary care center
Source: Sci Rep. 2022 Jun 7;12:9358. doi: 10.1038/s41598-022-13026-2 (PMC9174483; doi:10.1038/s41598-022-13026-2)

**Supplemental figure 1:** Fundus autoflourescence imaging for patients with multiple molecular findings.


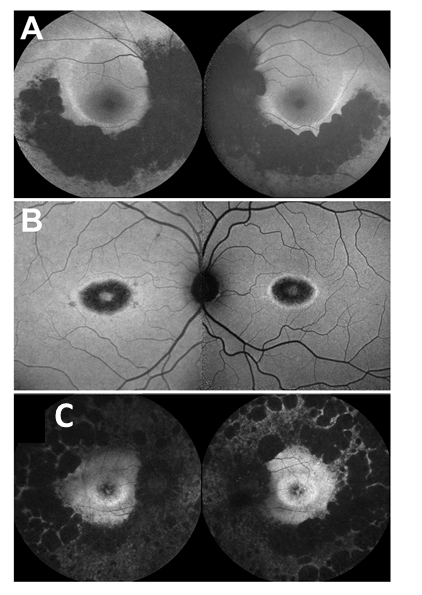

Supplement: Supplementary file 1 — Supplementary Information 1. [file 41598_2022_13026_MOESM1_ESM.docx]
