# Supplementary material for: Clinical exome sequencing for inherited retinal degenerations at a tertiary care center
Source: Sci Rep. 2022 Jun 7;12:9358. doi: 10.1038/s41598-022-13026-2 (PMC9174483; doi:10.1038/s41598-022-13026-2)
Supplement: Supplementary file 2 — Supplementary Information 2. [file 41598_2022_13026_MOESM2_ESM.docx]

**Supplemental table 1:** Comparison of diagnostic yields for inherited retinal disorders in published studies

| **Study** | **No. of probands** | **Phenotype distribution** | **Testing Methodology** | **Variants analyzed** | **Clinically significant or potentially clinically significant molecular finding** | **PMID** |
| --- | --- | --- | --- | --- | --- | --- |
| Wang, 2019 | 568 | 36% with inherited retinal degeneration, 27% with RP, 10% with macular degeneration, 9% with LCA, 7% with CORD, 6% with retinoschisis, 5% with STGD, 1% with CSNB and 1% with Bietti crystalline corneoretinal dystrophy | NGS panel (126 genes) | SNVs, small indels | 52% (293/568) | 31106028 |
| Carss, 2017 | 722 | 43% with RP, 14% with retinal dystrophy, 7% CORD, 6% with STGD, 5% with MD, 5% with Usher syndrome | ES (n=72), GS (n=605), both ES and GS (n= 45) | SNVs, small indels, SVs for GS cases | 56% (404/722) | 28041643 |
| Haer-Wigman L, 2017 | 266 | 49% with RP, 16% with cone or macular dystrophy, 11% with CORD, 6% with LCA | ES with virtual panel of genes (?) | SNVs, small indels, CNVs | 52% (137/266) | 28224992 |
| Wang, 2018 | 319 families | 69% with RP, 12% with CORD, 3% with Usher syndrome, and remainder 16% with LCA, CSNB, STGD, etc | ES (n=91), 441 NGS panel (n=228), Sanger sequencing for RPGR ORF15; NGS panel (441 genes) | SNVs, small indels, CNVs | 41.4% (132/319); Panel: 41.2% (94/228); ES: 33% (30/91) | 30029497 |
| Huang X, 2015 | 179 families | phenotype data n=99 (solved cases); 67% with RP, 10% with US, 6% with CRD, 6% with LCA, 5% with SD,3% with BCD, 2% with CSNB and 15 with ESCS | ES with gene panel (164 genes) | SNVs, small indels | 55.3% (99/179) | 25356976 |
| Stone, 2017 | 424 families | NA | ES | SNVs, small indels, CNVs | 43% (182/424) | 28559085 |
| Ellingford, 2016 | 537 | 47% with RP, 15% with LCA/early onset rod-cone dystrophy, 9% with STGD/MD, 7% with Usher syndrome, 7% with CORD, 5% Achromotopsia/cone dystrophy, 1% with suspected syndromic ciliopathy, 1% with FEVR, 1% with CHM, 8% with other indications | NGS panel (105 genes) | SNVs, small indels | 51% (271/537) | 27208204 |
| Carrigan, 2016 | 309 families | 39.4% with RP, 13.4% with STGD/MD/FFM, 3.5% with cone+cone rod dystrophy, 4.2% with CHM, 11.3% with Usher syndrome, 3.9% with LCA/EOSRD, 2.8% with Retinoschisis, 20.4% with other indications. | ? genes NGS panel | SNVs, small indels | 57% (176/309) | 27624628 |
| This study | 357 | 60% with RP, 20% with MD, 9% with CORD, 4% with Cone dystrophy, and remainder 8% cases with LCA/Myopia/ CHM/ Glaucoma/ Familial Drusen | standard ES; long range PCR followed by NGS for RPGR ORF15, only for cases of high clinical suspicion | SNVs, small indels | 57.1 (204 /357) |  |

Table S1: Compilation of diagnostic rates seen in other studies of inherited retinal disorders focussed on NGS based molecular diagnosis.

STGD Stargardt disease

CORD cone-rod dystrophy

RP retinitis pigmentosa

CSNB congenital stationary night blindness

FEVR Familial Exudative Vitreoretinopathy

MD Macular degeneration

LCA Leber congenital amaurosis

CHM Choroideremia

SV structural variants

SNV single nucleotide variant

EOSRD Early-Onset Severe Retinal Degeneration

US Usher syndrome

BCD Bietti crystalline dystrophy

CSNB congenital stationary night blindness

NA phenotype information for the ES cases was not separately available

n number of cases which underwent testing
