# Supplementary material for: Clinical exome sequencing for inherited retinal degenerations at a tertiary care center
Source: Sci Rep. 2022 Jun 7;12:9358. doi: 10.1038/s41598-022-13026-2 (PMC9174483; doi:10.1038/s41598-022-13026-2)
Supplement: Supplementary file 3 — Supplementary Information 3. [file 41598_2022_13026_MOESM3_ESM.docx]

**Supplemental table 2:** Variants and case details in the entire cohort of 357 cases

| **Case#** | **Sex** | **Age** | **ES samples** | **Result** | **OMIM Disease Association** | **Zygosity** | **Variant** | **1' phenotype category on imaging** | **2' phenotype category on imaging** | **ffERG** | **Phenotype classification** | **Case Category** |
| --- | --- | --- | --- | --- | --- | --- | --- | --- | --- | --- | --- | --- |
| 1 | M | 36 | TRIO | Negative |  |  |  | N.D. | N.D. | N.D. | Other | Negative |
| 2 | F | 37 | TRIO | Negative |  |  |  | N.D. | N.D. | N.D. | Rod-cone dystrophy | Negative |
| 3 | M | 87 | proband, daughters (2) | Pathogenic (homozygous) | AR Retinitis pigmentosa 25 (MIM:602772) | homozygous | EYS;Chr6:[64430632delCGATATTTAC];[64430632delCGATATTTAC]; NM_001142800.2:c.[9286_9295delGTAAATATCG];[9286_9295delGTAAATATCG]; NP_001136272.1:p.[(Val3096LeufsTer28)];[(Val3096LeufsTer28)] | Rod-cone dystrophy | RP | Rod-cone | Rod-cone dystrophy | Molecular Diagnosis |
| 4 | F | 45 | proband | Likely Pathogenic | AD Choroidal dystrophy, central areolar 2(MIM:613105); AR, AD Leber congenital amaurosis 18 (MIM:608133); AD Macular dystrophy, patterned, 1(MIM 169150); AD Macular dystrophy, vitelliform, 3 (MIM:608161); AR, AD Retinitis pigmentosa 7 and digenic form (MIM 608133); AR, AD Retinitis punctata albescens (MIM 136880) | heterozygous | PRPH2; Chr6:[42666160C>T];[=]; NM_000322.4:c.[914G>A];[=]; NP_000313.2:p.[(Gly305Asp)];[=] | Maculopathy | Adult Vitelliform Macular Dystrophy | N.D. | Macular dystrophy | Molecular Diagnosis |
| 5 | F | 10 | proband, mother, sister | Negative |  |  |  | N.D. | N.D. | N.D. | Cone-rod dystrophy | Negative |
| 6 | M | 57 | proband, daughter, son, sister | Likely Pathogenic (homozygous) | AR Retinitis pigmentosa 69 (MIM 615780) | homozygous | KIZ/PLK1S1;Chr20:[21117104C>T];[21117104C>T];NM_018474.4:c.[226C>T];[226C>T];NP_060944.3:p.[(Arg76Ter)];[(Arg76Ter)] | Rod-cone dystrophy | RP | Rod-cone | Rod-cone dystrophy | Molecular Diagnosis |
| 7^ | M | 59 | proband, sister, son | Likely Pathogenic | Cone-rod dystrophy, X-linked, 1 (MIM 304020); Macular degeneration, X-linked atrophic(MIM 300834); Retinitis pigmentosa 3 (MIM 300029); Retinitis pigmentosa, X-linked, and sinorespiratory infections, with or without deafness (MIM 300455) | hemizygous | RPGR; ChrX:[38145073delTC];[0]; NM_001034853.1:c.[3178_3179delGA];[0]; NP_001030025.1:p.[(Glu1060ArgfsTer18)];[0] | Maculopathy | Bull's eye macular dystrophy | ffERG normal | Macular dystrophy | Molecular Diagnosis |
| 8 | M | 11 | proband, father | Pathogenic (homozygous) | AR Retinitis pigmentosa 43 (MIM 613810) | homozygous | PDE6A; chr5:[149263009delT];[149263009delT];NM_000440.3:c.[2118delA;[2118delA]; NP_000431.2:p.[(Arg707GlyfsTer8)];[(Arg707GlyfsTer8)] | Rod-cone dystrophy | RP (severe) | Rod-cone | Rod-cone dystrophy | Molecular Diagnosis |
| 9 | F | 22 | proband, C1 (mother), C3 (sister) | 1 Likely Pathogenic and 1 VOUS identified, phase unknown | AR Joubert syndrome 5 (MIM 610188); Leber congenital amaurosis 10(MIM 611755); Meckel AR syndrome 4 (MIM 611134); AR Senior-Loken syndrome 6(MIM 610189) | two heterozygous variants, phase unknown | CEP290; Chr12:[88524330T>A](;)[88535011G>A]; NM_025114.3:c.[74C>T](;)[508A>T]; NP_079390.3:p.[(Ala25Val)](;)[(Lys170Ter)] | Rod-cone dystrophy | RP (severe) | Rod-cone | Rod-cone dystrophy | Likely Molecular Diagnosis |
| 10 | F | 15 | proband, mother | Likely Pathogenic | AD Retinitis pigmentosa 11(MIM 600138) | heterozygous | PRPF31; Chr19:[54629901A>G];[=]; NM_015629.3:c.[856-2A>G];[=] | Rod-cone dystrophy | RP | no ffERG | Rod-cone dystrophy | Molecular Diagnosis |
| 11 | M | 60 | proband, mother | VOUS | AD Occult macular dystrophy (MIM:613587) | heterozygous | RP1L1; Chr 8:[10480579C>T];[10480579C>T]; NM_178857.5:c.[133C>T];[=]; NP_849188.4:p.[(Arg45Trp)];[=] | Maculopathy | Optical gap/Occult maculopathy | Maculopathy | Macular dystrophy | Possible Molecular Diagnosis |
| 12 | M | 58 | proband, C3 (daughter, unaffected), C4 (sister) | 2 Likely Pathogenic compound heterozygous variants | AR Retinitis pigmentosa 25 (MIM:602772) | compound heterozygous | EYS; Chr6:[66044994C>A];[65596589C>T];NM_001142800.1:c.[1645G>T];[2992+1G>A]; NP_001136272.1:p.[(Glu549Ter)];[?] | Rod-cone dystrophy | RP | no ffERG | Rod-cone dystrophy | Molecular Diagnosis |
| 13 | F | 44 | proband | VOUS | AD Endosteal hyperostosis(MIM:144750); AD Osteopetrosis 1 (MIM:607634); AD Osteosclerosis, (MIM:144750); AD van Buchem disease type 2(OMIM 607636); AD Osteopetrosis, (OMIM 166710); AR Osteoporosis-pseudoglioma syndrome (OMIM 259770); AD/AR Exudative vitreoretinopathy 4(OMIM 601813) | Heterozygous | LRP5; Chr11:[68125147C>T];[68125147C>T]; NM_002335.3:c.[518C>T];[=]; NP_002326.2:p.[(Thr173Met)];[=] | N.D. | N.D. | no ffERG | Other | Possible Molecular Diagnosis |
| 14 | F | 39 | proband | 2 Likely pathogenic variants, phase unknown | AR Retinitis pigmentosa 39 (MIM 613809); AR Usher syndrome, type 2A (MIM 276901) | 2 heterozygous variants, phase unknown | USH2A; Chr1:[215963510C>T](;)[216498866_216498867insTGGC];NM_206933.2:c.[10073G>A](;)[920_923dupGCCA];NP_996816.2:p.[(Cys3358Tyr)](;)[(His308Glnfs)] | Rod-cone dystrophy | RP (mild) | Rod-cone | Rod-cone dystrophy | Likely Molecular Diagnosis |
| 15 | M | 66 | proband, C3 (daughter), C4 (sister) | 2 Likely Pathogenic Variants in cis in AR disease gene | AR Retinitis pigmentosa 25 (MIM:602772) | 1 variant in an AR disease (2 Likely Pathogenic Variants in cis) | EYS; Chr6:[66112400A>T;64431272_64431279delTCTGCATG], NM_001142800:c.[1155T>A;8648_8655delCATGC];[=]; NP_001136272:p.[(Cys385Ter);(Thr2883LysfsTer4)];[=] | Rod-cone dystrophy | RP | Rod-cone | Rod-cone dystrophy | Other |
| 16 | M | 41 | proband | 2 Likely pathogenic variants, phase unknown | AR Nephronophthisis 4 (MIM 606966); AR Senior-Loken syndrome 4 (MIM 606996) | 2 heterozygous variants, phase unknown | NPHP4; Chr1:[5969253G>A](;)[5993384-5993385insGG], NM_015102.4:c.[1124_1125insCC](;)[1463C>T]; NP_055917.1:p.[(Ser376LeufsTer31)](;)[(Arg488Ter)] | Cone-rod dystrophy | RP | Cone more attenuated than rods | Cone-rod dystrophy | Likely Molecular Diagnosis |
| 17 | M | 56 | proband | Likely pathogenic (Homozygous) | AR Retinitis pigmentosa 39 (MIM 613809); AR Usher syndrome, type 2A (MIM 276901) | homozygous | USH2A; chr1:[215963510C>T];[215963510C>T];NM_206933.2:c.[10073G>A];10073G>A]; NP_996816.2:p.[(Cys3358Tyr)];[(Cys3358Tyr)] | Rod-cone dystrophy | RP | Rod-cone | Rod-cone dystrophy | Molecular Diagnosis |
| 18 | M | 62 | proband | Pathogenic | AD Retinitis pigmentosa 13 (MIM 600059) | heterozygous | PRPF8; Chr17:[1554113delC];[=]; NM_ 006445.3:c.[6991delG];[=]; NP_006436.3:p.[(Glu2331fs)];[=] | Rod-cone dystrophy | RP (mild) | Rod-cone | Rod-cone dystrophy | Molecular Diagnosis |
| 19 | M | 69 | proband | Negative |  |  |  | Rod-cone dystrophy | RP | no ffERG | Rod-cone dystrophy | Negative |
| 20 | M | 45 | proband | Negative |  |  |  | Cone-rod dystrophy | RP (mild, sectoral) | Cone more attenuated than rods | Cone-rod dystrophy | Negative |
| 21 | F | 36 | proband | VOUS | AD Occult macular dystrophy (MIM 613587) | Heterozygous | RP1L1; Chr8:[10480579G>A];[=]; NM_178857.5:c.[133C>T];[=]; NP_849188.4:p.[(Arg45Trp)];[=] | Maculopathy | Optical gap/Occult maculopathy | no ffERG | Macular dystrophy | Possible Molecular Diagnosis |
| 22 | M | 49 | proband, C3 (daughter, buccal) | VOUS | AD Occult macular dystrophy (MIM 613587) | Heterozygous | RP1L1;Chr8:[10480143C>T];[=];NM_178857.5:c.[569G>A];[=];NP_849188.4:p.[(Arg190His)];[=] | Maculopathy | Pattern dystrophy | Pattern macular dystrophy | Macular dystrophy | Possible Molecular Diagnosis |
| 23 | F | 24 | proband, mother | Pathogenic (Homozygous) | AR Retinitis pigmentosa 26 (MIM 608380) | Homozygous | CERKL;Chr2:[182423344G>A];[182423344G>A]; NM_001030311.2:c.[847C>T];[847C>T];NP_001025482:p.[(Arg283Ter)];[(Arg283Ter)] | Rod-cone dystrophy | CERKL gene-associated RP | Rod-cone | Rod-cone dystrophy | Molecular Diagnosis |
| 24 | F | 7 | proband | Negative |  |  |  | Cone dystrophy | N.D. | cone attenuated, rods normal | Cone dystrophy | Negative |
| 25 | M | 60 | proband, C1 (mother), C3 (sister), C4 (daughter) | VOUS | AD/AR, Retinitis pigmentosa 44 (MIM 613769) | Heterozygous | RGR; Chr10:[86008695C>A];[=];NM_002921.3:c.[266C>A];[=]; NP_002912.2:p.[(Ser89Ter)];[=] | Cone-rod dystrophy | Geographic atrophy (diffuse-trickling subtype) | Cone more attenuated than rods | Cone-rod dystrophy | Possible Molecular Diagnosis |
| 26 | F | 59 | proband | CNGB1-2 Likely Pathogenic Variants of Unknown Phase; KCNV2*-* 2 Variants of Uncertain Significance of Unknown Phase (?cis); GALC -1 Variant of Uncertain Significance and Likely Pathogenic Variant of Unknown Phase | CNGB1 - AR Retinitis pigmentosa 45 (MIM 613767); KCNV2-AR Retinal cone dystrophy 3B (MIM 610356); GALC-AR Krabbe disease (MIM:245200) | CNGB1-Two heterozygous variants, phase unknown; KCNV2- 2 heterozygous variants, phase unknown (?cis); GALC -2 heterozygous variants, phase unknown | GALC; Chr14:[88452887C>A](;)[88454857C>T]; NM_000153.3:c.[206G>A](;)[338G>T]; NP_000144.2:p.[(Arg69Gln)](;)[(Glu130Ter)]; CNGB1; Chr16:[57931392ACCC>ACC](;)[57953064G>T]; NM_001297.4:c.[1896C>A](;)[3150delG]; NP_001288.3:p.[(Cys632Ter)](:)[(Gly1050GlyfsTer13)] KCNV2; Chr9:[2718159C>A](;)[2718517A>T]; NM_133497.3:c.[420C>A](;)[778A>T]; NP_598004.1:p.[(Asp140Glu)](;)[(Lys260Ter)] | Rod-cone dystrophy | RP (severe) | Rods/Cones non-recordable on ffERG | Rod-cone dystrophy | Likely Molecular Diagnosis |
| 27 | F | 38 | proband | Negative |  |  |  | Rod-cone dystrophy | RP (severe) | Rod-cone | Rod-cone dystrophy | Negative |
| 28 | F | 58 | proband | 2 Pathogenic variants, phase unknown | AR Retinitis pigmentosa 54 (MIM 613428) | 2heterozygous variants Identified, Phase Unknown | PCARE; Chr 2:[29295145](;)[29295322]; NM_001029883.2:c.[1979_1982delGCAA](:)[1804_1805delAG]; NP_001025054.1:p.[(Ser660Thrfs*84)](:)[(His603Argfs*76)] | Cone-rod dystrophy | N.D. | no ffERG | Cone-rod dystrophy | Likely Molecular Diagnosis |
| 29* | F | 42 | TRIO | VOUS in PRPF3; 2 VOUS in CRB1, in trans | PRPF3 - AD Retinitis pigmentosa 18 (MIM 601414); CRB1-AR Leber congenital amaurosis 8 (MIM 613835); AD Pigmented paravenous chorioretinal atrophy (MIM 172870); AR Retinitis pigmentosa-12, autosomal recessive (MIM 600105) | PRPF3- heterozygous variant; CRB1-two variants in trans | PRPF3; Chr1:[150305629G>C];[=]; NM_004698.2:c.[687C>G];[=]; NP_004689.1:p.[(Met229Ile)];[=],  CRB1; Chr1:[197391063A>G];[197404007A>T]; NM_201253.2:c.[2105T>C];[3014T>A]; NP_957705.1:p.[(Tyr702Cys)];[(Asp1005Val)] | Cone-rod dystrophy | N.D. | Cones non-recordable, rods attenuated | Cone-rod dystrophy | Possible Molecular Diagnosis |
| 30 | F | 36 | TRIO | Negative |  |  |  | Maculopathy | Familial | normal | Macular dystrophy | Negative |
| 31 | M | 51 | proband | VOUS | AD Macular dystrophy, vitelliform 4 (MIM:616151) | Heterozygous | IMPG1; Chr6:[76640728G>A];[=]; NM_001563.3:c.[2185G>A];[=]; NP_001554.2:p.[(Leu729Phe)];[=] | Cone-rod dystrophy | N.D. | Cone more attenuated than rods | Cone-rod dystrophy | Possible Molecular Diagnosis |
| 32 | M | 30 | proband | Negative |  |  |  | Rod-cone dystrophy | RP | Rod-cone | Rod-cone dystrophy | Negative |
| 33 | M | 66 | proband | Negative |  |  |  | Cone-rod dystrophy | N.D. | Cone more attenuated than rods | Cone-rod dystrophy | Negative |
| 34 | F | 58 | proband | Two Pathogenic Variants, Phase Unknown | AR Retinitis pigmentosa-40 (MIM 613801); AD Night blindness, congenital stationary 2 (MIM 163500) | Two heterozygous Variants, Phase Unknown | PDE6B; Chr4:[654274delC](;)[655977C>T]; NM_000283.3:c.[1488delC](;)[1669C>T]; NP_000274.2:p.[(Thr497Profs*78)](;)[(His557Tyr)] | Rod-cone dystrophy | RP | Rod-cone | Rod-cone dystrophy | Likely Molecular Diagnosis |
| 35 | F | 61 | proband | Two likely pathogenic variants, Phase Unknown | AR Retinitis pigmentosa 25 (MIM:602772) | Two heterozygous variants, Phase Unknown | EYS; Chr6:[64776241delA](;)[64430624delGAGT];NM_001142800.1:c.[6714delT](;)[9299_9302delCTCA]; NP_001136272.1:p.[(Ile2239Serfs*17)](;)[(Thr3100Lysfs*26)] | Rod-cone dystrophy | RP (severe) | Rods/Cones non recordable on ffERG | Rod-cone dystrophy | Likely Molecular Diagnosis |
| 36 | M | 8 | TRIO | VOUS (homozygous) | AR achromatopsia 3 (MIM:262300) | homozygous | CNGB3; Chr8:[87644976T>C];[87644976T>C]; NM_019098.4:c.[1320+4A>G];[1320+4A>G] | Cone dystrophy | Achromatopsia | rods normal, cones non recordable on ffERG | Cone dystrophy | Possible Molecular Diagnosis |
| 37 | F | 49 | proband | Negative |  |  |  | Rod-cone dystrophy | RP | N.D. | Rod-cone dystrophy | Negative |
| 38 | F | 42 | proband | Negative |  |  |  | Rod-cone dystrophy | RP | N.D. | Rod-cone dystrophy | Negative |
| 39 | M | 81 | proband | Two Likely Pathogenic variants, phase unknown | AR Retinitis pigmentosa 39 (MIM 613809); AR Usher syndrome, type 2A (MIM 276901) | Two heterozygous variants, phase unknown | USH2A; Chr1:[215848678C>T](:)[216062127delT]; NM_206933.2:c.[7863delA](;)[12575G>A]; NP_996816.2p.[(2623Hisfs*18)](;)[(Arg4192His)] | Rod-cone dystrophy | RP | N.D. | Rod-cone dystrophy | Likely Molecular Diagnosis |
| 40 | M | 22 | proband | Likely Pathogenic, VOUS | Cone-rod dystrophy, X-linked, 1 (MIM 304020); Macular degeneration, X-linked atrophic(MIM 300834); Retinitis pigmentosa 3 (MIM 300029); Retinitis pigmentosa, X-linked, and sinorespiratory infections, with or without deafness (MIM 300455); AD/AR Cone-rod dystrophy 12 (MIM:612657), AD Macular dystrophy, retinal, 2 (MIM:608051), AR Retinitis Pigmentosa 41 (MIM:612095), AD Stargardt disease 4 (MIM:603786) | Hemizygous, Heterozygous | RPGR; ChrX:[38145836CC>AA];[0]; NM_001034853.1: c.[2415_2416delGGinsTT];[0]; NP_001030025.1: p.[(Glu806Ter)];[0], PROM1; Chr4:[16040622T>C];[=];NM_006017.2:c.[223A>G];[=]; NP_006008.1:p.[(Thr75Ala)];[=] | Rod-cone dystrophy | RP | N.D. | Rod-cone dystrophy | Molecular Diagnosis |
| 41 | F | 45 | proband | Two Likely Pathogenic Variants identified, phase unknown | AR Retinitis pigmentosa 25 (MIM:602772) | Two heterozygous variants, phase unknown | EYS; Chr6:[66115144_66115160delGGCTGGAAGATCCTTTT](:)[64709008delG]; NM_001142800.1:c.[963_979delAAAAGGATCTTCCAGCC](;)[6794delC]; NP_001136272.1:p.[(Gly323TrpfsTer2)](;)[(Pro2265GlnfsTer46)] | Rod-cone dystrophy | RP (severe) | N.D. | Rod-cone dystrophy | Likely Molecular Diagnosis |
| 42^ | M | 69 | proband | Pathogenic Variant | Cone-rod dystrophy, X-linked, 1 (MIM 304020); Macular degeneration, X-linked atrophic(MIM 300834); Retinitis pigmentosa 3 (MIM 300029); Retinitis pigmentosa, X-linked, and sinorespiratory infections, with or without deafness (MIM 300455) | Hemizygous | RPGR;ChrX:[38145073_38145074delTC];NM_001034853.1:c.[3178_3179delGA];[0]; NP_001030025.1:p.[(Glu1060ArgfsTer18)];[0] | Cone-rod dystrophy | Bull's eye macular dystrophy | cone attenuated, rods normal | Cone-rod dystrophy | Molecular Diagnosis |
| 43 | F | 38 | proband | VOUS | GPR179 - AR Night blindness, congenital stationary (complete), 1E, (MIM 614565); SEMA4A- AR Cone-rod dystrophy 10 (MIM 610283); AR, AD Retinitis pigmentosa 35(MIM 610282) | GPR179-2 heterozygous variants phase unknown; SEMA4-2 heterozygous variants, phase unknown | GPR179; Chr17:[36484461C>T](:)[36485527T>A];NM_001004334.3:c.[4991G>A](;)[3925A>T]; NP_001004334.3:p.[(Arg1664His)](;)[(Lys1309Ter)], SEMA4A; Chr1:[156124389G>A](;)[156145436G>A]; NM_022367.3:c.[20G>A](;)[1682G>A]; NP_001180230.1:p.[(Gly7Asp)](;)[Arg561His] | Maculopathy | Bull's eye macular dystrophy | no ffERG | Macular dystrophy | Possible Molecular Diagnosis |
| 44 | F | 49 | proband | Negative |  |  |  | Drug-induced retinal toxicity | N.D. | N.D. | Other | Negative |
| 45 | F | 42 | proband | Likely Pathogenic | AD Choroidal dystrophy, central areolar 2(MIM:613105); AR, AD Leber congenital amaurosis 18 (MIM:608133); AD Macular dystrophy, patterned, 1(MIM 169150); AD Macular dystrophy, vitelliform, 3 (MIM:608161); AR, AD Retinitis pigmentosa 7 and digenic form (MIM 608133); AR, AD Retinitis punctata albescens (MIM 136880) | heterozygous | PRPH2; Chr6:[42666160C>T];[=]; NM_000322.4:c.[914G>A];[=]; NP_000313.2:p.[(Gly305Asp)];[=] | Maculopathy | Adult Vitelliform Macular Dystrophy | no ffERG | Macular dystrophy | Molecular Diagnosis |
| 46 | M | 70 | proband | 2 Likely pathogenic variants, phase unknown | AR Retinitis pigmentosa 25 (MIM:602772) | 2 heterozygous variants, phase unknown | EYS; Chr6:[65098733delC](;)[66063502G>T]; NM_001142800.1:c.[1308C>A](;)[5928delG]; NP_001136272.1:p.[(Cys436Ter)](;)[(Arg1976SerfsTer11)] | Rod-cone dystrophy | RP (severe) | no ffERG | Rod-cone dystrophy | Likely Molecular Diagnosis |
| 47^ | F | 75 | proband | Pathogenic | Cone-rod dystrophy, X-linked, 1 (MIM 304020); Macular degeneration, X-linked atrophic(MIM 300834); Retinitis pigmentosa 3 (MIM 300029); Retinitis pigmentosa, X-linked, and sinorespiratory infections, with or without deafness (MIM 300455) | heterozygous | RPGR; Chr X:[38146015delTC];[=]; NM_001034853.1:c.[2236_2237delGA];[=]; NP_000319.1:p.[(Glu746Argfs*23)];[=] | Rod-cone dystrophy | RP (severe) | no ffERG | Rod-cone dystrophy | Molecular Diagnosis |
| 48 | F | 28 | TRIO | Pathogenic | AR Cone-rod dystrophy 3(MIM:604116); AR Fundus flavimaculatus (MIM:248200); AR Retinal dystrophy, early-onset severe (MIM:248200); AR Retinitis pigmentosa 19 (MIM: 601718); AR Stargardt disease 1 (MIM:248200) | Compound heterozygous | ABCA4; Chr1:[94495071C>T];[ 94473807C>T]; NM_000350.2:c.[4469G>A];[5882G>A]; NP_000341.2:p.[(Cys1490Tyr)];[(Gly1961Glu)] | Maculopathy | STGD | ffERG normal | Macular dystrophy | Molecular Diagnosis |
| 49 | M | 17 | proband | Pathogenic | Cone-rod dystrophy, X-linked, 1 (MIM 304020); Macular degeneration, X-linked atrophic(MIM 300834); Retinitis pigmentosa 3 (MIM 300029); Retinitis pigmentosa, X-linked, and sinorespiratory infections, with or without deafness (MIM 300455) | hemizygous | RPGR; ChrX:[38160498_38160500delACC];[0], NM_001034853.1:c.[1059_1059+2delGGT];[=] | Rod-cone dystrophy | RP (mild) | N.D. | Rod-cone dystrophy | Molecular Diagnosis |
| 50 | M | 85 | proband | VOUS in a AD disease gene | Autosomal dominant cone-rod dystrophy 5 [MIM:600977] | heterozygous | PITPNM3,Chr17:[6360954A>G];[=], NM_031220.3:c.[2579T>C];[=], NP_112497.2:p.[(Ile860Thr)];[(=)] | Cone-rod dystrophy? | N.D. | Rod-cone | Rod-cone dystrophy | Possible Molecular Diagnosis |
| 51^#1^ | M | 57 | proband | Homozygous Pathogenic variants in AR disease | AR Retinitis pigmentosa 69 (MIM 615780) | Homozygous | KIZ Chr20:[21112767_21112770delAACT];[21112767_21112770delAACT], NM_018474.4:c.[119_122delAACT];[119_122delAACT], NP_060944.3:p.[(Lys40IlefsTer14)];[(Lys40IlefsTer14)] | Rod-cone dystrophy | RP | N.D. | Rod-cone dystrophy | Molecular Diagnosis |
| 52 | F | 45 | proband | Homozygous Pathogenic variants in AR disease | AR Retinitis pigmentosa 26 (MIM 608380) | homozygous | CERKL;Chr2:[182423344G>A];[182423344G>A]; NM_201548.4:c.[769C>T];[769C>T] NP_963842.1:p.[(Arg257Ter)];[(Arg257Ter)] | Rod-cone dystrophy | CERKL gene-associated RP | N.D. | Rod-cone dystrophy | Molecular Diagnosis |
| 53 | M | 40 | proband | Likely Pathogenic and VOUS in a AR disease gene phase unknown ; VOUS in a gene with AD disease | USH2A:AR Retinitis pigmentosa 39 (MIM 613809); AR Usher syndrome, type 2A (MIM 276901);PROM1: AD/AR Cone-rod dystrophy 12 (MIM:612657), AD Macular dystrophy, retinal, 2 (MIM:608051), AR Retinitis Pigmentosa 41 (MIM:612095), AD Stargardt disease 4 (MIM:603786) | USH2A:Two heterozygous Variants, Phase Unknown; PROM1: one heterozygous variant | USH2A; Chr1:[216017635C>T](;)[216019213A>G]; NM_206933.2:c.[9008T>C](;)[9258+1G>A]; NP_996816.2:p.[(Val3003Ala)];[?],  PROM1;Chr4:[16025960C>A];[=]; NM_006017.2:c.[652C>T];[=] NP_006008.1:p.[(Gln218Ter)];(=] | Rod-cone dystrophy | RP | N.D. | Rod-cone dystrophy | Likely Molecular Diagnosis |
| 54 | M | 26 | proband | Heterozygous Pathogenic variant in AD disease | AD Choroidal dystrophy, central areolar 2(MIM:613105); AR, AD Leber congenital amaurosis 18 (MIM:608133); AD Macular dystrophy, patterned, 1(MIM 169150); AD Macular dystrophy, vitelliform, 3 (MIM:608161); AR, AD Retinitis pigmentosa 7 and digenic form (MIM 608133); AR, AD Retinitis punctata albescens (MIM 136880) | heterozygous | PRPH2;Chr6:[42689559G>A];[=], NM_000322.4:c.[514C>T];[=]; NP_000313.2:p.[(Arg172Trp)];[=] | Maculopathy | Pattern dystrophy | ffERG normal | Macular dystrophy | Molecular Diagnosis |
| 55 | F | 51 | proband | Negative |  |  |  | Maculopathy vs AMD | Familial drusen | N.D. | Macular dystrophy | Negative |
| 56 | M | 33 | proband | Negative |  |  |  | Rod-cone dystrophy | RP | N.D. | Rod-cone dystrophy | Negative |
| 57^#1^ | M | 33 | proband | 1 Pathogenic Variant in an AR disease | AD, AR, Fundus Albipunctatus (OMIM: 136880); AR Bothnia retinal dystrophy (OMIM: 607475);Newfoundland rod-cone dystrophy (OMIM: 607476);AD,AR,Retinitis punctate albescens(OMIM:136880) | Heterozygous | RLBP1;Chr15:[89754981A>T];[=];NM_000326.4:c.[677T>A];[ =], NP_000317.1:p.[(Met226Lys)};[=] | Rod-cone dystrophy | RP (mild, sectoral) | ffERG normal | Rod-cone dystrophy | Other |
| 58 | F | 52 | proband | one Likely Pathogenic and one VOUS in a AR disease gene, phase unknown | AR Retinitis pigmentosa 39 (MIM 613809); AR Usher syndrome, type 2A (MIM 276901) | 2 heterozygous variants, phase unknown | USH2A; Chr1:[216498895delG](;)[216246240T>C]; NM_206933.2:c.[895delC](;)[5848A>G]; NP_996816.2:p.[(Thr1950Ala)](;)[(Gln299AsnfsTer37)] | Rod-cone dystrophy | RP | N.D. | Rod-cone dystrophy | Likely Molecular Diagnosis |
| 59 | F | 62 | proband | Negative |  |  |  | N.D. | N.D. | Doesn't look like RP, severe maculopathy/STGD1-like | Rod-cone dystrophy | Negative |
| 60 | M | 73 | proband | Negative |  |  |  | Rod-cone dystrophy | RP | N.D. | Rod-cone dystrophy | Negative |
| 61 | F | 11 | Trio | Homozygous Pathogenic variants in AR disease | AR Cone-rod dystrophy 13 (MIM 608194); AR Leber congenital amaurosis 6 (MIM 613826) | homozygous | RPGRIP1; Chr14:[21769345delACAG];[21769345delACAG]; NM_020366.3:c.[440_443delACAG];[440_443delACAG], NP_065099.3p.[(Arg148Serfs*21)];[(Arg148Serfs*21)] | Rod-cone dystrophy | RP | N.D. | Rod-cone dystrophy | Molecular Diagnosis |
| 62 | F | 57 | proband | Negative |  |  |  | Rod-cone dystrophy | RP | N.D. | Rod-cone dystrophy | Negative |
| 63 | F | 42 | proband | heterozygous VOUS in a AD disease gene | AD Night blindness, congenital stationary, 3(OMIM 610444); AR Night blindness, congenital stationary, type 1G (MIM 616389) | heterozygous | GNAT1;Chr3:[50231038G>A];[=]; NM_144499.2:c.[391G>A];[=]; NP_653082.1:p.[(Gly131Ser)];[=] | Rod-cone dystrophy | RP | N.D. | Rod-cone dystrophy | Possible Molecular Diagnosis |
| 64 | M | 70 | proband | Hemizygous Likely Pathogenic | XLR Retinoschisis (MIM 312700) | hemizygous | RS1;ChrX:[18662655delT;[0], NM_000330.3:c.[416delA];[0],NP_000321.1:p.[(Gln139Argfs*10)];[0] | Maculopathy | Retinoschisis | electronegative ffERG | Macular dystrophy | Molecular Diagnosis |
| 65 | M | 30 | proband | Negative |  |  |  | Rod-cone dystrophy | N.D. | N.D. | Rod-cone dystrophy | Negative |
| 66 | M | 73 | proband | Homozygous Pathogenic variants in AR disease | AR Enhanced S-cone syndrome (MIM 268100); AR, AD Retinitis pigmentosa 37 (MIM 611131) | homozygous | NR2E3;Chr15:[72105913G>A];[72105913G>A], NM_014249.3:c.[932G>A];[932G>A] NP_055064.1:p.[(Arg311Gln)];[(Arg311Gln)] | Rod-cone dystrophy | RP | classic ESCS phenotype | Rod-cone dystrophy | Molecular Diagnosis |
| 67 | F | 48 | proband, mother | Negative |  |  |  | N.D. | N.D. | N.D. | Macular dystrophy | Negative |
| 68 | F | 62 | proband | Likely pathogenic | AD Night blindness, congenital stationary 1(MIM 610445); AR, AD Retinitis pigmentosa 4 (MIM 613731); AR, AD Retinitis punctata albescens (MIM 136880) | heterozygous | RHO, Chr3:[129251119T>C];[=]; NM_000539.3:c.[556T>C];[=]; NP_000530.1:p.[(Ser186Pro)];[=] | Rod-cone dystrophy | RP | N.D. | Rod-cone dystrophy | Molecular Diagnosis |
| 69 | M | 57 | proband | 1 Likely pathogenic Variant in an AR disease gene | AR Retinitis pigmentosa-40 (MIM 613801); AD Night blindness, congenital stationary 2 (MIM 163500) | heterozygous | PDE6B; Chr4:[655963G>A];[=]; NM_000283.3:c.[1655G>A];[=]; NP_000274.2:p.[(Arg552Gln)];[=] | Rod-cone dystrophy | RP | N.D. | Rod-cone dystrophy | Other |
| 70 | M | 77 | proband | 2 compound heterozygous VOUS in AR disease gene | AR Leber congenital amaurosis 8 (MIM 613835); AD Pigmented paravenous chorioretinal atrophy (MIM 172870); AR Retinitis pigmentosa-12 (MIM 600105) | Compound heterozygous | CRB1;Chr1:[197390648G>T];[197390666G>A]; NM_201253.2:c.[1690G>T];[1708G>A]; NP_957705.1:p.[(Asp564Tyr)];[(Val570Met)] | Rod-cone dystrophy | RP | N.D. | Rod-cone dystrophy | Likely Molecular Diagnosis |
| 71 | M | 47 | proband | VOUS | Autosomal dominant cone-rod dystrophy 5 [MIM:600977] | heterozygous | PITPNM3; Chr17:[6371570C>T];[=]; NM_031220.3:c.[1865G>A];[=]; NP_112497.2:p.[(Arg622His)];[=] | Rod-cone dystrophy | RP | mild RP | Rod-cone dystrophy | Possible Molecular Diagnosis |
| 72 | M | 56 | proband | one Pathogenic and one VOUS in a AR disease gene, phase unknown | AR Retinitis pigmentosa 39 (MIM 613809); AR Usher syndrome, type 2A (MIM 276901) | 2 heterozygous variants, phase unknown | USH2A; Chr1:[216420436delC](;)[215848679G>A]; NM_206933.2:c.[2299delG](:)[12574C>T]; NP_996816.2:p.[(Glu767Serfs)](;)[(Arg4192Cys)] | Rod-cone dystrophy | RP | N.D. | Rod-cone dystrophy | Likely Molecular Diagnosis |
| 73 | F | 54 | proband | Likely Pathogenic | AD Night blindness, congenital stationary 1(MIM 610445); AR, AD Retinitis pigmentosa 4 (MIM 613731); AR, AD Retinitis punctata albescens (MIM 136880) | Heterozygous | RHO;Chr3:[129252539C>T];[=]; NM_000539.3:c.[1025G>A];[=]; NP_000530.1:p.[(Thr342Met)];[=] | Rod-cone dystrophy | RP | N.D. | Rod-cone dystrophy | Molecular Diagnosis |
| 74 | M | 59 | proband | Single VOUS in AR disease | AR Retinitis pigmentosa 61 (MIM 614180); AR Usher syndrome, type 3A (MIM 276902) | Heterozygous | CLRN1;Chr3:[150690352A>C];[=]; NM_174878.2:c.[144T>G];[=]; NP_777367.1:p.[(Asn48Lys)];[=] | Rod-cone dystrophy | US | N.D. | Rod-cone dystrophy | Other |
| 75 | F | 38 | proband | VOUS | AD Night blindness, congenital stationary 1(MIM 610445); AR, AD Retinitis pigmentosa 4 (MIM 613731); AR, AD Retinitis punctata albescens (MIM 136880) | Heterozygous | RHO; Chr3:[129251104 G>A];[=]; NM_000539.3:c.[241G>A];[=];NP_000530.1:p.[(Glu181Lys)];[=] | Cone-rod dystrophy | N.D. | N.D. | Cone-rod dystrophy | Possible Molecular Diagnosis |
| 76 | M | 63 | proband | 1 Likely pathogenic and VOUS in ABCA4 gene phase unknown ; VOUS homozygous | ABCA4: AR Cone-rod dystrophy 3(MIM 604116); AR Fundus flavimaculatus(MIM 248200); AR Retinal dystrophy, early-onset severe(MIM 248200); AR Retinitis pigmentosa 19 (MIM 601718); AR Stargardt disease 1 (MIM 248200); PROM1:AD/AR Cone-rod dystrophy 12 (MIM:612657), AD Macular dystrophy, retinal, 2 (MIM:608051), AR Retinitis Pigmentosa 41 (MIM:612095), AD Stargardt disease 4 (MIM:603786) | ABCA4- heterozygous variants, phase unknown; PROM1:homozygous | ABCA4;Chr1:[94471055C>T](;)[94505667G>A](;)[94586601T>C]; NM_000350.2:c.[1A>G](;)[3758C>T](;)[6089G>A]; NP_000341.2:p.[?](;)[(Thr1253Met)](;)[(Arg2030Gln)]   PROM1; Chr4:[16019985C>A];[16019985C>A]; NM_006017.2:c.[963G>T];[963G>T]; NP_006008.1:p.[Leu321Phe];[Leu321Phe] | Maculopathy | STGD | N.D. | Macular dystrophy | Likely Molecular Diagnosis |
| 77 | M | 69 | proband | Likely pathogenic homozygous variants | AR Retinitis pigmentosa 45 (MIM 613767) | Homozygous | CNGB1; Chr16:[57949173G>A];[57949173G>A]; NM_001297.4:c.[2284C>T];[2284C>T]; NP_001288.3:p.[(Arg762Cys)];[(Arg762Cys)] | Rod-cone dystrophy | RP | N.D. | Rod-cone dystrophy | Molecular Diagnosis |
| 78 | M | 14 | proband | negative |  |  |  | Rod-cone dystrophy | RP | rods down on ffERG | Rod-cone dystrophy | Negative |
| 79 | F | 36 | proband | VOUS | AD Retinitis pigmentosa 42(612943); AR Cold-induced sweating syndrome 3(617055) | Heterozygous | KLHL7:Chr7:[23164782A>G];[=]; NM_001031710.2:c.[433A>G];[=];NP_001026880.2:p.[(Asn145Asp)];[=] | Rod-cone dystrophy | RP | N.D. | Rod-cone dystrophy | Possible Molecular Diagnosis |
| 80 | M | 37 | proband, mother | 1 Pathogenic variant in a AR disease | AR Cone-rod dystrophy 3(MIM 604116); AR Fundus flavimaculatus(MIM 248200); AR Retinal dystrophy, early-onset severe(MIM 248200); AR Retinitis pigmentosa 19 (MIM 601718); AR Stargardt disease 1 (MIM 248200) | Heterozygous | ABCA4: Chr1:[94508969G>A;94528806A>G];[=]; NM_000350.2:c.[3113C>T;1622T>C];[=]; NP_000341.2:p.[(Ala1038Val;Leu541Pro)];[(=)] | Maculopathy | Pattern dystrophy | N.D. | Macular dystrophy | Other |
| 81 | F | 45 | proband | Likely pathogenic and VOUS, phase unknown in a AR disease gene | AR Retinitis pigmentosa 62 (MIM 614181) | 2 heterozygous variants, phase unknown | MAK: Chr6:[10770433_10770436delTGAT](;)[10809140G>C]; NM_005906.5:c.[1625_1628delATCA](;)[394C>G]; NP_005897.1:p.[(Asn542SerfsTer22)](;)[(Leu132Val)] | Cone dystrophy | N.D. | N.D. | Cone dystrophy | Likely Molecular Diagnosis |
| 82 | M | 44 | proband | Pathogenic | AR, AD Cone-rod dystrophy 6(MIM 601777); AR Leber congenital amaurosis 1(MIM 204000) | Heterozygous | GUCY2D; Chr17:[7918018C>T]; NM_000180.3:c.[2512C>T];[=] NP_000171.1:p.[(Arg838Cys)];[(=]) | Cone-rod dystrophy | N.D. | N.D. | Cone-rod dystrophy | Molecular Diagnosis |
| 83 | M | 64 | proband, Affected Child (F), Sibling (M) | Pathogenic | AD Night blindness, congenital stationary 1(MIM 610445); AR, AD Retinitis pigmentosa 4 (MIM 613731); AR, AD Retinitis punctata albescens (MIM 136880) | Heterozygous | RHO; Chr3:[129251479C>T];[=]; NM_000539.3:c.[800C>T];[=] NP_000530.1:p.(Pro267Leu);[=] | Rod-cone dystrophy | RP | N.D. | Rod-cone dystrophy | Molecular Diagnosis |
| 84 | M | 37 | proband | Negative |  |  |  | Rod-cone dystrophy | RP | N.D. | Rod-cone dystrophy | Negative |
| 85 | F | 88 | proband | Negative |  |  |  | Cone-rod dystrophy | N.D. | N.D. | Cone-rod dystrophy | Negative |
| 86 | M | 60 | proband | VOUS | AD Cone-rod retinal dystrophy-2(MIM 120970); AD, AR Leber congenital amaurosis 7 (MIM 613829) | Heterozygous | CRX: Chr19:[48339518G>A];[=]; NM_000554.5:c.[119G>A];[=] NP_000545.1:p.[(Arg40Gln)];[=] | Cone-rod dystrophy | N.D. | N.D. | Cone-rod dystrophy | Possible Molecular Diagnosis |
| 87 | M | 57 | proband | 1 Pathogenic Variant in an AR disease | AR Bietti crystalline corneoretinal dystrophy (MIM 210370) | Heterozygous | CYP4V2:Chr4:[187122303_187122319delTCATACAGGTCATCGCTinsGC];[=]; NM_207352.3:c.802-8_810delGTCATCGCTinsGC;[=] | Rod-cone dystrophy | RP | N.D. | Rod-cone dystrophy | Other |
| 88 | F | 44 | proband | Negative |  |  |  | Rod-cone dystrophy | RP (severe) | N.D. | Rod-cone dystrophy | Negative |
| 89 | M | 53 | proband | Negative |  |  |  | Rod-cone dystrophy | RP (severe) | N.D. | Rod-cone dystrophy | Negative |
| 90 | M | 31 | proband | VOUS | AD Retinitis pigmentosa 10(MIM 180105); Leber congenital amaurosis 11(MIM 613837) | Heterozygous | IMPDH1:Chr7:[128038580G>A];[=]; NM_000883.3:c.[962C>T];[=]; NP_000874.2:p.[(Arg321Val)];[(=)] | Rod-cone dystrophy | RP (severe) | N.D. | Rod-cone dystrophy | Possible Molecular Diagnosis |
| 91 | F | 69 | proband | Negative |  |  |  | Cone-rod dystrophy | N.D. | Rod and cone attenuation | Cone-rod dystrophy | Negative |
| 92* | M | 65 | proband | Likely Pathogenic and VOUS in trans; 1 Likely Pathogenic Variant in an AR disease | AR Retinitis pigmentosa 39 (MIM 613809); AR Usher syndrome, type 2A (MIM 276901); ABCA4 -AR Cone-rod dystrophy 3(MIM 604116); AR Fundus flavimaculatus(MIM 248200); AR Retinal dystrophy, early-onset severe(MIM 248200); AR Retinitis pigmentosa 19 (MIM 601718); AR Stargardt disease 1 (MIM 248200) | USH2A-Compound heterozygous variants; ABCA4-Heterozygous | USH2A; Chr1:[215933187T>C];[216011330] NM_206933.2:c.[11048-2A>G];[9371+3A>G] ABCA4; Chr1:[94543389C>T];[=]; c.[1411G>A];[=] p.[(Glu471Lys)];[=]; | Rod-cone dystrophy | RP | N.D. | Rod-cone dystrophy | Possible Molecular Diagnosis |
| 93 | M | 33 | proband | ABCA4-Two pathogenic variants phase unknown; CRX-1 VOUS | ABCA4: AR Cone-rod dystrophy 3(MIM 604116); AR Fundus flavimaculatus(MIM 248200); AR Retinal dystrophy, early-onset severe(MIM 248200); AR Retinitis pigmentosa 19 (MIM 601718); AR Stargardt disease 1 (MIM 248200); CRX: AD Cone-rod retinal dystrophy-2(MIM 120970); AD, AR Leber congenital amaurosis 7 (MIM 613829) | ABCA4:2 heterozygous variants, phase unknown; CRX: Heterozygous | ABCA4 Chr1:[94508969G>A];[94528806A>G]; NM_000350.2:c.[3113C>T](;)[1622T>C]; NP_000341.2:p.[(Ala1038Val)](;)[(Leu541Pro)]  CRX; Chr19:[48339532A>G];[=]; NM_000554.5:c.[133A>G];[=] NP_000545.1:p.[(Thr45Ala)];[=] | Maculopathy | STGD | N.D. | Macular dystrophy | Likely Molecular Diagnosis |
| 94 | F | 31 | proband | Negative |  |  |  | N.D. | foveal hypoplasia, no dystrophy | ffERN normal; pattern ERG? | Macular dystrophy | Negative |
| 95 | F | 34 | proband | Negative |  |  |  | Rod-cone dystrophy | RP (severe) | N.D. | Rod-cone dystrophy | Negative |
| 96^#2^ | M | 66 | proband | 1 Likely pathogenic and VOUS, compound heterozygous | AR Leber congenital amaurosis 3(MIM 604232); AR Retinitis pigmentosa, juvenile (MIM 604232) | Compound heterozygous variants | SPATA7; Chr14:[88899497_88899498delTC];[88899496A>G]; NM_018418.4:c.[1102_1103delCT];[1100A>G]; NP_060888.2:p.[(Leu368GlufsTer4)];[(Tyr367Cys)] | Rod-cone dystrophy | RP (severe) | N.D. | Rod-cone dystrophy | Possible Molecular Diagnosis |
| 97 | F | 35 | proband | Negative |  |  |  | Rod-cone dystrophy | RP | N.D. | Rod-cone dystrophy | Negative |
| 98 | M | 41 | proband | Pathogenic | AD Night blindness, congenital stationary 1(MIM 610445); AR, AD Retinitis pigmentosa 4 (MIM 613731); AR, AD Retinitis punctata albescens (MIM 136880) | Heterozygous | RHO: Chr3:[129247842G>A];[=]; NM_000539.3:c.[266G>A];[=] NP_000530.1:p.[(Gly89Asp)];[=] | Rod-cone dystrophy | RP (severe) | N.D. | Rod-cone dystrophy | Molecular Diagnosis |
| 99 | F | 68 | proband | VOUS; 1 heterozygous variant in an AR disease; GUS | AR Cone-rod dystrophy 3(MIM 604116); AR Fundus flavimaculatus(MIM 248200); AR Retinal dystrophy, early-onset severe(MIM 248200); AR Retinitis pigmentosa 19 (MIM 601718); AR Stargardt disease 1 (MIM 248200) | ABCA4- heterozygous; AGBL1-heterozygous | ABCA4: Chr1:[94577093G>C];[=]; NM_000350.2:c.[203C>G];[=], NP_000341.2:p.[(Pro68Arg)];[=] AGBL1; Chr15:[86687064delT];[=]; NM_152336.3:c.[113delT];[=] NP_689549.3:p.[(Gly39AlafsTer1)];[=] | Maculopathy | Adult Vitelliform Macular Dystrophy | N.D. | Macular dystrophy | Other |
| 100 | F | 74 | proband | Negative |  |  |  | N.D. | AZOOR? | Rods/Cones non recordable on ffERG | Cone-rod dystrophy | Negative |
| 101 | F | 86 | proband | Negative |  |  |  | N.D. | Not RP | both rod/cones down on ffERG | Rod-cone dystrophy | Negative |
| 102 | M | 72 | proband | Pathogenic | AR, AD, Retinitis pigmentosa 1 (MIM 180100) | Heterozygous | RP1: Chr8:[55538721delTAAAT];[=];NM_006269.1:c.[2285_2289delTAAAT];[=]; NP_006260.1:p.[(Leu762Tyrfs*17)];[=] | Rod-cone dystrophy | RP | N.D. | Rod-cone dystrophy | Molecular Diagnosis |
| 103 | F | 72 | proband, Mother, Child (F) | Negative |  |  |  | Rod-cone dystrophy | RP | N.D. | Rod-cone dystrophy | Negative |
| 104 | M | 48 | proband | RPGR-VOUS; CRB1-1 Pathogenic Variant in an AR disease | Cone-rod dystrophy, X-linked, 1 (MIM 304020); Macular degeneration, X-linked atrophic(MIM 300834); Retinitis pigmentosa 3 (MIM 300029); Retinitis pigmentosa, X-linked, and sinorespiratory infections, with or without deafness (MIM 300455) | RPGR-Hemizygous; CRB1-Heterozygous | CRB1:Chr1:[197396856A>T];[=]; NM_201253.2:c.[2401A>T];[=] NP_957705.1:p.[(Lys801Ter)];[=] RPGR:ChrX:[38178097A>G];[0]; NM_001034853.1:c.[454T>C];[0] NP_001030025.1:p.[(Ser152Pro)];[0] | Rod-cone dystrophy | RP | N.D. | Rod-cone dystrophy | Possible Molecular Diagnosis |
| 105 | M | 59 | proband | VOUS | AD Choroidal dystrophy, central areolar 2(MIM:613105); AR, AD Leber congenital amaurosis 18 (MIM:608133); AD Macular dystrophy, patterned, 1(MIM 169150); AD Macular dystrophy, vitelliform, 3 (MIM:608161); AR, AD Retinitis pigmentosa 7 and digenic form (MIM 608133); AR, AD Retinitis punctata albescens (MIM 136880) | Heterozygous | PRPH2:Chr6:[42672308C>T];[=]; NM_000322.4::c.[623G>A];[=] NP_000313.2:p.[(Gly208Asp)];[=] | Maculopathy | Pattern dystrophy | Mild cone attenuation, rods normal | Macular dystrophy | Possible Molecular Diagnosis |
| 106 | F | 71 | proband, Father, Sibling (M) | Negative |  |  |  | Rod-cone dystrophy | RP (severe) | N.D. | Rod-cone dystrophy | Negative |
| 107 | M | 38 | proband | VOUS | AD Macular dystrophy, patterned, 2(MIM 608970) | Heterozygous | CTNNA1: Chr5:[138223228A>G];[=]; NM_001903.4:c.1193[A>G];[=], NP_001894.2p.[(Asn398Ser)];[=] | N.D. | N.D. | N.D. | Other | Possible Molecular Diagnosis |
| 108 | F | 59 | proband | Negative |  |  |  | Maculopathy | Pattern dystrophy | N.D. | Macular dystrophy | Negative |
| 109 | F | 32 | proband | 1 Pathogenic Variant in an AR disease | AR Retinitis pigmentosa 39 (MIM 613809); AR Usher syndrome, type 2A (MIM 276901) | Heterozygous | USH2A: Chr1:[216420460C>A];[=]; NM_206933.2:c.[2276G>T];[=] NP_996816.2:p.[(Cys759Phe)];[=] | Rod-cone dystrophy | RP | N.D. | Rod-cone dystrophy | Other |
| 110 | F | 51 | proband | Pathogenic heterozygous variant in AD disease | AD Retinitis pigmentosa 1 (OMIM:180100) | Heterozygous | RP1,Chr8:[55538721delTAAAT];[=], NM_006269.1:c.[2285_2289delTAAAT];[=], NP_006260.1:p.[( Leu762Tyrfs*17)];[=] | Rod-cone dystrophy | none | rod-cone dystrophy | Rod-cone dystrophy | Molecular Diagnosis |
| 111 | F | 54 | proband | Negative |  |  |  | Rod-cone dystrophy | RP | N.D. | Rod-cone dystrophy | Negative |
| 112 | M | 45 | proband | Negative |  |  |  | Rod-cone dystrophy | RP | N.D. | Rod-cone dystrophy | Negative |
| 113 | M | 62 | proband | Negative |  |  |  | Rod-cone dystrophy | RP (sectoral) | N.D. | Rod-cone dystrophy | Negative |
| 114 | M | 32 | proband | Negative |  |  |  | N.D. | N.D. | no ffERG | Other | Negative |
| 115 | M | 37 | proband | Negative |  |  |  | Rod-cone dystrophy | RP | N.D. | Rod-cone dystrophy | Negative |
| 116 | M | 55 | proband | Negative |  |  |  | Cone dystrophy vs Cone-rod dystrophy | N.D. | N.D. | Cone dystrophy | Negative |
| 117 | F | 75 | proband | Negative |  |  |  | Maculopathy | N.D. | N.D. | Other | Negative |
| 118 | F | 70 | proband | Negative |  |  |  | Rod-cone dystrophy | RP | N.D. | Rod-cone dystrophy | Negative |
| 119 | F | 71 | proband | Homozygous Pathogenic variant in AR disease | AR Retinitis pigmentosa 59(MIM 613861); AD Developmental delay and seizures with or without movement abnormalities (MIM 617836) | Homozygous | DHDDS: Chr1:[26764719A>G];[26764719A>G]; NM_205861.2:c.[124A>G];[124A>G]; NP_995583.1:p.[(Lys42Glu)];[(Lys42Glu) | Rod-cone dystrophy | End-stage RP (scalloped degen) | N.D. | Rod-cone dystrophy | Molecular Diagnosis |
| 120 | F | 54 | proband, Child (M) | VOUS | AD Retinitis pigmentosa 11 (MIM 600138) |  | PRPF31, Chr19[54632507C>T];[=]; NM_015629.4:c.[1222C>T];[=] NP_056444.3:p.[(Arg408Trp)];[(Arg408=)] | Rod-cone dystrophy | End-stage RP | N.D. | Rod-cone dystrophy | Possible Molecular Diagnosis |
| 121 | F | 43 | proband | 1 Likely Pathogenic Variant in an AR disease | AR Cone-rod dystrophy 3(MIM 604116); AR Fundus flavimaculatus(MIM 248200); AR Retinal dystrophy, early-onset severe(MIM 248200); AR Retinitis pigmentosa 19 (MIM 601718); AR Stargardt disease 1 (MIM 248200) | Heterozygous | ABCA4: Chr1:[94485278C>T];[=] , NM_000350.2:c.[5056G>A];[=] NP_000341.2:p.[(Val1686Met)];[=] | Rod-cone dystrophy | Sector RP | N.D. | Rod-cone dystrophy | Other |
| 122 | M | 67 | proband | Negative |  |  |  | Maculopathy | N.D. | N.D. | Macular dystrophy | Negative |
| 123 | F | 32 | proband | Homozygous Pathogenic variant in AR disease | AR Retinitis pigmentosa 59(MIM 613861); AD Developmental delay and seizures with or without movement abnormalities (MIM 617836) | homozygous | DHDDS:Chr1:[26764719A>G];[26764719A>G]; NM_205861.2:c.[124A>G];[124A>G]; NP_995583.1:p.[(Lys42Glu)];[(Lys42Glu)] | Rod-cone dystrophy | RP | N.D. | Rod-cone dystrophy | Molecular Diagnosis |
| 124 | F | 71 | proband | VOUS; VOUS | AD Retinitis pigmentosa 33 (MIM:610359); AD Retinitis pigmentosa 13 )MIM:600059) | Heterozygous | SNRNP200: Chr2:[96950142T>C];[=]; NM_014014.4:c.[4346A>G];[=]; NP_054733.2:p.[(Asn1449Ser)];[=]  PRPF8 Chr17:[1576647G>A];[=]; NM_0064453.3:c.3657+4C>T];[=] | Rod-cone dystrophy | EYS- or RHO-associated RP | N.D. | Rod-cone dystrophy | Possible Molecular Diagnosis |
| 125 | M | 29 | proband | Homozygous Pathogenic variants in AR disease | AR Retinitis pigmentosa 25 (MIM:602772) | homozygous | EYS: chr6:[65301640G>A];[65301640G>A]; NM_001142800.2:c.[4120C>T];[4120C>T]; NP_001136272.1:p.[(Arg1374Ter)];[(Arg1374Ter)] | Rod-cone dystrophy | RP | N.D. | Rod-cone dystrophy | Molecular Diagnosis |
| 126 | F | 64 | proband | Negative |  |  |  | Rod-cone dystrophy | RP (mild) | N.D. | Rod-cone dystrophy | Negative |
| 127 | F | 59 | proband | Negative |  |  |  | Maculopathy | N.D. | N.D. | Macular dystrophy | Negative |
| 128 | F | 77 | proband | VOUS | AD Cone dystrophy-3[MIM:602093]; AD Cone-rod dystrophy 14[MIM 602093] | heterozygous | GUCA1A;Chr6:[42147061C>T];[=]; NM_000409.4:c.[526C>T];[=]; NP_000400.2:p.[(Leu176Phe)];[=] | Maculopathy | Optical gap/Occult maculopathy | N.D. | Macular dystrophy | Possible Molecular Diagnosis |
| 129 | F | 57 | proband, Sibling (F), Sibling (M) | Homozygous Likely Pathogenic variants in AR disease | AR Retinitis pigmentosa 25 (MIM:602772) | homozygous | EYS:Chr6:[64791792G>T];[64791792G>T]; NM_001142800.1:c.[6528C>A][6528C>A]; NP_001136272.1p.[(Tyr2176Ter)];[(Tyr2176Ter)] | Rod-cone dystrophy | RP | N.D. | Rod-cone dystrophy | Molecular Diagnosis |
| 130 | M | 60 | proband | Negative |  |  |  | N.D. | N.D. | N.D. | Rod-cone dystrophy | Negative |
| 131 | M | 64 | proband | Negative |  |  |  | AZOOR | N.D. | N.D. | Other | Negative |
| 132 | F | 82 | proband | VOUS | AD Retinitis pigmentosa 11 (MIM 600138) | Heterozygous | PRPF31:Chr19:[54631568G>A];[=]; NM_015629.3:c.[1066G>A];[=] NP_056444.3:p.[(Gly356Ser)];[=] | Rod-cone dystrophy | RP | N.D. | Rod-cone dystrophy | Possible Molecular Diagnosis |
| 133 | M | 66 | proband | Negative |  |  |  | Rod-cone dystrophy | RP | N.D. | Rod-cone dystrophy | Negative |
| 134 | M | 61 | proband | 1 Pathogenic Variant in an AR disease; GUS | AR Retinitis pigmentosa 25 (MIM:602772) | EYS-heterozygous; GUS-heterozygous | EYS:Chr6:[65622398G>A];[=]; NM_001142800.1:c.[2620C>T];[=]; NP_001136272.1:p.[(Q874Ter)];[=] SEMA4A: Chr1:[156146474C>T];[=]; NM_022367:c.[1972C>T];[=]; NP_071762.2:p.[(Arg658Trp)];[=] | Rod-cone dystrophy | RP (severe) | N.D. | Rod-cone dystrophy | Other |
| 135 | F | 53 | proband, Mother(C1), Brother(C3) | One Likely Pathogenic variant, and One VOUS, phase unknown in a AR disease gene | AR Retinitis pigmentosa 14 (MIM 600132); AR Leber congenital amaurosis 15 (MIM 613843) | 2 heterozygous variants, phase unknown | TULP1:Chr6:[35479996dupTTCGGGG](;)[35471620T>C]; NM_003322.5:c.[146_147dupTTCGGGG](;)[1118A>G]; NP_003313.3:p.[(Pro49fs*126)](;)[(Asn373Ser)] | Cone-rod dystrophy | N.D. | N.D. | Cone-rod dystrophy | Likely Molecular Diagnosis |
| 136 | F | 74 | proband | Negative |  |  |  | Rod-cone dystrophy | RP | N.D. | Rod-cone dystrophy | Negative |
| 137 | F | 69 | proband | Negative |  |  |  | N.D. | STGD? | N.D. | Cone-rod dystrophy | Negative |
| 138 | M | 65 | proband | VOUS | Cone-rod dystrophy, X-linked, 1 (MIM 304020); Macular degeneration, X-linked atrophic(MIM 300834); Retinitis pigmentosa 3 (MIM 300029); Retinitis pigmentosa, X-linked, and sinorespiratory infections, with or without deafness (MIM 300455) | hemizygous | RPGR; ChrX:[38158399A>T];[0]; NM_000328.2:c.[1060-5T>A];[0] | Rod-cone dystrophy | RP (severe) | N.D. | Rod-cone dystrophy | Possible Molecular Diagnosis |
| 139 | M | 20 | proband,Mother (C1); unaffected brother (C3) | VOUS | AR, AD Exudative vitreoretinopathy 4 (MIM 601813); AD Hyperostosis, endosteal (MIM 144750); AD Osteopetrosis 1(MIM 607634); AR Osteoporosis-pseudoglioma syndrome (MIM259770); AD Osteosclerosis (MIM 144750); AD Polycystic liver disease 4 with or without kidney cysts (MIM 617875); AD van Buchem disease, type 2 (MIM 607636) | Heterozygous | LRP5:Chr11:[68183919A>G];[=]; NM_002335.3:c.[2951A>G];[=]; NP_002326.2:p.[(Tyr984Cys)];[=] | N.D. | N.D. | N.D. | Rod-cone dystrophy | Possible Molecular Diagnosis |
| 140 | M | 27 | proband | Negative |  |  |  | Rod-cone dystrophy | RP (severe) | N.D. | Rod-cone dystrophy | Negative |
| 141 | M | 24 | proband | One Pathogenic variant, and One VOUS, phase unknown (same gene) | AR Retinitis pigmentosa 28 (MIM 606068) | Two heterozygous Variants, Phase Unknown | FAM161A:Chr2:[62066830T>A](;)[62067406T>C]; NM_001201543.1:c.[1309A>T](;)[733A>G]; NP_001188472.1:p.[(Arg437Ter)](;)[(Met245Val)] | Rod-cone dystrophy | RP | N.D. | Rod-cone dystrophy | Likely Molecular Diagnosis |
| 142^#3^ | M | 11 | Trio | VOUS Compound heterozygous variants; GUS | AR Usher syndrome, type 2C (MIM 605472); AR Usher syndrome, type 2C, GPR98/PDZD7 digenic (MIM 605472) | USH2A: compound heterozygous variants; GUS: SCAPER - homozygous | ADGRV1 Chr5:[90001231G>A];[90085544G>A]; NM_032119.3:c.[8401G>A];[13919G>A] NP_115495.3:p.[Gly2801Arg];[Gly4649Glu].   SCAPER Chr15:[77021080T>C];[77021080T>C]; NM_020843.4:c.[2023-2A>G];[2023-2A>G] | Rod-cone dystrophy | RP | N.D. | Rod-cone dystrophy | Possible Molecular Diagnosis |
| 143 | F | 62 | proband | One Likely Pathogenic variant, and One VOUS, phase unknown (same gene) | AR Retinitis pigmentosa 39 (MIM 613809); AR Usher syndrome, type 2A (MIM 276901) | Two heterozygous Variants, Phase Unknown | USH2A:Chr1:[215848678C>T](;)[216373412T>C]; NM_206933.2:c.[12575G>A](;)[3368A>G]; NP_996816.2:p.[(Arg4192His)](;)[(Tyr1123Cys)] | Rod-cone dystrophy | RP | N.D. | Rod-cone dystrophy | Likely Molecular Diagnosis |
| 144 | M | 2 | proband | One Likely pathogenic variant in an AR disease | AR Joubert syndrome 5 (MIM 610188); Leber congenital amaurosis 10(MIM 611755); Meckel AR syndrome 4 (MIM 611134); AR Senior-Loken syndrome 6(MIM 610189) | Heterozygous | CEP290, Chr12:[88454772C>T];[=]; NM_025114.3:c.[6358-1G>A];[=] | Rod-cone dystrophy | LCA | N.D. | Rod-cone dystrophy | Other |
| 145 | F | 58 | proband | Negative |  |  |  | Maculopathy | Pattern dystrophy | N.D. | Macular dystrophy | Negative |
| 146 | M | 15 | Trio | Two Pathogenic variants identified, in trans | AR Cone-rod dystrophy 3(MIM 604116); AR Fundus flavimaculatus(MIM 248200); AR Retinal dystrophy, early-onset severe(MIM 248200); AR Retinitis pigmentosa 19 (MIM 601718); AR Stargardt disease 1 (MIM 248200) | Compound heterozygous variants | ABCA4:Chr1:[94476951A>G];[94508969G>A]; NM_000350.2:c.[3113C>T];[5461-10T>C]; NP_000341.2:p.[(Ala1038Val)];[(?)] | Maculopathy | STGD | N.D. | Macular dystrophy | Molecular Diagnosis |
| 147 | M | 83 | proband | Negative |  |  |  | Rod-cone dystrophy | RP | N.D. | Rod-cone dystrophy | Negative |
| 148 | F | 51 | proband | One pathogenic and one likely pathogenic variant, phase unknown | AR Retinitis pigmentosa 39 (MIM 613809); AR Usher syndrome, type 2A (MIM 276901) | Two heterozygous Variants, Phase Unknown | USH2A:Chr1:[216073486delA](;)[215821999G>A]; NM_206933.2:c.[7524delT](;)[14453C>T]; NP_996816.2:p.[(Arg2509fs)](;)[(Pro4818Leu)] | Rod-cone dystrophy | US | N.D. | Rod-cone dystrophy | Likely Molecular Diagnosis |
| 149 | F | 74 | proband | Negative |  |  |  | Rod-cone dystrophy | RP (severe) | N.D. | Rod-cone dystrophy | Negative |
| 150 | M | 21 | Trio | 2 VOUS in trans identified (same gene) | AR Retinitis pigmentosa 71(MIM 616394); AR Short-rib thoracic dysplasia 10 with or without polydactyly (MIM 615630) | Compound heterozygous variants | IFT172:Chr2:[27679408T>A];[27676334C>T]; NM_015662.3:c.[3341A>T];[3868G>A]; NP_056477.1:p.[(Glu1114Val)];[(Gly1290Arg)] | Rod-cone dystrophy | RP | N.D. | Rod-cone dystrophy | Possible Molecular Diagnosis |
| 151 | F | 9 | proband,Mother, Brother | 1 Likely Pathogenic variant in AR disease gene | AR Albinism, brown oculocutaneous (MIM 203200); AR Albinism, oculocutaneous, type II(MIM 203200) | Heterozygous | OCA2: Chr15:[28259941T>C];[=]; NM_000275.2:c.[1025A>G];[=], NP_000266.2:p.[Tyr342Cys];[=] | OCA | N.D. | N.D. | Other | Other |
| 152 | M | 57 | proband | 1 Pathogenic and 1 Likely Pathogenic variant identified, phase unknown | AR Cone-rod dystrophy 3(MIM 604116); AR Fundus flavimaculatus(MIM 248200); AR Retinal dystrophy, early-onset severe(MIM 248200); AR Retinitis pigmentosa 19 (MIM 601718); AR Stargardt disease 1 (MIM 248200) | Two heterozygous Variants, Phase Unknown | ABCA4, Chr1:[94544185C>T](;)[94512499T>C]; NM_000350.2:c.[1317G>A](;)[2894A>G]; NP_000341.2:p.[(Trp439Ter)](;)[(Asn965Ser)] | Maculopathy | STGD | N.D. | Macular dystrophy | Likely Molecular Diagnosis |
| 153 | M | 47 | proband | 1 Pathogenic and 1 VOUS identified, phase unknown | AR Retinitis pigmentosa 14 (MIM 600132); AR Leber congenital amaurosis 15 (MIM 613843) | Two heterozygous Variants, Phase Unknown | TULP1: Chr6:[35471401G>T](;)[35473548C>T]; NM_003322.5:c.[1082G>A](;)[1258C>A]; NP_003313.3:p.[(Arg361Gln)](;)[(Arg420Ser)] | Cone-rod dystrophy | Bull's eye maculopathy | N.D. | Cone-rod dystrophy | Possible Molecular Diagnosis |
| 154 | F | 54 | proband, Mother | Negative |  |  |  | N.D. | TULP1 or CRX genes? | N.D. | Macular dystrophy | Negative |
| 155* | M | 37 | proband | 2 Pathogenic variants identified, in trans | AR Joubert syndrome 5 (MIM 610188); Leber congenital amaurosis 10(MIM 611755); Meckel AR syndrome 4 (MIM 611134); AR Senior-Loken syndrome 6(MIM 610189) | Compound heterozygous variants | CEP290:Chr12:[88443069_88443073delCTTCT];[88508951delA]; NM_025114.3:c.[1833delT];[7328_7332delAGAAG]; NP_079390.3:p.[(Phe611fs)];[(Glu2443fs)] | Rod-cone dystrophy | RP or LCA | N.D. | Rod-cone dystrophy | Molecular Diagnosis |
| 156 | M | 50 | proband | Negative |  |  |  | Maculopathy | Optical gap/Occult maculopathy | N.D. | Macular dystrophy | Negative |
| 157 | M | 16 | proband, C1, C3 (unaffected brother) | Negative |  |  |  | Maculopathy | Benign fleck | N.D. | Macular dystrophy | Negative |
| 158 | M | 37 | proband | Negative |  |  |  | Rod-cone dystrophy | CHM | N.D. | Rod-cone dystrophy | Negative |
| 159 | F | 9 | proband | Negative |  |  |  | Maculopathy | Bull's eye maculopathy | N.D. | Macular dystrophy | Negative |
| 160 | M | 62 | Trio | Negative |  |  |  | Rod-cone dystrophy | RP | N.D. | Rod-cone dystrophy | Negative |
| 161 | F | 46 | proband, C3 | 2 VOUS | AR Retinitis pigmentosa 39 (MIM 613809); AR Usher syndrome, type 2A (MIM 276901) | Two heterozygous variants, phase unknown | USH2A: Chr1:[215802151C>G](;)[215963467A>T]; NM_206933.2:c.[10116T>A](;)[15519+5G>C]; NP_996816.2:p.[(Asn3372Lys)](;)[?] | Rod-cone dystrophy | RP | N.D. | Rod-cone dystrophy | Possible Molecular Diagnosis |
| 162 | F | 54 | proband | 1 Pathogenic, 1 VOUS identified, phase unknown | AR Cone-rod dystrophy 3(MIM 604116); AR Fundus flavimaculatus(MIM 248200); AR Retinal dystrophy, early-onset severe(MIM 248200); AR Retinitis pigmentosa 19 (MIM 601718); AR Stargardt disease 1 (MIM 248200) | Two heterozygous Variants, Phase Unknown | ABCA4:Chr1:[94473239T>G](;)[94544185C>T]; NM_000350.2:c.[5956T>G](;)[1317G>A]; NP_000341.2:p.[(Thr1986Pro)](;)[(Trp439Ter)] | Maculopathy | STGD | N.D. | Macular dystrophy | Possible Molecular Diagnosis |
| 163 | M | 50 | proband | 2 homozygous VOUSs in the SPATA7 gene | AR Leber congenital amaurosis 3(MIM 604232); AR Retinitis pigmentosa, juvenile (MIM 604232) | Homozygous (2 variants in the same gene) | SPATA7: Chr14:[88895702G>A;88904204A>G];[88895702G>A;88904204A>G]; NM_018418.4:c.[923G>A;1238A>G];[923G>A;1238A>G]; NP_060888.2:p.[(Cys308Tyr);(His413Arg)];[(Cys308Tyr);(His413Arg)] | Maculopathy or Cone-rod dystrophy | Bull's eye maculopathy | N.D. | Macular dystrophy | Possible Molecular Diagnosis |
| 164 | F | 50 | proband, C3 (son) | 1 VOUS | AD retinitis pigmentosa 33 (MIM#610359) | heterozygous | SNRNP200: Chr2:[96949648T>C];[=]; NM_014014.4:c.[4487A>G];[=]; NP_054733.2:p.[(Asn1496Ser)];[=] | Rod-cone dystrophy | RP | N.D. | Rod-cone dystrophy | Possible Molecular Diagnosis |
| 165 | M | 67 | proband | 1 Likely Pathogenic | AD Choroidal dystrophy, central areolar 2(MIM:613105); AR, AD Leber congenital amaurosis 18 (MIM:608133); AD Macular dystrophy, patterned, 1(MIM 169150); AD Macular dystrophy, vitelliform, 3 (MIM:608161); AR, AD Retinitis pigmentosa 7 and digenic form (MIM 608133); AR, AD Retinitis punctata albescens (MIM 136880) | Heterozygous | PRPH2: Chr6:[42672279A>G];[=]; NM_000322.4:c.[652T>C];[=]; NP_000313.2:p.[(Ser218Pro)];[=] | Maculopathy (severe) | Pattern dystrophy + Peripheral degeneration | Both rods/cones down | Macular dystrophy | Molecular Diagnosis |
| 166 | M | 51 | proband, C3 (cousin), C4 (brother) | negative |  |  |  | Rod-cone dystrophy | RP | N.D. | Rod-cone dystrophy | Negative |
| 167 | F | 27 | TRIO | 1 Pathogenic heterozygous variant in an autosomal recessive disorder | AR Retinitis pigmentosa 54 (MIM 613428) | 1 Heterozygous variant in a AR disease | PCARE: Chr2:[29294126C>T];[=]; NM_001029883.2:c.[3002G>A];[=], NP_001025054.1:p.[(Trp1001Ter)];[=] | Rod-cone dystrophy | RP (mild) | ffERG normal | Rod-cone dystrophy | Other |
| 168 | M | 92 | proband | 2 Likely pathogenic variants in trans in USH2A | AR Retinitis pigmentosa 39 (MIM 613809); AR Usher syndrome, type 2A (MIM 276901) | Compound heterozygous | USH2A:Chr1:[216143987C>A];[216143993delC]; NM_206933.2:c.[6931delG];[6937G>T]; NP_996816.2:p.[(Arg2311Profs*15)];[(Gly2313Cys)] | Rod-cone dystrophy | RP | N.D. | Rod-cone dystrophy | Molecular Diagnosis |
| 169 | F | 36 | proband | 1 Pathogenic, 1 VOUS identified, phase unknown | AR Retinitis pigmentosa 39 (MIM 613809); AR Usher syndrome, type 2A (MIM 276901) | 1 Pathogenic, 1 VOUS identified, phase unknown | USH2A; Chr1:[216251506C>T](;)[216595579G>GA]; NM_206933.2:c.[99_100insT](;)[5479G>A]; NP_996816.2:p.[(Arg34Serfs*41)](;)[(Val1833Met)] | Rod-cone dystrophy | RP | N.D. | Rod-cone dystrophy | Possible Molecular Diagnosis |
| 170 | F | 69 | proband | Negative |  |  |  | Maculopathy vs AMD | Late-onset maculopathy | N.D. | Macular dystrophy | Negative |
| 171 | M | 62 | proband | Negative |  |  |  | AMD vs. Maculopathy | N.D. | N.D. | Macular dystrophy | Negative |
| 172^ | M | 46 | Proband | 1 Likely Pathogenic | Cone-rod dystrophy, X-linked, 1 (MIM 304020); Macular degeneration, X-linked atrophic(MIM 300834); Retinitis pigmentosa 3 (MIM 300029); Retinitis pigmentosa, X-linked, and sinorespiratory infections, with or without deafness (MIM 300455) | Hemizygous | RPGR:ChrX:[38146098delG];[0]; NM_001034853.1:c.[2155delC];[0]; NP_001030025.1:p.[(His719Ilefs)];[0] | Rod-cone dystrophy | RP (severe) | N.D. | Rod-cone dystrophy | Molecular Diagnosis |
| 173 | M | 69 | proband | 1 Likely Pathogenic in a AD disease; 1 VOUS in a AR disease | TOPORS AD Retinitis pigmentosa 31 (MIM 609923); PCARE AR Retinitis pigmentosa 54 (MIM 613428) | Heterozygous; Heterozygous | TOPORS: Chr9:[32542000dupT];[=]; NM_005802.4:c.[2524dupA];[=], NP_005793.2:p.[(Thr842Asnfs)];[=]  PCARE: Chr2:[29293983dupG], NM_001029883.2:c.[3149dupC];[=], NP_001025054.1:p.[(Pro1051Thrfs)] | Rod-cone dystrophy | RP (severe) | N.D. | Rod-cone dystrophy | Molecular Diagnosis |
| 174 | F | 41 | proband, C1, C3 | Negative |  |  |  | N.D. | N.D. | N.D. | Other | Negative |
| 175 | F | 71 | proband | negative |  |  |  | Rod-cone dystrophy | RP | N.D. | Rod-cone dystrophy | Negative |
| 176 | F | 31 | proband | 1 VOUS | AD retinitis pigmentosa 18 (MIM#601414) | Heterozygous | PRPF3: Chr1:[150307684A>G];[=]; NM_004698.3:c.[1007A>G];[=], NP_004689.1:p.[(Glu336Gly)];[=] | Rod-cone dystrophy | RP | N.D. | Rod-cone dystrophy | Possible Molecular Diagnosis |
| 177 | F | 63 | proband | Negative |  |  |  | N.D. | N.D. | N.D. | Other | Negative |
| 178 | M | 59 | proband, C3 (daughter) | 1 hemizygous VOUS | Cone-rod dystrophy, X-linked, 1 (MIM 304020); Macular degeneration, X-linked atrophic(MIM 300834); Retinitis pigmentosa 3 (MIM 300029); Retinitis pigmentosa, X-linked, and sinorespiratory infections, with or without deafness (MIM 300455) | Hemizygous | RPGR: ChrX:[38144829C>A];[0]; NM_001034853.1:c.[3423G>T];[0]; NP_001030025.1:p.[(Trp1141Cys)];[0] | Maculopathy | Bull's eye maculopathy | N.D. | Macular dystrophy | Possible Molecular Diagnosis |
| 179 | F | 55 | proband, C1 (affected), C3 (unaffected) | negative |  |  |  | Maculopathy or AMD | Geographic atrophy (diffuse-trickling subtype) | N.D. | Macular dystrophy | Negative |
| 180 | F | 54 | proband, C3 (niece) | Negative |  |  |  | Maculopathy or AMD | Geographic atrophy (diffuse-trickling subtype) | N.D. | Macular dystrophy | Negative |
| 181 | F | 35 | proband | 2 heterozygous VOUSs in 2 different AR disease genes | EYS- AR Retinitis pigmentosa 25 (MIM:602772) ; PCARE AR Retinitis pigmentosa 54 (MIM 613428) | Heterozygous ; Heterozygous | PCARE: Chr2:[29296119C>T];[=]; NM_001029883.2:c.[1009G>A];[=], NP_001025054.1:p.[(Asp337Asn)];[=]  EYS:Chr6:[65149147T>C];[=]; NM_001142800.1:c.[5743A>G];[=], NP_001136272.1:p.[(Ser1915Gly)];[=] | Rod-cone dystrophy | RP | N.D. | Rod-cone dystrophy | Possible Molecular Diagnosis |
| 182 | F | 35 | proband | 1 Pathogenic, 2 VOUSs in the same gene;Three heterozygous variants, phase unknown; | CNGB1 - AR Retinitis pigmentosa 45 (MIM 613767) | Three heterozygous variants in the same gene , phase unknown | CNGB1:Chr16:[57931393Cdel](;)[57953019G>T](;)[58001027C>T]; NM_001297.4:c.[159+5G>A](;)[1941C>A](;)[3150delG]; NP_001288.3p.[?](;)[(Ser647Arg)](;)[(Phe1051Leufs*12)] | Rod-cone dystrophy | RP (mild) | N.D. | Rod-cone dystrophy | Possible Molecular Diagnosis |
| 183 | M | 22 | proband | Negative |  |  |  | Rod-cone dystrophy | LCA | N.D. | Rod-cone dystrophy | Negative |
| 184 | F | 76 | proband | Negative |  |  |  | Rod-cone dystrophy | RP | N.D. | Rod-cone dystrophy | Negative |
| 185*^#4^ | M | 13 | proband | 2 Likely pathogenic variants in trans in PROM1 | AD/AR Cone-rod dystrophy 12 (MIM:612657), AD Macular dystrophy, retinal, 2 (MIM:608051), AR Retinitis Pigmentosa 41 (MIM:612095), AD Stargardt disease 4 (MIM:603786) | compound heterozygous | PROM1:Chr4:[15993904delTA];[16077391delG], NM_006017.2:c.[1877_1878delTA](;)[139delC]; NP_006008.1:[(Ile626ArgfsTer6)];[(His47IlefsTer12)] | N.D. | N.D. | ffERG messy non-recordable, maybe rods down? | Cone-rod dystrophy | Molecular Diagnosis |
| 186 | F | 51 | proband | negative |  |  |  | Rod-cone dystrophy | RP (severe) | N.D. | Rod-cone dystrophy | Negative |
| 187 | F | 55 | proband | negative |  |  |  | Rod-cone dystrophy | RP | N.D. | Rod-cone dystrophy | Negative |
| 188 | M | 70 | proband | negative |  |  |  | Maculopathy or AMD | Geographic atrophy (diffuse-trickling subtype) | N.D. | Macular dystrophy | Negative |
| 189 | F | 49 | proband | 1 Pathogenic in AD disease | AD Choroidal dystrophy, central areolar 2(MIM:613105); AR, AD Leber congenital amaurosis 18 (MIM:608133); AD Macular dystrophy, patterned, 1(MIM 169150); AD Macular dystrophy, vitelliform, 3 (MIM:608161); AR, AD Retinitis pigmentosa 7 and digenic form (MIM 608133); AR, AD Retinitis punctata albescens (MIM 136880) | Heterozygous | PRPH2: Chr6:[42689649G>A];[=]; NM_00322.4:c.[424C>T];[=], NP_000313.2:p.[(Arg142Trp)];[=] | Maculopathy | Pattern dystrophy | N.D. | Macular dystrophy | Molecular Diagnosis |
| 190 | F | 78 | proband | negative |  |  |  | Drug-induced retinal toxicity | N.D. | N.D. | Other | Negative |
| 191 | M | 55 | proband | 1 Likely Pathogenic | AD Retinitis pigmentosa 11(MIM 600138) | Heterozygous | PRPF31:Chr19:[54629919C>T];[=], NM_015629.3:c.[872C>T];[=], NP_056444.3:p.[(Ala291Val)];[=] | Rod-cone dystrophy | RP | N.D. | Rod-cone dystrophy | Molecular Diagnosis |
| 192 | F | 4 | TRIO | negative |  |  |  | Rod-cone dystrophy | RP (severe) | N.D. | Rod-cone dystrophy | Negative |
| 193 | M | 34 | proband | 1 Pathogenic and 1 VOUS , in trans | AR, AD Leber congenital amaurosis 13 (MIM 612712) | Compound heterozygous | RDH12: Chr14:[68191306G>T];[68191267C>T]; NM_152443.2:c.[146C>T];[185G>T]; NP_689656.2:p.[(Arg62Leu)];[(Thr49Met)] | Maculopathy | Bull's eye maculopathy | ffERG normal | Macular dystrophy | Possible Molecular Diagnosis |
| 194 | F | 75 | proband | negative |  |  |  | AMD vs. Maculopathy | N.D. | N.D. | Macular dystrophy | Negative |
| 195 | F | 17 | proband | negative |  |  |  |  | RP | N.D. | Rod-cone dystrophy | Negative |
| 196 | F | 59 | proband | 1 homozygous VOUS | AR, AD, Retinitis pigmentosa 1 (MIM 180100) | Homozygous | RP1:Chr8:[55537952T>G];[55537952T>G]; NM_006269c.[1510T>G];[1510T>G]; , NP_006260.1:p.[(Ser504Ala)];[(Ser504Ala)] | Rod-cone dystrophy | RP | N.D. | Rod-cone dystrophy | Possible Molecular Diagnosis |
| 197 | F | 51 | proband | Homozygous VOUS | AR Retinitis pigmentosa 43 (MIM 613810) | Homozygous | PDE6A:Chr5:[149278951T>C];[149278951T>C]; NM_000440.2:c.[1250A>G];[1250A>G], NP_000431.2:p.[(Glu417Gly)];[(Glu417Gly)] | Rod-cone dystrophy | RP | N.D. | Rod-cone dystrophy | Possible Molecular Diagnosis |
| 198 | M | 86 | proband | Negative |  |  |  | Maculopathy | N.D. | N.D. | Macular dystrophy | Negative |
| 199 | F | 49 | proband | Negative |  |  |  | Rod-cone dystrophy | RP | N.D. | Rod-cone dystrophy | Negative |
| 200 | F | 50 | proband | 1 VOUS | AD/AR Cone-rod dystrophy 12 (MIM:612657), AD Macular dystrophy, retinal, 2 (MIM:608051), AR Retinitis Pigmentosa 41 (MIM:612095), AD Stargardt disease 4 (MIM:603786) | Heterozygous | PROM1: Chr4:[16025015C>T];[=]; NM_006017.2:c.[718G>A];[=]; NP_006008.1:p.[(Gly240Arg)];[=] | Rod-cone dystrophy | RP | N.D. | Rod-cone dystrophy | Possible Molecular Diagnosis |
| 201 | M | 67 | proband | Negative |  |  |  | Rod-cone dystrophy | RP | N.D. | Rod-cone dystrophy | Negative |
| 202 | F | 69 | proband, C5 (cousin) | Negative |  |  |  | Maculopathy or Cone-rod dystrophy | N.D. | rods normal on ffERG, cones decreased | Other | Negative |
| 203 | F | 34 | P, C3(sister), C4(son) | pathogenic variant (homozygous) | AR, DR Bardet-Biedl syndrome 1 (MIM 209900) | Homozygous | BBS1:Chr11:[66293652T>G];[66293652T>G]; NM_024649.4:c.[1169T>G];[1169T>G]; NP_078925:p.[(Met390Arg)];[(Met390Arg)] | Rod-cone dystrophy | RP | N.D. | Rod-cone dystrophy | Molecular Diagnosis |
| 204 | M | 49 | proband | Negative |  |  |  | N.D. | Paravenous retinal dystrophy | N.D. | Rod-cone dystrophy | Negative |
| 205 | M | 26 | proband, father | Negative |  |  |  | Rod-cone dystrophy | RP | N.D. | Rod-cone dystrophy | Negative |
| 206 | F | 16 | TRIO | 1 Pathogenic variant in autosomal recessive disease identified | AR Leber congenital amaurosis 5 (MIM:604537) | heterozygous variant in AR disease | LCA5; Chr6:[80203353G>A];[=]; NM_181714.3:c.[835C>T];[=]; NP_859065.2:p.[(Gln279Ter)];[=] | N.D. | N.D. | N.D. | Other | Other |
| 207 | F | 55 | proband | Likely Pathogenic (homozygous) | AR Retinal cone dystrophy 4 (MIM 610478) | homozygous | CACNA2D4: Chr12:[1969369G>A];[1969369G>A]; NM_172364.4:c.[1882C>T];[1882C>T]; NP_758952.4:p.[(Arg628Ter)];[(Arg628Ter)] | Maculopathy vs Cone-rod dystrophy | N.D. | ffERG cones abnormal | Cone dystrophy | Molecular Diagnosis |
| 208 | M | 49 | proband | Negative |  |  |  | Maculopathy | Macular atrophy | Maculopathy | Macular dystrophy | Negative |
| 209 | F | 58 | proband | Likely Pathogenic Variant | AD Retinitis pigmentosa 13 (MIM 600059) | Heterozygous | PRPF8; Chr17:[1554142_ 1554157delTGCAGGAGAGCAAAGT];[=]; NM_006445.3:c.[6947_6962delACTTTGCTCTCCTGCA];[=]; NP_006436.3:p.[(Asn2316ArgfsTer38)];[(Asn2316ArgfsTer38)] | Maculopathy and Retinitis pigmentosa | With Cystoid macular edema | Rod non-recordable suggesting retinitis pigmentosa. Cone attenuated. | Macular dystrophy | Molecular Diagnosis |
| 210 | M | 49 | proband | 1 Pathogenic and 1 VOUS in the same gene , phase unknown | AR Cone-rod dystrophy 3(MIM 604116); AR Fundus flavimaculatus(MIM 248200); AR Retinal dystrophy, early-onset severe(MIM 248200); AR Retinitis pigmentosa 19 (MIM 601718); AR Stargardt disease 1 (MIM 248200) | Two heterozygous Variants, Phase Unknown | ABCA4;Chr1:[94517254C>G](;)[94528713C>T]; NM_000350.2c.[2588G>C](;)[1715G>A]; NP_000341.2:p.[(Gly863Ala)](;)[(Arg572Gln)] | Maculopathy | With flecks | normal | Macular dystrophy | Possible Molecular Diagnosis |
| 211 | F | 80 | proband | Negative |  |  |  | Rod-cone | RP | Rod-cone | Rod-cone dystrophy | Negative |
| 212 | M | 26 | proband | 2 pathogenic, phase unknown | Cone Rod Dystrophy 3 (OMIM#604116), Retinal dystrophy, early-onset severe (OMIM# 248200), Retinitis Pigmentosa 19 (OMIM#601718), and Stargardt Disease 1 (OMIM# 248200) | 2 heterozygous, phase unknown | ABCA4; Chr1:[94473807C>T](;)[94496666G>A]; NM_000350.2:c.[4139C>T](;)[5882G>A]; NP_000341.2:p.[(Pro1380Leu)](;)[(Gly1961Glu)] | Maculopathy | Adult Vitelliform Macular Dystrophy | maculopathy | Macular dystrophy | Possible Molecular Diagnosis |
| 213 | F | 35 | proband, mother (affected) | VOUS | Occult macular dystrophy (AD, OMIM#613587) | Heterozygous | RP1L1; Chr8:[10480498C>T];[=]; NM_178857.5:c.[214G>A];[=] NP_849188.4:p.[(Val72Met)];[=] | Maculopathy | N.D. | ffERG not performed | Macular dystrophy | Possible Molecular Diagnosis |
| 214 | F | 51 | proband | VOUS | AD Cone-rod dystrophy 5 [MIM: 600977] | Heterozygous | PITPNM3; chr17:[6406892delCTC];[=]; NM_031220.3:c.[227_229delGAG];[=]; NM_031220.3:p.[(Gly76del)];[=] | cone-rod | N.D. | cone-rod | Cone-rod dystrophy | Possible Molecular Diagnosis |
| 215 | M | 50 | proband | Negative |  |  |  | cone dystrophy | N.D. | cone dystrophy | Cone dystrophy | Negative |
| 216 | M | 17 | proband | Pathogenic variant identified | X-linked recessive retinitis pigmentosa (OMIM#300029) | hemizygous | RPGR;ChrX:[38145928delCT];[0]; NM_001034853.1:c.[2323_2324delAG];[0]; NP_001030025.1:p.[(Arg775Glufs)];[0] | Rod-cone dystrophy | RP (severe) | Non-recordable responses | Rod-cone dystrophy | Molecular Diagnosis |
| 217 | M | 34 | TRIO | One Pathogenic and one Likely Pathogenic variant present in trans, identified | AR Cone-rod dystrophy 3 (OMIM: 604116); AR Fundus flavimaculatus (OMIM: 248200); AR Retinal dystrophy, early-onset severe (OMIM: 248200); AR Retinitis Pigmentosa 19 (OMIM: 601718); AR Stargardt Disease I (OMIM: 248200); AD Macular degeneration, age-related, 2 (OMIM: 153800). | Compound Heterozygous | ABCA4; Chr1:[94476355C>G];[94473807C>T]; NM_000350.2:c.[5714+1G>C];[5882G>A]; NP_000341.2:p.[(?)];[(Gly1961Glu)] | Maculopathy | STGD | Cone-rod | Macular dystrophy | Molecular Diagnosis |
| 218 | M | 29 | TRIO | Compound heterozygous pathogenic variants identified | Cone-rod dystrophy, type 3 (OMIM# 604116); AR Fundus flavimaculatus (OMIM# 248200); AR Retinal dystrophy, early-onset severe (OMIM# 248200); AR Retinitis pigmentosa, type 19 (OMIM# 601718); AR Stargardt disease, type 1 (OMIM# 248200); AD Macular degeneration, age related, type 2 (OMIM# 153800) | Compound Heterozygous | ABCA4; Chr1:[94508969G>A];[94508434dupCA]; NM_000350.2:c.[3113C>T];[3210_3211dupGT]; NP_000341.2:p.[(Ala1038Val)];[(Ser1071Cysfs)] | Maculopathy | STGD | **Normal**: No detectable dysfunction | Macular dystrophy | Molecular Diagnosis |
| 219 | F | 23 | TRIO | Pathogenic variant identified | Autosomal Recessive and Autosomal Dominant Pseudoxanthoma Elasticum (OMIM# 264800) | Homozygous | ABCC6; Chr16:[16256935G>A];[16256935G>A]; NM_001171.5:c.[3421C>T];[3421C>T]; NP_001162.4:p.[(Arg1141Ter)];[(Arg1141Ter)] | Healthy | N.D. | N.D. | Other | Molecular Diagnosis |
| 220 | F | 61 | proband | Pathogenic Variant Identified | Autosomal Recessive and Autosomal Dominant Pseudoxanthoma Elasticum (OMIM# 264800) | Homozygous | ABCC6; Chr16:[16256935G>A];[16256935G>A]; NM_001171.5:c.[3421C>T];[3421C>T]; NP_001162.4:p.[(Arg1141Ter)];[(Arg1141Ter)] | Maculopathy | Pseudoxanthoma elasticum | N.D. | Other | Molecular Diagnosis |
| 221 | F | 27 | proband, mother | Homozygous likely pathogenic variant | Autosomal Recessive Joubert syndrome 3 (OMIM#: 608629) | Homozygous | AHI1; Chr6:[135748398G>A];[135748398G>A]; NM_001134830.1:c.[2671C>T];[2671C>T]; NP_001128302.1:p.[(Arg891Ter)];[(Arg891Ter)] | Rod-cone dystrophy | RP (atypical) | Non-recordable responses | Rod-cone dystrophy | Molecular Diagnosis |
| 222 | M | 63 | proband | Heterozygous likely pathogenic variant | autosomal recessive bestrophinopathy (OMIM#: 611809), autosomal dominant vitelliform macular dystrophy 2 (OMIM#: 153700), autosomal dominant vitreoretinochoroidopathy (OMIM#: 193220), retinitis pigmentosa 50 (OMIM#: 613194) | Heterozygous | BEST1; Chr11:[61730104_61730107del];[=]; NM_001139443.1:c.[1300_1303delGGCA];[=]; NP_001132915:p.[(Gly434Ter)];[=] | Rod-cone dystrophy | RP (severe) | N.D. | Rod-cone dystrophy | Molecular Diagnosis |
| 223 | M | 8 | proband, father | Likely pathogenic variant | Congenital stationary night blindness, type 2A (incomplete), X-linked (XLR, OMIM#300071); Cone-rod dystrophy, X-linked, 3 (XLR, OMIM#300476); Aland Island eye disease (XLR, OMIM#300600) | Hemizygous | CACNA1F; ChrX:[49072841C>T];[0]; NM_001256789.2:c.[3236+1G>A];[0] | Healthy | N.D. | Cone-rod (data is very messy; cone attenuation may not be real) | Other | Molecular Diagnosis |
| 224^#5^ | F | 22 | proband, mother | Pathogenic variant identified | Choroideremia (OMIM#303100) | Heterozygous | CHM; ChrX:[85213970G>A];[=]; NM_000390.3:c.[715C>T];[=]; NP_000381.1:p.[(Arg239Ter)];[=] | Rod-cone dystrophy | CHM | N.D. | Other | Molecular Diagnosis |
| 225^#5^ | F | 64 | proband | Pathogenic variant, heterozygous | X-linked disease Choroideremia (OMIM#303100) | Heterozygous | CHM; ChrX:[8513992_85134001del];[=]; NM_000390.3c.[1584_1587delTGTT];[1584=];p.[(Val529Hisfs*7)];[Val529=] (, NP_000381.1) | X-linked choroideremia carrier | N.D. | N.D. | Other | Molecular Diagnosis |
| 226 | M | 42 | proband | Likely Pathogenic Variant identified | Autosomal Recessive Retinitis Pigmentosa 45 (OMIM# 613767) | Homozygous | CNGB1; Chr16:[57949173G>A];[57949173G>A]; NM_001297.4:c.[2284C>T];[2284C>T]; NP_001288.3:p.[(Arg762Cys)];[(Arg762Cys)] | Rod-cone dystrophy | RP (severe) | Non-recordable responses | Rod-cone dystrophy | Molecular Diagnosis |
| 227^#6^ | M | 25 | proband; C3;C4 | Two Pathogenic and Likely Pathogenic variants identified | Coenzyme Q10 deficiency, primary, 1 (OMIM#609825) | Compound heterozygous | COQ2; Chr4:[84205780dupG];[84205692G>C]; NM_015697.7:c.[288dupC];[376C>G]; NP_056512.5:p.[(Ala97Argfs)];[(Arg126Gly)] | Rod-cone dystrophy | RP (atypical) | Rod-cone | Rod-cone dystrophy | Molecular Diagnosis |
| 228 | M | 45 | proband | Heterozygous pathogenic variants in two genes (1 AD gene and the 2nd one AR gene) | Cone-rod retinal dystrophy 2 (AD, OMIM#120970), Leber congenital amaurosis 7 (AR, | Heterozygous | CRX; Chr19:[48342773C>G];[=]; NM_000554.5:c.[449C>G];[=]; NP_000545.1:p.[(Ser150Ter)];[=] | Maculopathy | Bull's maculopathy | N.D. | Macular dystrophy | Molecular Diagnosis |
| 229 | F | 66 | proband | Pathogenic variant identified | autosomal dominant Doyne honeycomb degeneration of retina (OMIM#126600) | Heterozygous | EFEMP1; Chr2:[56098226 G>A];[=]; NM_001039348.2:c.[1033C>T];[=]; NP_001034437.1:p.[(Arg345Trp)];[=] | Maculopathy | Doyne Honeycomb Dystrophy (Malattia Leventinese) | N.D. | Macular dystrophy | Molecular Diagnosis |
| 230 | F | 64 | proband | Pathogenic Variant Identified | Retinitis pigmentosa 28 (OMIM#606068) | Homozygous | FAM161A;Chr2:[62066783_62066784del];[62066783_62066784del]; NM_001201543.2:c.[1355_1356delCA];[1355_1356delCA] NP_001188472.1:p.[(Thr452Serfs)];[(Thr452Serfs)] | Rod-cone dystrophy | RP (severe) | N.D. | Rod-cone dystrophy | Molecular Diagnosis |
| 231 | F | 9 | proband | Mosaic Pathogenic variant identified | X-linked recessive ocular albinism, type I, Nettleship-Falls type (OMIM#300500) | mosaic (VAF = 21.4%; 39/182 reads) | GPR143; ChrX:[9711715=/T>G]; NM_000273.2:c.[659-2=/A>C] | X-linked OA1 carrier | N.D. | **Normal**: No detectable dysfunction* (although minor cone attenuation seen) | Other | Molecular Diagnosis |
| 232 | F | 28 | Proband & affected mother & unaffected brother | Heterozygous Pathogenic variant identified | Cone dystrophy-3 (AD, OMIM#602093), Cone-rod dystrophy 14 (AD, OMIM#602093) | Heterozygous | GUCA1A; Chr6:[42146112A>G];[=]; NM_000409.4:c.[296A>G];[=]; NP_000400.2p.[(Tyr99Cys)];[=] | Cone dystrophy vs. Cone-rod dystrophy | Bull's eye maculopathy | Cone | Cone dystrophy | Molecular Diagnosis |
| 233 | M | 48 | proband | Pathogenic variant identified | AR Retinitis Pigmentosa 69 (OMIM#: 615780) | homozygous | KIZ; Chr20:[21117104C>T];[21117104C>T]; NM_018474.5:c.[226C>T];[226C>T]; NP_060944.3:p.[(Arg76Ter)];[(Arg76Ter)] | Rod-cone dystrophy | RP (severe) | Rod-cone | Rod-cone dystrophy | Molecular Diagnosis |
| 234 | M | 66 | P:C3(BROTHER); C4(BROTHER AFFECTED), | 1 Likely Pathogenic Variant identified | Retinitis Pigmentosa 42, (OMIM#612943) | Heterozygous | KLHL7; Chr7:[23180417T>C];[=]; NM_001031710.2:c.[472T>C];[=]; NP_001026880.2:p.[(Cys158Arg)];[=] | Rod-cone dystrophy | RP | Rod-cone | Rod-cone dystrophy | Molecular Diagnosis |
| 235* | F | 52 | proband :C3 | Pathogenic variant identified | Retinitis pigmentosa 37, AR, AD (OMIM# 611131); Enhanced S-cone syndrome, AR (OMIM# 268100). | Homozygous | NR2E3; Chr15:[72103821A>C];[72103821A>C]; NM_016346.3:c.[119-2A>C];[119-2A>C] | Rod-cone dystrophy | Enhanced S-cone syndrome | Rod-cone | Rod-cone dystrophy | Molecular Diagnosis |
| 236 | M | 19 | proband;C1,C3 | Pathogenic variant identified | X-linked Recessive Blue Cone Monochromacy (OMIM# 303700) | Hemizygous | OPN1MW; Chr X:[153457207T>C];[0]; NM_000513.2:c.[607T>C];[0]; NP_000504.1:p.[(Cys203Arg)];[0] | Cone dystrophy | Achromatopsia | Cone | Cone dystrophy | Molecular Diagnosis |
| 237 | F | 11 | TRIO | Pathogenic variant identified | Usher syndrome, type 1F (MIM# 602083) | Homozygous | PCDH15; Chr10:[56077174G>A];[56077174G>A]; NM_033056.3:c.[733C>T];[733C>T]; NP_149045.3:p.[(Arg245Ter)];[(Arg245Ter)] | Rod-cone dystrophy | US | Non-recordable responses | Rod-cone dystrophy | Molecular Diagnosis |
| 238 | F | 31 | proband | Likely Pathogenic variant identified | Night blindness, congenital stationary, autosomal dominant 2 (OMIM#163500); Retinitis  pigmentosa-40, autosomal recessive (OMIM#613801) | Homozygous | PDE6B; Chr4:[657977T>G];[657977T>G]; NM_000283.3:c.[2096T>G];[2096T>G]; NP_000274.2:p.[(Leu699Arg)];[(Leu699Arg)] | Rod-cone dystrophy | RP | Non-recordable responses | Rod-cone dystrophy | Molecular Diagnosis |
| 239 | F | 12 | TRIO | Pathogenic Variant identified | Cone-rod dystrophy 12 (OMIM#3612657), Autosomal Dominant Retinal macular dystrophy 2 (OMIM#608051), Autosomal Recessive Retinitis pigmentosa 41 (OMIM#612095), and Stargardt disease 4 (OMIM#603786) | Homozygous | PROM1; Chr4:[16010732C>T];[16010732C>T]; NM_006017.2: c.[1142-1G>A];[1142-1G>A] | Cone-rod dystrophy | Bull's eye maculopathy | Cone-rod | Cone-rod dystrophy | Molecular Diagnosis |
| 240 | M | 38 | proband | Pathogenic variant identified | Autosomal Dominant Retinitis Pigmentosa (OMIM #600059) | Heterozygous | PRPF8; Chr17:[1554192G>C];[=]; NM_006445.3:c.[6912C>G];[=]; NP_006436.3:p.[(Phe2304Leu)];[=] | Rod-cone dystrophy | RP | N.D. | Rod-cone dystrophy | Molecular Diagnosis |
| 241 | M | 51 | proband | Pathogenic variant identified | Autosomal Dominant Macular dystrophy, vitelliform, 3 (OMIM #608161) | Heterozygous | PRPH2; Chr6:[42689960del];[=]; NM_000322.4:c.[113delG];[=]; NP_000313.2:p.[(Gly38Aspfs)];[=] | Maculopathy | Pattern dystrophy | Cone-rod | Macular dystrophy | Molecular Diagnosis |
| 242 | F | 64 | Proband + Brother + Sister | Likely Pathogenic variant identified | central areolar 2 choroidal dystrophy (OMIM#613105), leber congenital amaurosis 18 (OMIM#608133), patterned macular dystrophy 1 (OMIM#169150), vitelliform macular dystrophy 3 (OMIM#608161), retinitis pigmentosa 7 and digenic form (OMIM#608133), and retinitis punctata albescens (OMIM#136880) | heterozygous | PRPH2; Chr6:[42672277CG>G];[=]; NM_000322.4:c.[654delG];[=]; NP_000313.2:p.[(Pro219Hisfs)];[=] | Cone-rod dystrophy vs. Maculopathy | Pattern dystrophy | Cone-rod | Cone-rod dystrophy | Molecular Diagnosis |
| 243 | F | 43 | proband & affected father & affected sibling | 1 Pathogenic Variant identified; Inheritance: Paternal | AD Night blindness, congenital stationary, 1 (OMIM# 610445), AD or AR Retinitis pigmentosa 4 (OMIM# 613731), AD or AR Retinitis punctata albescens (OMIM# 136880) | Heterozygous | RHO; Chr3:[129247842G>A];[=]; NM_000539.3:c.[266G>A];[=]; NP_000530.1:p.[(Gly89Asp)];[=] | Rod-cone dystrophy | RP (severe) | Non-recordable responses | Rod-cone dystrophy | Molecular Diagnosis |
| 244 | F | 55 | proband | Heterozygous Pathogenic Variant Detected | AD, AR Retinitis Pigmentosa 1 (OMIM#: 180100) | Heterozygous | RP1; Chr8:[55538471C>T];[=]; NM_006269.1:c.[2029C>T];[=]; NP_006260.1:p.[(Arg677Ter)];[=] | Rod-cone dystrophy | RP | Non-recordable responses | Rod-cone dystrophy | Molecular Diagnosis |
| 245 | F | 50 | proband | Heterozygous pathogenic variant identified | Retinitis pigmentosa 1(OMIM#180100,AD,AR) | Heterozygous | RP1; Chr8:[55538471C>T];[=]; NM_006269.1:c.[2029C>T];[=]; NP_006260.1:p.[(Arg677Ter)];[=] | Rod-cone dystrophy | Sectoral RP | Rod-cone | Rod-cone dystrophy | Molecular Diagnosis |
| 246 | M | 45 | proband | Likely pathogenic variant identified | Occult macular dystrophy (AD, OMIM#613587) | Heterozygous | RP1L1; Chr8:[10480579G>A];[=]; NM_178857.5:c.[133C>T];[=]; NP_849188.4:p.[(Arg45Trp)];[=] | Maculopathy | Occult macular dystrophy | Cone-rod | Macular dystrophy | Molecular Diagnosis |
| 247 | M | 33 | proband | Likely Pathogenic variant identified | X-linked recessive retinitis pigmentosa 3 (OMIM#300029) | hemizygous | RPGR; ChrX:[38146245C>T];[0]; NM_001034853.1:c.[2007G>A];[0]; NP_001030025.1:p.[(Trp669Ter)];[0] | Rod-cone dystrophy | RP | Non-recordable responses | Rod-cone dystrophy | Molecular Diagnosis |
| 248^ | M | 59 | proband; C3 | Pathogenic Variant Identified | X-linked recessive Retinitis Pigmentosa 3 (OMIM#300029) | hemizygous | RPGR; ChrX:[38145846delCT];[0]; NM_001034853.1:c.[2405_2406delAG];[0]; NP_001030025.1:p.[(Glu802Glyfs )];[0] | Rod-cone dystrophy | RP (severe) | N.D. | Rod-cone dystrophy | Molecular Diagnosis |
| 249 | M | 32 | proband | Likely pathogenic variant identified | Foveal hypoplasia 2, with or without optic nerve misrouting and/or anterior segment dysgenesis | Homozygous | SLC38A8; Chr16:[84075603C>A];[84075603C>A]; NM_001080442.2:c.[160G>T];[160G>T]; NP_001073911.1:p.[(Gly54Ter)];[(Gly54Ter)] | Ocular albinism | N.D. | White | Other | Molecular Diagnosis |
| 250 | M | 72 | proband | Likely pathogenic variant identified | Retinitis pigmentosa 22 (AD, OMIM#610359) | Heterozygous | SNRNP200; Chr2:[96958829G>A];[=]; NM_014014.4:c.[2041C>T];[=]; NP_054733.2:p.[(Arg681Cys)];[=] | Rod-cone dystrophy | RP | Non-recordable responses | Rod-cone dystrophy | Molecular Diagnosis |
| 251 | F | 58 | proband | One Likely Pathogenic variant identified in a gene related to the patient’s phenotype | Retinitis pigmentosa 33, AD (OMIM# 610359) | Heterozygous | SNRNP200; Chr2:[96953706G>A];[=]; NM_014014.4:c.[3260C>T];[=]; NP_054733.2:p.[(Ser1087Leu)];[=] | Rod-cone dystrophy | RP (severe) | Non-recordable responses | Rod-cone dystrophy | Molecular Diagnosis |
| 252 | F | 68 | proband | Likely pathogenic variant identified | Retinitis pigmentosa 39 (AR, OMIM#613809), Usher Syndrome, type 2A (AR, OMIM#276901) | Homozygous | USH2A; Chr1:[215848678C>T];[215848678C>T]; NM_206933.2:c.[12575G>A];[12575G>A]; NP_996816.2:p.[(Arg4192His)];[(Arg4192His)] | Rod-cone dystrophy | RP (severe) | N.D. | Rod-cone dystrophy | Molecular Diagnosis |
| 253* | M | 56 | proband | 1 Likely pathogenic variant and 1 pathogenic variant in trans | AR, Retinitis Pigmentosa 54 (OMIM: 613425). | compound heterozygous | PCARE, chr2:[29293779_29293782del];[29295400_29295419del]; NM_001029883.3:c.[3346_3349delTCTG];[1709_1728delTGGGGGGACCACTGTCCTCC]; NP_001025054.1:p.[(Ser1116Glyfs*27)];[(Gly570Glufs*3)] | Rod-cone dystrophy | RP | N.D. | Rod-cone dystrophy | Molecular Diagnosis |
| 254 | M | 73 | proband | 1 pathogenic and 1 likely pathogenic variant, (phase unknown) | AR, Cone Rod Dystrophy 3 (OMIM#604116), AR, Retinal dystrophy, early-onset severe (OMIM# 248200), AR, Retinitis Pigmentosa 19 (OMIM#601718), AR, Stargardt Disease 1 (OMIM# 248200) | two heterozygous variants | ABCA4; Chr1:[94508323G>A](;)[94526296G>A]; NM_000350.2:c.[3322C>T](;)[1957C>T]; NP_000341.2:p.[(Arg1108Cys)](;)[(Arg653Cys)] | Cone-rod dystrophy | STGD | N.D. | Cone-rod dystrophy | Possible Molecular Diagnosis |
| 255 | F | 65 | proband | 1 Likely Pathogenic and 1 Pathogenic Variant, (phase unknown) | AR, Achromatopsia 2 (OMIM#216900) | two heterozygous variants | CNGA3; Chr2[99008427C>G](;)[99013302G>A]; NM_001298.2:c.[667C>G](;)[1669G>A]; NP_001289.1:p.[(Arg223Gly)](;)[(Gly557Arg)] | Maculopathy | STGD | N.D. | Macular dystrophy | Possible Molecular Diagnosis |
| 256* | F | 76 | proband | Compound heterozygous | AR Cone-rod dystrophy 15 (OMIM#613660); AR Retinitis pigmentosa 65 (OMIM#613660) | two heterozygous variants | CDHR1; chr10[85962879G>A]](;)[85968083C>T]; NM_033100.3:c.[783G>A](;)[1117C>T]; NP_149091.1:p.[(Pro261=)](;)[(Gln373Ter)] | Hereditary retinal dystrophy | Cone-rod dystrophy | N.D. | Cone-rod dystrophy | Likely Molecular Diagnosis |
| 257 | F | 22 | proband/C3 | 1 pathogenic and 1 likely pathogenic variant (phase unknown) | AR, Leber congenital amaurosis 2 (OMIM#204100), AR, Retinitis pigmentosa 20 (OMIM#613794) | two heterozygous variants (in both proband and affected sister) | RPE65; Chr1:[68897192C>T](;)[68903976A>G]; NM_000329.2:c.[1205G>A](;)[1022T>C]; NP_000320.1:p.[(Trp402Ter)](;)[(Leu341Ser)] | Rod-cone dystrophy | RP (severe) | Non-recordable responses | Rod-cone dystrophy | Possible Molecular Diagnosis |
| 258 | M | 28 | proband | 2 likely pathogenic variants, (phase unknown) | AR, Retinitis pigmentosa 39 (OMIM#613809), AR, Usher Syndrome, type 2A (OMIM#276901) | two heterozygous variants | USH2A; Chr1:[215848678C>T](;)[216419934A>C]; NM_206933.2:c.[12575G>A](;)[2802T>G]; NP_996816.2:p.[(Arg4192His)](;)[(Cys934Trp)] | Rod-cone dystrophy | US | Amplitudinal attenuation of rod and cone responses | Rod-cone dystrophy | Possible Molecular Diagnosis |
| 259 | M | 22 | proband, mother | 2 pathogenic variants (phase unknown) | AR, Knobloch syndrome, type 1 (OMIM#267750) | two heterozygous variants | COL18A1; chr21:[46895403_46895404del](;)[46917565dup]; NM_030582.3:c.[1292_1293delCT](;)[3213dupC]; NP_085059.2:p.[(Ser431Trpfs)](;)[(Gly1072Argfs)] | Rod-cone dystrophy | syndromic RP (Knobloch syndrome) | Non-recordable responses | Rod-cone dystrophy | Possible Molecular Diagnosis |
| 260 | F | 25 | proband, mother | 2 Pathogenic Variants (phase unknown) | AR, Cone-rod Dystrophy (OMIM# 604116) AR, Stargardt Disease (OMIM #248200) | two heterozygous, variants are likely in trans | ABCA4; chr1:[94564484G>A](;)[94473807C>T]; NM_000350.2:c.[634C>T](;)[5882G>A]; NP_000341.2:p.[(Arg212Cys)](;)[(Gly1961Glu)] | Maculopathy | STGD | Cone-rod | Macular dystrophy | Possible Molecular Diagnosis |
| 261 | M | 22 | proband | 2 Likely pathogenic variants, (phase unknown) | AR, Retinitis pigmentosa 39 (OMIM# 613809); AR, Usher syndrome, type 2A (OMIM#276901) | Two heterozygous variants | USH2A; chr1:[216498735G>A](;)[215848045_215848046del]; NM_206933.2:c.[1055C>T](;)[13207_13208del]; NP_996816.2:p.[(Thr352Ile)](;)[(Gly4403ProfsTer15)] | Rod-cone dystrophy | RP | N.D. | Rod-cone dystrophy | Possible Molecular Diagnosis |
| 262 | M | 69 | proband | 2 likely pathogenic variants and 1 variant of uncertain significance (phase unknown) | AR, Usher syndrome, Type 2A (OMIM#276901), AR, Retinitis pigmentosa 39 (OMIM#613809) | three heterozygous variants | USH2A; chr1:[216243634G>C](;)[215848678C>T](;)[216498790G>A]; NM_206933.2:c.[5858C>G](;)[12575G>A](;)[1000C>T]; NP_996816.2:p.[(Ala1953Gly)](;)[(Arg4192His)](;)[(Arg334Trp)] | Rod-cone dystrophy | RP | N.D. | Rod-cone dystrophy | Possible Molecular Diagnosis |
| 263 | M | 35 | proband, mother | VOUS (Heterozygous) | Retinitis pigmentosa 48 (OMIM#613827) | single heterozygous variant in Autosomal Dominant disorder | GUCA1B; Chr6:[42162404_42162406del];[=]; NM_002098.5:c.[153_155delTGA];[=]; NP_002089.4:p.[(Asp51del)];[=] | Rod-cone dystrophy | RP (severe) | Rod-cone | Rod-cone dystrophy | Possible Molecular Diagnosis |
| 264 | F | 39 | proband | 2 Variants of Uncertain Significance (Heterozygous) | Cone-rod dystrophy 3 (OMIM# 604116); Autosomal Recessive Fundus flavimaculatus (OMIM# 248200); Autosomal Recessive Retinal dystrophy, early-onset severe (OMIM# 248200); Autosomal Recessive Retinitis pigmentosa 19 (OMIM# 601718); Autosomal Recessive Stargardt disease 1 (OMIM# 248200) | Two heterozygous variants, phase unknown | ABCA4; Chr1:[94502837C>T](;)[94463398_94463412del]; NM_000350.2:c.[3677G>A](;)[6729+5_6729+19del]; NP_000341.2:p.[(Gly1226Asp)](;)[?] | Maculopathy 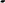 | Bull's eye maculopathy 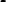 | Normal: No detectable dysfunction | Macular dystrophy | Possible Molecular Diagnosis |
| 265*^^#7^ | M | 57 | proband | VOUS (Heterozygous) | autosomal dominant retinitis pigmentosa (RP) with features of chroideremia, based on Bowne et al. 2011; Hull et al. 2016 | Heterozygous | RPE65; Chr:[68896768T>C];[=]; NM_000329.2:c.[1430A>G];[=]; NP_000320.1:p.[(Asp477Gly)];[=] | Maculopathy | Maternally-inherited diabetes and deafness (MIDD) | Rod-cone | Macular dystrophy | Possible Molecular Diagnosis |
| 266 | F | 77 | proband | heterozygous; 1 variant of unknown significance in a AD gene; 2 Variants of unknown significance identified in an AR gene, Phase unknown | PRPH2: Choroidal dystrophy, central areolar 2 (OMIM#613105), Leber congenital amaurosis 18 (OMIM#608133), Macular dystrophy, patterned, 1 (OMIM#169150), Macular dystrophy, vitelliform, 3 (OMIM#608161), Retinitis pigmentosa 7 and digenic (OMIM#608133) Retinitis punctata albescens (OMIM#136880) ADGRV1:Febrile seizures, familial, 4 (OMIM#604352); Usher syndrome, type 2C (OMIM#605472); Usher syndrome type 2C, GPR98/PDZD7 digenic (OMIM#605472) | Heterozygous | PRPH2; Chr6:[42689802A>T];[=]; NM_000322.4:c.[271T>A];[=]; NP_000313.2:p.[Tyr91Asn];[=]  ADGRV1; Chr5:[89910831G>T](;)[89968466G>A]; NM_032119.3:c.[202G>T](;)[4856G>A]; NP_115495.3:p.[(Val68Leu)](;)[(Gly1619Asp)] | Cone-rod dystrophy 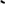 | N.D. | Cone-rod | Cone-rod dystrophy | Possible Molecular Diagnosis |
| 267 | F | 59 | proband | Variant of Unknown Significance identified | PRPH2: Choroidal dystrophy, central areolar 2 (OMIM#613105), Leber congenital amaurosis 18 (OMIM#608133), Macular dystrophy, patterned, 1 (OMIM#169150), Macular dystrophy, vitelliform, 3 (OMIM#608161), Retinitis pigmentosa 7 and digenic (OMIM#608133) Retinitis punctata albescens (OMIM#136880) | Heterozygous | PRPH2; Chr6:[42689525G>A];[=]; NM_000322.4:c.[548G>A];[=]; NP_000313.2:p.[Arg183His];[=] | Maculopathy vs. Cone-rod dystrophy | Bull's eye maculopathy | Amplitudinal decreases in both rod and cone responses | Macular dystrophy | Possible Molecular Diagnosis |
| 268 | M | 59 | proband | Variant of Uncertain Significance identified | Autosomal Dominant Retinitis Pigmentosa 31 (OMIM#609923) | Heterozygous | TOPORS; Chr9:[32542492A>C];[=]; NM_005802.4:c.[2031T>G];[=]; NP_005793.2:p.[His677Gln];[=] | Rod-cone dystrophy | RP (atypical) | Non-recordable responses | Rod-cone dystrophy | Possible Molecular Diagnosis |
| 269 | M | 68 | proband | One Heterozygous Variant of Unknown Significance Identified | Autosomal Dominant Retinitis Pigmentosa (OMIM#180105) | Heterozygous | IMPDH1; Chr7:[128035310G>A];[=]; NM_000883.3:c.[1280C>T];[=]; NP_000874.2:p.[Pro427Leu];[=] | Rod-cone dystrophy | RP | Non-recordable responses | Rod-cone dystrophy | Possible Molecular Diagnosis |
| 270 | F | 84 | proband;C5: (Daughter);C6: (Son) | One Heterozygous Variant of Unknown Significance identified; also found in the patient’s daughter and the patient’s son who are both asymptomatic | Autosomal Recessive and Autosomal Dominant Cone-rod dystrophy 6 (OMIM# 601777) | Heterozygous | GUCY2D; Chr17:[7906612C>A];[=]; NM_000180.3:c.[247C>A];[=]; NP_000171.1:p.[Arg83Ser];[=] | Rod-cone dystrophy | RP (atypical) | Non-recordable responses | Rod-cone dystrophy | Other |
| 271 | F | 67 | proband;C3(SON) | Two variants of uncertain significance, in trans, identified | Usher Syndrome, type 2C (AR, OMIM#605472) | compound heterozygous | ADGRV1; Chr5:[89921028G>T];[89949562G>A]; NM_032119.3:c.[640G>T];[4171G>A]; NP_115495.3:p.[(Val214Leu)];[(Glu1391Lys)] | Rod-cone dystrophy | RP | Non-recordable responses | Rod-cone dystrophy | Possible Molecular Diagnosis |
| 272^#8^ | M | 32 | proband | Variant of Uncertain Significance | Autosomal recessive Retinitis pigmentosa 77 (MIM# 617304) | Homozygous | REEP6; chr19:[1495553G>A];[1495553G>A]; NM_138393.3:c.[295G>A];[295G>A]; NP_001316485.1:p.[Glu99Lys];[Glu99Lys] | Rod-cone dystrophy | RP (atypical) | Non-recordable responses | Rod-cone dystrophy | Possible Molecular Diagnosis |
| 273 | M | 92 | P;C3 (DAUGHTER) | Variant of uncertain clinical significance | Autosomal recessive achromatopsia3 (MIM#26300); macular degeneration, juvenile(MIM#248200). | Homozygous | CNGB3; Chr8:[87645110C>T];[87645110C>T]; NM_019098.4:c.[1190G>A];[1190G>A]; NP_061971.3:p.[(Cys397Tyr)];[(Cys397Tyr)] | Cone dystrophy | Achromatopsia | Cone | Cone dystrophy | Possible Molecular Diagnosis |
| 274 | M | 44 | proband | Two Heterozygous Variants of Unknown Significance Identified, Phase Undetermined | Autosomal Recessive Usher syndrome, type 2A (OMIM 276901), and Autosomal Recessive Retinitis Pigmentosa (OMIM 613809) | Two heterozygous variants, phase undetermined. | USH2A; Chr1:[216595222](;)[215960057C>T]; NM_206933.2:c.[457T>A](;)[10342G>A]; NP_996816.2:p.[(Trp153Arg)(;)[(Glu3448Lys)] | Rod-cone dystrophy | RP | Non-recordable responses | Rod-cone dystrophy | Possible Molecular Diagnosis |
| 275^#5^ | F | 78 | proband | Variant of Uncertain Significance Identified | Choroideremia (OMIM#303100) | Heterozygous | CHM; ChrX:[85282510A>T];[=]; NM_000390.3:c.[101T>A];[=]; NP_000381.1:p.[(Val34Asp)];[=] | Rod-cone dystrophy | CHM | Rod-cone | Rod-cone dystrophy | Possible Molecular Diagnosis |
| 276 | M | 66 | proband | Variant of Uncertain Significance identified | autosomal dominant glaucoma 1, open angle, f (OMIM#603383) | Heterozygous | ASB10; Chr7:[150883614G>T];[=]; NM_001142459.1:c.[449C>A];[=]; NP_001135931.2:p.[(Thr150Asn)];[=] | Glaucoma | N.D. | Normal: No detectable dysfunction | Other | Possible Molecular Diagnosis |
| 277*^ | F | 70 | proband | One Variant of Uncertain Significance identified | Retinitis pigmentosa 1; Autosomal Dominant (OMIM# 180100) | Heterozygous | RP1; Chr 8:[55534144G>A];[=]; NM_006269.1:c.[615+3G>A];[=] | Rod-cone dystrophy | RP (atypical) | Non-recordable responses | Rod-cone dystrophy | Possible Molecular Diagnosis |
| 278 | M | 54 | proband | One heterozygous variant of uncertain significance related to patient’s phenotype identified. | Leber congenital amaurosis 11 (OMIM#613837), Retinitis pigmentosa 10 (AD, OMIM#180105) | Heterozygous | IMPDH1; Chr7:[128040170T>A];[=]; NM_000883.3:c.[853A>T];[=]; NP_000874.2:p.[(Ile285Phe)];[=] | Unilateral CME/lamellar hole (OD) | N.D. | Normal: No detectable dysfunction | Other | Possible Molecular Diagnosis |
| 279 | M | 82 | proband | One Variant of Uncertain Significance identified | Retinitis pigmentosa 4, autosomal dominant or recessive (OMIM#613731); Retinitis punctata albescens, autosomal dominant or recessive (OMIM#136880). | Heterozygous | RHO; Chr3:[129247659A>G];[=]; NM_000539.3:c.[83A>G];[=]; NP_000530.1:p.[(Gln28Arg)];[=] | Rod-cone dystrophy | RP (atypical) | N.D. | Rod-cone dystrophy | Possible Molecular Diagnosis |
| 280 | F | 66 | proband | Two heterozygous VOUS, phase unknown | Autosomal Recessive Usher syndrome, type IV (OMIM#: 618144) | two heterozygous variants, phase unknown | ARSG; Chr17:[66381226C>T](;)[66416350del]; NM_014960.4:c.[1004C>T](;)[1326delG]; NP_055775.2:p.[(Thr335Met)](;)[(Ser443Alafs*12)] | Rod-cone dystrophy | RP (atypical) | Extinguished rod and cone responses | Rod-cone dystrophy | Possible Molecular Diagnosis |
| 281 | M | 64 | proband | Two heterozygous VOUS, phase unknown | Usher syndrome, Type 2A (Autosomal Recessive; OMIM#276901) | two heterozygous variants, phase unknown | USH2A; Chr1:[21659540A>C](;)[215916561G>A]; NM_206933.2:c.[274T>G](;)[11506C>T]; NP_996816.2:p.[(Ser92Ala)](;)[(Pro3836Ser)] | Rod-cone dystrophy | US | N.D. | Rod-cone dystrophy | Possible Molecular Diagnosis |
| 282 | M | 65 | proband | Heterozygous VOUS | Cone-rod dystrophy 6 (AD,AR, OMIM#601777); Leber Congenital Amaurosis 1 (AR,OMIM#204000) | Heterozygous | GUCY2D; Chr17:[7918022C>T];[=]; NM_000180.3:c.[2516C>T];[=]; NP_0001711:p.[(Thr839Met)];[=] | Cone dystrophy | N.D. | Cone | Cone dystrophy | Possible Molecular Diagnosis |
| 283 | F | 56 | proband | Heterozygous VOUS | AD, AR, Fundus Albipunctatus (OMIM: 136880); AR Bothnia retinal dystrophy (OMIM: 607475);Newfoundland rod-cone dystrophy (OMIM: 607476);AD,AR,Retinitis punctate albescens(OMIM:136880) | Heterozygous | RLBP1; Chr15:[89758376A>G];[=]; NM_000326.4:c.[440T>C];[=]; NP_000317.1:p.[(Val147Ala)];[=] | Maculopathy | Pattern dystrophy | Normal: No detectable dysfunction* | Macular dystrophy | Possible Molecular Diagnosis |
| 284 | M | 39 | proband | Two variants of uncertain significance identified, phase unknown | Retinitis pigmentosa 39 (OMIM#: 613809); Usher syndrome, type 2A (OMIM#: 276901) | Two heterozygous variants, phase unknown | USH2A; Chr1:[215848910G>A](;)[215847979G>A]; NM_206933.2:c.[12343C>T](;)[13274C>T]; NP_996816.2:p.[(Arg4115Cys)](;) [(Thr4425Met)] | Rod-cone dystrophy | RP (severe) | Rod-cone | Rod-cone dystrophy | Possible Molecular Diagnosis |
| 285 | M | 83 | proband | One Variant of Uncertain Significance identified | Autosomal Dominant and Autosomal Recessive Retinitis Pigmentosa 1(OMIM#180100) | Heterozygous | RP1; Chr8:[55537952T>G];[=]; NM_006269.1:c.[1510T>G];[=]; NP_006260.1p.[(Ser504Ala)];[=] | Macular posterior epithelial detachments | N.D. | Cone-rod | Cone-rod dystrophy | Possible Molecular Diagnosis |
| 286 | F | 39 | proband | Two variants of uncertain significance of unknown phase in EYS, and a single heterozygous pathogenic variant in an autosomal recessive disease gene, CNGA1 identified | Autosomal Recessive Retinitis pigmentosa 25 (OMIM# 602772) | two heterozygous variants, phase unknown; heterozygous | EYS; Chr6:[66044979A>T](;)[66204564G>A]; NM_001142800.1:c.[740C>T](;)[1660T>A]; NP_001136272.1:p.[(Pro247Leu)](;)[(Cys554Ser)] | Cone dystrophy vs. Cone-rod dystrophy vs. Maculopathy | N.D. | Cone | Other | Possible Molecular Diagnosis |
| 287 | F | 47 | proband + C3,C4 (sons) | Two Homozygous Variants of Uncertain Significance in EYS identified | Autosomal Recessive Retinitis pigmentosa 25 (OMIM# 602772) | 2 Homozygous | EYS; Chr6:[64574166C>T;64430907_644309115del];[64574166C>T;64430907_644309115del], NM_001142800.2:c.[7141G>A;9012_9020del];[7141G>A;9012_9020del]; NP_001136272.1:p.[(Gly2381Arg);(Glu3004_Asn3006del)];[(Gly2381Arg);(Glu3004_Asn3006del)] | Rod-cone dystrophy | Sectoral RP | Both rod and cone attenuation | Rod-cone dystrophy | Possible Molecular Diagnosis |
| 288 | M | 39 | proband | Variant of Uncertain Significance identified | Cone-rod dystrophy 6, AD, AR (OMIM#601777); Leber congenital amaurosis 1, AR (OMIM#204000) | Heterozygous | GUCY2D; Chr17:[7918012C>T];[=]; NM_000180.3:c.[2506C>T];[=]; NP_000171.1:p.[(Arg836Trp)];[=] | Cone-rod dystrophy vs. Maculopathy | Pattern dystrophy | Both rod and cone attenuation | Other | Possible Molecular Diagnosis |
| 289 | F | 33 | proband | Variant of Uncertain Significance identified | Vitelliform macular dystrophy 5 (OMIM#616152), Retinitis Pigmentosa 56 (OMIM#613581) | heterozygous | IMPG2; Chr3:[100963029A>G];[=]; NM_016247.3:c.[2146T>C];[=]; NP_057331.2:p.[(Ser716Pro)];[=] | Healthy | N.D. | N.D. | Other | Possible Molecular Diagnosis |
| 290 | M | 56 | proband | Heterozygous Variant of Unknown Significance Identified in X-linked gene | Retinitis Pigmentosa 3 (RP3; OMIM:300029), X-linked. Retinitis Pigmentosa, X-linked, and sinorespiratory infections, with or without deafness (OMIM:300455), Macular Degeneration, X-linked (OMIM:300834), Cone-rod dystrophy, X-linked (OMIM:304020). | Hemizygous. | RPGR; ChrX:[38146026_38146049];[0]; NM_001034853.1:c.[2203_2226del];[0]; NP_001030025.1:p.[(His735_Glu742del)];[0] | Rod-cone dystrophy | RP (severe) | N.D. | Rod-cone dystrophy | Possible Molecular Diagnosis |
| 291 | F | 76 | proband | Variant of uncertain significance identified in a clinically relevant gene. | Macular dystrophy, vitelliform, 5 (OMIM#616152, AD), Retinitis pigmentosa 56(OMIM#613581,AR)​ | heterozygous | IMPG2; Chr3:[100963388A>T];[=]; NM_016247.3:c.[1787T>A];[=]; NP_057331:p.[(Ile596Lys)];[=] | Maculopathy | N.D. | Normal: No detectable dysfunction | Macular dystrophy | Possible Molecular Diagnosis |
| 292 | F | 55 | proband, mother | 1 Homozygous Variant of Uncertain Significance identified | Autosomal Recessive Cone-rod dystrophy and hearing loss (OMIM#617236) | Homozygous (apparent) | CEP78; Chr9:[80877900G>A];[80877900G>A]; NM_001098802.2:c.[1461G>A];[1461G>A]; NP_001092272.1:p.[(Glu487=)];[(Glu487=)](;) | Rod-cone dystrophy | RP | Rod-cone | Rod-cone dystrophy | Possible Molecular Diagnosis |
| 293 | F | 15 | TRIO | Two variants of uncertain significance, in trans, identified in a gene related to the patient’s phenotype | Bothnia retinal dystrophy (AR; OMIM#607475), Fundus albipunctatus (AD, AR, OMIM#136880),Retinitis punctate albescens (AD, AR, OMIM#136880) | Compound heterozygous | RLBP1; Chr15:89760400_89760411del];[89761912G>A]; NM_000326.4:c.[286_297del];[25C>T]; NP_000317.1:p.[(Phe96_Phe99)];[(Arg9Cys)] | Vitamin A deficiency | N.D. | Rod-cone | Rod-cone dystrophy | Possible Molecular Diagnosis |
| 294 | M | 75 | proband | One heterozygous variant of uncertain significance identified | Wagner syndrome 1(OMIM#143200,AD) | Heterozygous | VCAN; Chr5:82835599_82835601del; NM_004385.4:c.[6782_6784del];[=]; NP_004376.2:p.[(Glu2261del)];[=] | Rod-cone dystrophy | RP (atypical) | N.D. | Rod-cone dystrophy | Possible Molecular Diagnosis |
| 295 | F | 37 | proband | Variant of Uncertain Significance identified. | Arts syndrome (XLR; OMIM#301835), Charcot-Marie-Tooth disease, X-linked recessive 5 (XLR; OMIM#311070), Deafness, X-Linked 1 (XL; OMIM# 304500), Gout, PRPS-related (XLR; OMIM# 300661), Phosphoribosylpyrophosphate synthetase superactivity (XLR; OMIM# 300661) | Heterozygous | PRPS1 ChrX:[106888549A>G];[=]; NM_002764.4:c.[673A>G];[=]; NP_002755.1:p.[Thr225Ala];[=] | RPGR carrier | N.D. | Normal: No detectable dysfunction | Rod-cone dystrophy | Possible Molecular Diagnosis |
| 296 | M | 69 | proband | Homozygous variant of uncertain significance identified in a clinically relevant gene | Retinitis pigmentosa-12 (OMIM#600105,AR); Leber congenital amaurosis 8 (OMIM#613835,AR); Pigmented paravenous chorioretinal atrophy (OMIM#172870,AD). | homozygous | CRB1; Chr1:[197396961C>A];[197396961C>A]; NM_201253.2:c.[2506C>A];[2506C>A]; NP_957705.1:p.[(Pro836Thr)];[(Pro836Thr)] | Rod-cone dystrophy | RP (atypical) | Non-recordable responses | Rod-cone dystrophy | Possible Molecular Diagnosis |
| 297 | M | 48 | proband, mother | Two Heterozygous Variants of Uncertain Significance Identified | susceptibility to age-related macular degeneration-1 (OMIM# 603075) | Heterozygous variants in cis | HMCN1; Chr1:[186086704A>T;186107069T>C];[=]; NM_031935.2:c.[11797A>T;13889T>C];[=]; NP_114141.2:p.[Asn3933Tyr;Val4630Ala];[=] | Cone-rod dystrophy vs. Maculopathy | N.D. | Cone-rod | Cone-rod dystrophy | Possible Molecular Diagnosis |
| 298 | F | 71 | proband | Heterozygous variant of uncertain significance identified in a clinically relevant gene | Enhanced S-cone syndrome (OMIM#268100,AR), Retinitis pigmentosa 37(OMIM#611131, AD,AR) | heterozygous | NR2E3; Chr15:[72104123G>T];[=]; NM_016346.3:c.[263G>T];[=]; NP_057430.1:p.[(Gly88Val)];[=] | Healthy | N.D. | Normal: No detectable dysfunction | Other | Possible Molecular Diagnosis |
| 299 | F | 54 | proband | 1 Likely Pathogenic variant and 1 variant of Uncertain Significance (phase unknown) | AR, Cone dystrophy 4 (OMIM# 613093); AR, Achromatopsia 5 (OMIM# 613093) | Two heterozygous variants | PDE6C; Chr10:[95421855G>C](;)[95422335_95422336del]; NM_006204.3:c.[2248G>C](;)[2304_2305del]; NP_006195.3:p.[(Asp750His)](;)[(Asp770*)] | Maculopathy | Bull's eye maculopathy | Cone-rod | Macular dystrophy | Possible Molecular Diagnosis |
| 300 | M | 52 | proband | 1 Likely Pathogenic Variant associated with autosomal recessive disease identified | AR Achromatopsia 3 (OMIM#262300); AR Macular degeneration, juvenile (OMIM#248200) | 1 Heterozygous | CNGB3; Chr8:[87641222A>C];[=]; NM_019098.4:c.[1405T>G];[=]; NP_061971.3:p.[(Tyr469Asp)];[=] | Rod-cone dystrophy | RP (severe) | Non-recordable responses | Rod-cone dystrophy | Other |
| 301 | F | 66 | proband | 1 pathogenic variant and 1variant of uncertain significance identified ( phase unknown) | AR, Retinitis Pigmentosa 80 (OMIM#617781); AR, short-rib thoracic dysplasia 9 with or without polydactyly (OMIM#266920) | two heterozygous variants | IFT140; Chr16:[1607935C>A](;)[1642520_1642522del]; NM_014714.3:c.[437_439del](;)[2399+1G>T]; NP_055529.2:p.[(His146_Glu147delinsGln)](;)[(?)] | Rod-cone dystrophy | RP (atypical) | Non-recordable responses | Rod-cone dystrophy | Possible Molecular Diagnosis |
| 302 | F | 55 | proband | 1 Likely Pathogenic Variant and 1 Variant of Uncertain Significance identified (phase unknown) | AR, Cone-rod dystrophy 3 (OMIM# 604116); AR, Fundus flavimaculatus (OMIM#248200); AR, Retinal dystrophy, early-onset severe (OMIM# 248200); AR, Retinitis pigmentosa 19 (OMIM# 601718); AR, Stargardt disease 1 (OMIM# 248200) | Two heterozygous variants | ABCA4; Chr1:[94512491C>T](;)[94466624C>T]; NM_000350.2:c.[2902G>A](;)[6320G>A]; NP_000341.2:p.[(Gly968Arg)](;)[(Arg2107His)] | Cone-rod dystrophy | STGD | N.D. | Cone-rod dystrophy | Possible Molecular Diagnosis |
| 303 | M | 64 | proband | 1 LP and 1VOUS, phase unknown | AR, Retinitis Pigmentosa 39 (OMIM:613809), AR, Usher Syndrome Type 2A (OMIM:276901) | Two heterozygous variants | USH2A; Chr1:[216497592C>T](;)[215848678C>T]; NM_206933.2:c.[1246G>A](;)[12575G>A]; NP_996816.2:p.[(Ala416Thr)](;)[(Arg4192His)] | Rod-cone dystrophy | RP (severe) | Rod-cone | Rod-cone dystrophy | Possible Molecular Diagnosis |
| 304 | F | 36 | proband | 1 Likely pathogenic variant and 1 VOUS, (phase unknown) | Deafness, autosomal dominant 11 (OMIM# 601317); Deafness, autosomal recessive 2 (OMIM#600060); AR, Usher syndrome, type 1B (OMIM# 276900). | Two heterozygous variants | MYO7A; chr11:[76871317G>A](;)[76886511G>A]; NM_000260.3:c.[1189G>A](;)[2187+1G>A]; NP_000251.3:p.[(Ala397Thr)](;)[?] | Rod-cone dystrophy | RP (atypical) | Rod-cone | Rod-cone dystrophy | Possible Molecular Diagnosis |
| 305 | M | 47 | proband | 1 Likely pathogenic variant and 1 VOUS, (phase unknown) | Night blindness, congenital stationary (AD, OMIM#163500), Retinitis Pigmentosa-40 (AR,OMIM#613801) | Two heterozygous variants | PDE6B; Chr4:[647684AC>A](;)[660383G>A]; NM_000283.3:c.[756del](;)[2332G>A]; NP_000274.2:p.[(Asp252Glufs*29)](;)[Val778Met] | Rod-cone dystrophy | RP (severe) | Rod-cone | Rod-cone dystrophy | Possible Molecular Diagnosis |
| 306 | M | 68 | proband + C3(Daughter) + C4 (Son) | 1 Likely pathogenic variant and 1 VOUS, (phase unknown) | AR, Retinitis pigmentosa 39 (OMIM# 613809); AR, Usher syndrome, type 2A (OMIM#276901) | Two heterozygous variants | USH2A; Chr1:[216373096A>T](;)[216143987C>A]; NM_206933.2:c.[3684T>A](;)[6937G>T]; NP_996816.2:p.[(Cys1228Ter)](;)[(Gly2313Cys)] | Rod-cone dystrophy | RP (severe) | N.D. | Rod-cone dystrophy | Possible Molecular Diagnosis |
| 307 | F | 37 | proband + C1+ C3 (uncle) | 1 likely pathogenic and 1 variant of uncertain significance (phase unknown) | AR, Cone-rod dystrophy 3 (OMIM# 604116), AR, Fundus flavimaculatus (OMIM# 248200), AR, Retinal dystrophy, early-onset severe (OMIM# 248200), AR, Retinitis pigmentosa 19 (OMIM#601718), AR, Stargardt disease1(OMIM#248200), AD, {Macular degeneration, age-related, 2} (OMIM#153800) | Two heterozygous variants | ABCA4; Chr1:[94466422C>T](;)[94480208A>C]; NM_000350.2:c.[6449G>A](;)[5351T>G]; NP_000341.2:p.[(Cys2150Tyr)](;)[(Leu1784Arg)] | Maculopathy | STGD | Normal: No detectable dysfunction | Macular dystrophy | Possible Molecular Diagnosis |
| 308 | M | 62 | proband + C3 (son) | 1 Likely Pathogenic variant identified in an autosomal recessive disease gene | AD, Macular dystrophy, vitelliform, 5 (OMIM#: 616152), AR, Retinitis pigmentosa 56 (OMIM#: 613581) | Heterozygous | IMPG2; Chr3:[100961687dup];[=]; NM_016247.4:c.[2867dup];[=]; NP_057331.2:p.[(Asn956Lysfs*19)];[=] | Rod-cone dystrophy | RP | Non-recordable responses | Rod-cone dystrophy | Other |
| 309 | M | 78 | proband | 1 Heterozygous pathogenic variant identified in an autosomal recessive disease gene | AR, Retinitis pigmentosa 25 ( OMIM#: 602772) | Heterozygous | EYS; Chr6:[65707474C>T];[=]; NM_001142800.1:c.[2259+1G>A];[=] | Rod-cone dystrophy | RP | Non-recordable responses | Rod-cone dystrophy | Other |
| 310 | F | 79 | proband | 1 Likely Pathogenic Variant associated with autosomal recessive disease identified | AR, Cone-rod dystrophy 3 (OMIM#604116); AR, Fundus flavimaculatus (OMIM#248200); AR, Retinal dystrophy, early-onset severe (OMIM#248200); AR, Retinitis pigmentosa 19 (OMIM#601718); AR, Stargardt disease (OMIM#248200); AD, Macular degeneration (OMIM#153800) | Heterozygous | ABCA4; Chr1:[94476428G>A];[=]; NM_000350.2:c.[5642C>T];[=]; NP_000341.2:p.[(Ala1881Val)];[=] | Rod-cone dystrophy | RP | N.D. | Rod-cone dystrophy | Other |
| 311 | F | 61 | proband | 1 Likely Pathogenic variant identified in autosomal recessive retinitis pigmentosa | AR, Retinitis pigmentosa 39 (OMIM#613809);AR, Usher syndrome, type 2A  (OMIM#276901) | Heterozygous | USH2A; Chr1:[215987168dup];[=]; NM_206933.2:c.[9650dup];[=]; NP_996816:p.[(Arg3217Lysfs*27)];[=] | Rod-cone dystrophy | RP (severe) | Non-recordable responses | Rod-cone dystrophy | Other |
| 312^ | M | 16 | proband/C1/C3 (sister) | Negative |  |  |  | Maculopathy | PPRCA | Amplitudinal decreases in both rod and cone responses | Macular dystrophy | Negative |
| 313^ | M | 55 | proband | Negative |  |  |  | Maculopathy | Pattern dystrophy | Cone-rod | Cone-rod dystrophy | Negative |
| 314*^ | F | 51 | proband | Negative |  |  |  | Rod-cone dystrophy | RP (severe) | N.D. | Rod-cone dystrophy | Negative |
| 315 | F | 42 | proband | Negative |  |  |  | Healthy | N.D. | **Normal:** No detectable dysfunction | Rod-cone dystrophy | Negative |
| 316 | M | 78 | proband | Negative |  |  |  | Rod-cone dystrophy | RP (severe) | Non-recordable responses | Rod-cone dystrophy | Negative |
| 317 | F | 62 | proband | Negative |  |  |  | Rod-cone dystrophy | RP | Amplitudinal decreases in both rod and cone responses | Rod-cone dystrophy | Negative |
| 318 | M | 59 | proband | Negative |  |  |  | Cone-rod dystrophy vs maculopathy | N.D. | Cone-rod | Cone-rod dystrophy | Negative |
| 319 | M | 74 | P; C3 (Son, unaffected) | Negative |  |  |  | Rod-cone dystrophy | RP (atypical) | Rod-cone | Rod-cone dystrophy | Negative |
| 320 | F | 56 | P;C3 (Son) | Negative |  |  |  | Maculopathy | Pattern dystrophy | **Normal:** No detectable dysfunction* | Macular dystrophy | Negative |
| 321 | M | 8 | P; C1;C3 ( Affected Brother) | Negative |  |  |  | Maculopathy | X-linked retinoschisis | N.D. | Macular dystrophy | Negative |
| 322^ | M | 44 | proband, mother | Negative |  |  |  | Cone-rod dystrophy vs rod-cone dystrophy | N.D. | Non-recordable responses | Rod-cone dystrophy | Negative |
| 323 | M | 38 | proband | Negative |  |  |  | Rod-cone dystrophy | RP (atypical) | rod-cone | Rod-cone dystrophy | Negative |
| 324 | M | 67 | proband | Negative |  |  |  | Rod-cone dystrophy | RP | Rod-cone | Rod-cone dystrophy | Negative |
| 325^ | M | 44 | proband, mother | Negative |  |  |  | Rod-cone dystrophy | RP | Non-recordable responses | Rod-cone dystrophy | Negative |
| 326 | F | 40 | proband | Negative |  |  |  | Rod-cone dystrophy | Unilateral RP | Rod-cone (right eye only) | Rod-cone dystrophy | Negative |
| 327 | F | 63 | proband | Negative |  |  |  | Rod-cone dystrophy | RP | Rod-cone | Rod-cone dystrophy | Negative |
| 328 | F | 48 | proband, mother | Negative |  |  |  | Cone-rod dystrophy | N.D. | N.D. | Cone-rod dystrophy | Negative |
| 329 | F | 67 | proband | Negative |  |  |  | Rod-cone dystrophy | RP | Cone | Rod-cone dystrophy | Negative |
| 330 | F | 73 | proband | Negative |  |  |  | Maculopathy | N.D. | **Normal:** No detectable dysfunction | Macular dystrophy | Negative |
| 331 | F | 3 m | TRIO | Negative |  |  |  | N.D. | N.D. | N.D. | Other | Negative |
| 332 | M | 44 | proband | Negative |  |  |  | Rod-cone dystrophy | Unilateral RP | Rod-cone (right eye only) | Rod-cone dystrophy | Negative |
| 333 | M | 18 | proband, mother | Negative |  |  |  | Maculopathy | N.D. | Moderate decrease in cone function | Macular dystrophy | Negative |
| 334 | M | 74 | proband | Negative |  |  |  | Rod-cone dystrophy | RP | Amplitudinal decreases in both rod and cone responses | Rod-cone dystrophy | Negative |
| 335 | M | 38 | P:C3(SON) | Negative |  |  |  | Rod-cone dystrophy | RP | Amplitudinal decreases in both rod and cone responses | Rod-cone dystrophy | Negative |
| 336 | M | 34 | proband | Negative |  |  |  | Rod-cone dystrophy | RP (severe) | Amplitudinal decreases in both rod and cone responses | Rod-cone dystrophy | Negative |
| 337 | M | 60 | proband | Negative |  |  |  | N.D. | N.D. | N.D. | Rod-cone dystrophy | Negative |
| 338 | M | 31 | proband | Negative |  |  |  | N.D. | N.D. | N.D. | Rod-cone dystrophy | Negative |
| 339 | M | 48 | proband | Negative |  |  |  | N.D. | N.D. | N.D. | Cone-rod dystrophy | Negative |
| 340 | M | 71 | proband + C3(son) +C4(daughter) | Negative |  |  |  | N.D. | N.D. | N.D. | Macular dystrophy | Negative |
| 341 | M | 61 | proband | Negative |  |  |  | N.D. | N.D. | N.D. | Other | Negative |
| 342 | F | 73 | proband | Negative |  |  |  | N.D. | N.D. | N.D. | Macular dystrophy | Negative |
| 343 | F | 46 | proband | Negative |  |  |  | N.D. | N.D. | N.D. | Rod-cone dystrophy | Negative |
| 344 | M | 75 | proband | Negative |  |  |  | N.D. | N.D. | N.D. | Macular dystrophy | Negative |
| 345 | F | 69 | proband | Negative |  |  |  | N.D. | N.D. | N.D. | Cone dystrophy | Negative |
| 346 | M | 70 | proband | Negative |  |  |  | N.D. | N.D. | N.D. | Cone dystrophy | Negative |
| 347 | M | 68 | proband | Negative |  |  |  | N.D. | N.D. | N.D. | Macular dystrophy | Negative |
| 348 | M | 53 | proband | Negative |  |  |  | N.D. | N.D. | N.D. | Rod-cone dystrophy | Negative |
| 349 | F | 70 | proband | Negative |  |  |  | N.D. | N.D. | N.D. | Rod-cone dystrophy | Negative |
| 350 | F | 69 | proband + C3 (Daughter) | Negative |  |  |  | N.D. | N.D. | N.D. | Rod-cone dystrophy | Negative |
| 351 | M | 38 | proband | Negative |  |  |  | N.D. | N.D. | N.D. | Rod-cone dystrophy | Negative |
| 352 | M | 42 | TRIO | Negative |  |  |  | N.D. | N.D. | N.D. | Cone dystrophy | Negative |
| 353 | M | 41 | proband | Negative |  |  |  | N.D. | N.D. | N.D. | Rod-cone dystrophy | Negative |
| 354 | F | 42 | proband | Negative |  |  |  | N.D. | N.D. | N.D. | Rod-cone dystrophy | Negative |
| 355 | F | 56 | proband | Negative |  |  |  | N.D. | N.D. | N.D. | Rod-cone dystrophy | Negative |
| 356 | F | 77 | proband | Negative |  |  |  | N.D. | N.D. | N.D. | Rod-cone dystrophy | Negative |
| 357 | F | 61 | proband | Likely pathogenic heterozygous variant in autosomal dominant disease related to retinoblastoma identified which is not the primary indication for the test | Retinoblastoma (OMIM#180200,AD,Smu) | heterozygous | RB1,Chr13:[49027221C>T;49027222C>T];[=], NM_000321.2:c.[1788_1789delCCinsTT];[=], NP_000312.2:p.[(Gln597Ter)];[=] | Rod-cone dystrophy | RP | N.D. | Rod-cone dystrophy | Negative |

*Sanger follow-up performed

^ Miseq RPGR ORF15 performed

#1 has been previously reported in Ophthalmic Genet. 2020 Feb;41(1):26-30. doi: 10.1080/13816810.2020.1723116. Epub 2020 Feb 13. PMID: 32052671

#2 has been previously reported in *Doc Ophthalmol. 2018 Apr;136(2):125-133. doi: 10.1007/s10633-018-9626-1. Epub 2018 Feb 6. PMID: 29411205; PMCID: PMC6015729.*

#3 has been previously reported in *Am J Med Genet A. 2019 Feb;179(2):312-316. doi: 10.1002/ajmg.a.61001. Epub 2018 Dec 18. PMID: 30561111; PMCID: PMC6349500.*

#4 has been previously reported in *Cold Spring Harb Mol Case Stud. 2019 Dec 13;5(6):a004481. doi: 10.1101/mcs.a004481. PMID: 31836589; PMCID: PMC6913139.*

#5 has been previously reported in *Am J Ophthalmol. 2019 Nov;207:77-86. doi: 10.1016/j.ajo.2019.06.002. Epub 2019 Jun 8. PMID: 31181178; PMCID: PMC7579725.*

#6 has been previously reported in *Orphanet J Rare Dis. 2020 Nov 13;15(1):320. doi: 10.1186/s13023-020-01600-8. PMID: 33187544; PMCID: PMC7662744.*

#7 has been previously reported in *Cold Spring Harb Mol Case Stud. 2020 Feb 3;6(1):a004952. doi: 10.1101/mcs.a004952. PMID: 32014860; PMCID: PMC6996519.*

#8 has been previously reported in *Doc Ophthalmol. 2020 Feb;140(1):67-75. doi: 10.1007/s10633-019-09719-1. Epub 2019 Sep 19. PMID: 31538292; PMCID: PMC7310602.*

AMD, Age-related Macular Degeneration; AZOOR, Acute zonal occult outer retinopathy; CHM, Choroideremia; ESCS, Enhanced S-Cone Syndrome; LCA, Leber Congenital Amaurosis; N.D., no data; OCA, Oculocutaneous Albinism; PPRCA, Pigmented Paravenous Retinochoroidal Atrophy; RP, Retinitis Pigmentosa; STGD, Stargardt disease; US ,Usher syndrome
