# Supplementary material for: Clinical exome sequencing for inherited retinal degenerations at a tertiary care center
Source: Sci Rep. 2022 Jun 7;12:9358. doi: 10.1038/s41598-022-13026-2 (PMC9174483; doi:10.1038/s41598-022-13026-2)
Supplement: Supplementary file 4 — Supplementary Information 4. [file 41598_2022_13026_MOESM4_ESM.docx]

**Supplemental table 3:** Additional information in individuals with syndromic IRD ES findings.

| Case# | Sex | Age | OMIM Disease Association | Zygosity | Variant | Extra-Retinal phenotypes | Clinical Follow-up / Referrals / Outcome | Case Category |
| --- | --- | --- | --- | --- | --- | --- | --- | --- |
| 9 | F | 22 | AR Joubert syndrome 5 (MIM 610188); Leber congenital amaurosis 10(MIM 611755); Meckel AR syndrome 4 (MIM 611134); AR Senior-Loken syndrome 6(MIM 610189) | two heterozygous variants, phase unknown | CEP290; Chr12:[88524330T>A](;)[88535011G>A]; NM_025114.3:c.[74C>T](;)[508A>T]; NP_079390.3:p.[(Ala25Val)](;)[(Lys170Ter)] | No extra-retinal phenotype as she was assessed for LCA vs RP. There was some question of nystagmus but this was not found on exam | No referrals made. Lost to follow-up | Likely Molecular Diagnosis |
| 13 | F | 44 | AD Endosteal hyperostosis(MIM:144750); AD Osteopetrosis 1 (MIM:607634); AD Osteosclerosis, (MIM:144750); AD van Buchem disease type 2(OMIM 607636); AD Osteopetrosis, (OMIM 166710); AR Osteoporosis-pseudoglioma syndrome (OMIM 259770); AD/AR Exudative vitreoretinopathy 4(OMIM 601813) | Heterozygous | LRP5; Chr11:[68125147C>T];[68125147C>T]; NM_002335.3:c.[518C>T];[=]; NP_002326.2:p.[(Thr173Met)];[=] | Ptosis noted and patient was seen by oculoplastic. Treated for hypothyroidism (dx at 26). Patient self reported issues of night sweats and sinusitis. | No referrals made. Lost to follow-up | Possible Molecular Diagnosis |
| 16 | M | 41 | AR Nephronophthisis 4 (MIM 606966); AR Senior-Loken syndrome 4 (MIM 606996) | two heterozygous variants, phase unknown | NPHP4; Chr1:[5969253G>A](;)[5993384-5993385insGG], NM_015102.4:c.[1124_1125insCC](;)[1463C>T]; NP_055917.1:p.[(Ser376LeufsTer31)](;)[(Arg488Ter)] | Chronic stage 3 kidney disease, hyperparathyroidism (secondary), anemia of renal failure, hypertension | Renal specialist seen, renal transplant | Likely Molecular Diagnosis |
| 139 | M | 20 | AR, AD Exudative vitreoretinopathy 4 (MIM 601813); AD Hyperostosis, endosteal (MIM 144750); AD Osteopetrosis 1(MIM 607634); AR Osteoporosis-pseudoglioma syndrome (MIM259770); AD Osteosclerosis (MIM 144750); AD Polycystic liver disease 4 with or without kidney cysts (MIM 617875); AD van Buchem disease, type 2 (MIM 607636) | Heterozygous | LRP5:Chr11:[68183919A>G];[=]; NM_002335.3:c.[2951A>G];[=]; NP_002326.2:p.[(Tyr984Cys)];[=] | Nystagmus noted on exam | None | Possible Molecular Diagnosis |
| 142 | M | 11 | AR Usher syndrome, type 2C (MIM 605472); AR Usher syndrome, type 2C, GPR98/PDZD7 digenic (MIM 605472) | USH2A: compound heterozygous variants; GUS: SCAPER - homozygous | ADGRV1 Chr5:[90001231G>A];[90085544G>A]; NM_032119.3:c.[8401G>A];[13919G>A] NP_115495.3:p.[Gly2801Arg];[Gly4649Glu].  SCAPER Chr15:[77021080T>C];[77021080T>C]; NM_020843.4:c.[2023-2A>G];[2023-2A>G] | None noted | None | Possible Molecular Diagnosis |
|  |  |  |  |  |  |  |  |  |
| 144 | M | 2 | AR Joubert syndrome 5 (MIM 610188); Leber congenital amaurosis 10(MIM 611755); Meckel AR syndrome 4 (MIM 611134); AR Senior-Loken syndrome 6(MIM 610189) | Heterozygous | CEP290, Chr12:[88454772C>T];[=]; NM_025114.3:c.[6358-1G>A];[=] | Nystagmus and intermittent exotropia | Referral to audiology, normal hearing test | Other |
| 151 | F | 9 | AR Albinism, brown oculocutaneous (MIM 203200); AR Albinism, oculocutaneous, type II(MIM 203200) | Heterozygous | OCA2: Chr15:[28259941T>C];[=]; NM_000275.2:c.[1025A>G];[=], NP_000266.2:p.[Tyr342Cys];[=] | Ocular albinism noted, Lazy eye [patched]. Bone age study performed which was normal | None | Other |
| 155 | M | 37 | AR Joubert syndrome 5 (MIM 610188); Leber congenital amaurosis 10(MIM 611755); Meckel AR syndrome 4 (MIM 611134); AR Senior-Loken syndrome 6(MIM 610189) | Compound heterozygous variants | CEP290:Chr12:[88443069_88443073delCTTCT];[88508951delA]; NM_025114.3:c.[1833delT];[7328_7332delAGAAG]; NP_079390.3:p.[(Phe611fs)];[(Glu2443fs)] | Nystagmus, Polycystic kidney, Hypertension | Was already seeing nephrology, no additional referrals | Molecular Diagnosis |
| 203 | F | 34 | AR, DR Bardet-Biedl syndrome 1 (MIM 209900) | Homozygous | BBS1:Chr11:[66293652T>G];[66293652T>G]; NM_024649.4:c.[1169T>G];[1169T>G]; NP_078925:p.[(Met390Arg)];[(Met390Arg)] | Ocular migraines, Gall bladder removal, Diabetes, | No referrals made. Lost to follow-up | Molecular Diagnosis |
| 219 | F | 23 | Autosomal Recessive and Autosomal Dominant Pseudoxanthoma Elasticum (OMIM# 264800) | Homozygous | ABCC6; Chr16:[16256935G>A];[16256935G>A]; NM_001171.5:c.[3421C>T];[3421C>T]; NP_001162.4:p.[(Arg1141Ter)];[(Arg1141Ter)] | Endometriosis, Migraines | No referrals made. Lost to follow-up | Molecular Diagnosis |
| 220 | F | 61 | Autosomal Recessive and Autosomal Dominant Pseudoxanthoma Elasticum (OMIM# 264800) | Homozygous | ABCC6; Chr16:[16256935G>A];[16256935G>A]; NM_001171.5:c.[3421C>T];[3421C>T]; NP_001162.4:p.[(Arg1141Ter)];[(Arg1141Ter)] | Arthritis, Pre-diabetic, Tendonitis, Lymphedema, Thyroid disorder, | Was already seeing several specialists, no additional referrals made, lost to follow-up | Molecular Diagnosis |
| 221 | F | 27 | Autosomal Recessive Joubert syndrome 3 (OMIM#: 608629) | Homozygous | AHI1; Chr6:[135748398G>A];[135748398G>A]; NM_001134830.1:c.[2671C>T];[2671C>T]; NP_001128302.1:p.[(Arg891Ter)];[(Arg891Ter)] | GI issues, Mobility problems, Kidney failure, Motor problems, Intellectual disability, Possible ataxia | Was seeing nephrology, gastroenterology, endocrinology. No additional referrals made | Molecular Diagnosis |
| 227 | M | 25 | Coenzyme Q10 deficiency, primary, 1 (OMIM#609825) | Compound heterozygous | COQ2; Chr4:[84205780dupG];[84205692G>C]; NM_015697.7:c.[288dupC];[376C>G]; NP_056512.5:p.[(Ala97Argfs)];[(Arg126Gly)] | Kidney Failure | Nephrology | Molecular Diagnosis |
| 231 | F | 9 | X-linked recessive ocular albinism, type I, Nettleship-Falls type (OMIM#300500) | mosaic (VAF = 21.4%; 39/182 reads) | GPR143; ChrX:[9711715=/T>G];  NM_000273.2:c.[659-2=/A>C] | None noted | None | Molecular Diagnosis |
| 259 | M | 22 | AR, Knobloch syndrome, type 1 (OMIM#267750) | two heterozygous variants | COL18A1; chr21:[46895403_46895404del](;)[46917565dup]; NM_030582.3:c.[1292_1293delCT](;)[3213dupC]; NP_085059.2:p.[(Ser431Trpfs)](;)[(Gly1072Argfs)] | Nystagmus, Crohn's disease, Syncope, Pituitary issues | None | Possible Molecular Diagnosis |
| 295 | F | 37 | Arts syndrome (XLR; OMIM#301835), Charcot-Marie-Tooth disease, X-linked recessive 5 (XLR; OMIM#311070), Deafness, X-Linked 1 (XL; OMIM# 304500), Gout, PRPS-related (XLR; OMIM# 300661), Phosphoribosylpyrophosphate synthetase superactivity (XLR; OMIM# 300661) | Heterozygous | PRPS1 ChrX:[106888549A>G];[=]; NM_002764.4:c.[673A>G];[=]; NP_002755.1:p.[Thr225Ala];[=] | Hearing Loss | No referrals made. Hearing loss due to GJB6 | Possible Molecular Diagnosis |
| 301 | F | 66 | AR, Retinitis Pigmentosa 80 (OMIM#617781); AR, short-rib thoracic dysplasia 9 with or without polydactyly (OMIM#266920) | two heterozygous variants | IFT140; Chr16:[1607935C>A](;)[1642520_1642522del]; NM_014714.3:c.[437_439del](;)[2399+1G>T]; NP_055529.2:p.[(His146_Glu147delinsGln)](;)[(?)] | None noted | None | Possible Molecular Diagnosis |
